# Targeting *Mycobacterium tuberculosis*: The Role of Alkyl Substitution in Pyrazinamide Derivatives

**DOI:** 10.1021/acsomega.5c07249

**Published:** 2026-01-14

**Authors:** Martin Juhás, Ghada Bouz, Luping Pang, Stephen D. Weeks, Ondřej Jand́ourek, Klára Konečná, Pavla Paterová, Pavel Bárta, Martina Halířová, Marta Kučerová-Chlupáčová, Martin Doležal, Jan Zitko

**Affiliations:** † Faculty of Pharmacy in Hradec Králové, 69727 Charles University, Ak. Heyrovského 1203, Hradec Králové 500 03, Czech Republic; ‡ Faculty of Science, University of Hradec Králové, Rokitanského 62, Hradec Králové 500 03, Czech Republic; § Faculty of Pharmacy, University Business Academy, Heroja Pinkija 4, Novi Sad 21101, Serbia; ∥ Department of Medical Genetics and Cell Biology, School of Basic Medical Sciences, 12636 Zhengzhou University, Zhengzhou, Henan 450001, China; ⊥ Medicinal Chemistry, Rega Institute for Medical Research, KU Leuven, Herestraat 49—Box 1041, Leuven 3000, Belgium; # Pledge Therapeutics, Gaston Geenslaan 1, Leuven 3001, Belgium; ∇ Department of Clinical Microbiology, University Hospital Hradec Králové, Sokolská 581, Hradec Králové 500 05, Czech Republic

## Abstract

Tuberculosis (TB) remains a significant global health challenge due to the rapid emergence of drug resistance. Despite substantial progress in anti-TB drug development, effective treatment options are limited. In this study, we report the synthesis and biological evaluation of pyrazinamide (PZA) derivatives with 5-alkyl and 5-alkanamido modifications, designed to enhance antimycobacterial activity by increasing lipophilicity and improving penetration of the lipid-rich mycobacterial cell wall. A positive correlation between the length of the 5-alkyl chain and antimycobacterial activity was observed, with maximal potency achieved with the heptyl substituent (**4**: 5-heptylpyrazine-2-carboxamide, MIC_*M. tuberculosis* H37Rv = 3.13 μg/mL). In series C with phenyl substitution on the C-2 carboxamide, different simple substituents were tolerated on the benzene ring (both electron-donating and electron-withdrawing, both lipophilic and hydrophilic), and the length of the alkyl chain was the main determinant of the antimycobacterial activity. Compound **23** (5-hexyl-*N*-(3-trifluoromethylphenyl)­pyrazine-2-carboxamide) exerted MIC = 3.13 μg/mL and selectivity index (SI, compared to HepG2 cells) >25. Notably, the tested compounds exhibited significant activity against multidrug-resistant (MDR) *Mycobacterium tuberculosis* strains while maintaining favorable selectivity profiles and low cytotoxicity. In contrast, 5-alkanamido derivatives (series B and D) were devoid of antimycobacterial activity. Mechanistic investigations revealed that unlike PZA, the 5-alkyl pyrazinamide derivatives are not hydrolyzed by mycobacterial pyrazinamidase (PncA), indicating a distinct mode of action. While molecular modeling initially suggested enoyl-ACP reductase (InhA) as a potential target of series C, subsequent experimental validation disproved this hypothesis; thus, the precise mechanism of action remains to be elucidated.

## Graphical abstract

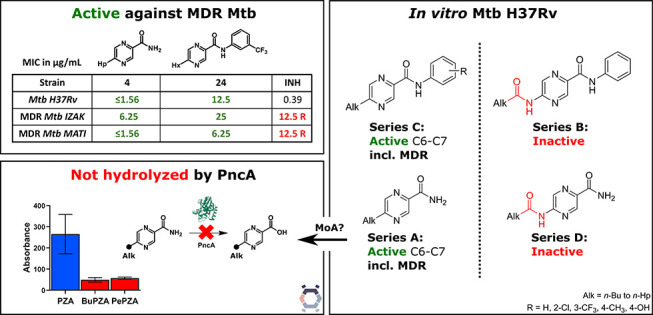

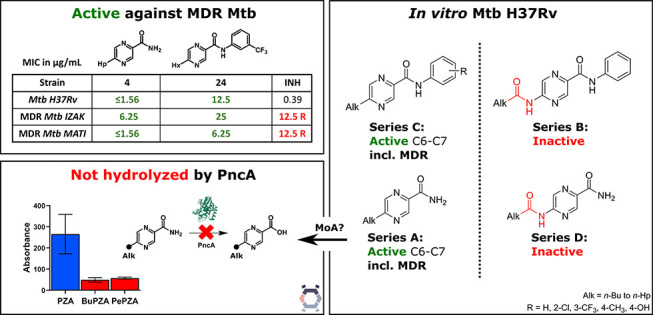

## 1 Introduction and Design Rationale

Tuberculosis (TB) is a lung infection predominantly caused by *Mycobacterium tuberculosis* (*Mtb*). TB infection can stay dormant for years, and the latent infection poses no apparent issues, while the active state of the disease endangers both affected individuals and society, as TB is highly contagious. Based on the most recent data from the World Health Organization (WHO), TB remains a significant global health threat. In 2023 alone, around 10.8 million people developed active TB, and 1.25 million people died from the infection, including 160,000 people with HIV coinfection. In addition, nearly half a million new cases of multidrug-resistant TB (MDR-TB) occur annually.^1^ MDR-TB is on the WHO list of priority pathogens, which should be addressed by antimicrobial research.^2^

The current treatment regimen for TB is complex, typically consisting of a combination of four anti-TB drugs for an extended period of time. In drug-resistant cases, the regimen is, however, much more complicated, often combining up to seven drugs for a duration exceeding a year.^3^ This combinatorial treatment regimen increases the risk of developing hepatotoxicity and/or neurotoxicity.^3^ The negatives of the TB treatment regimen result in low adherence and subsequently an increased emergence of resistance. The current antimycobacterial research employs new subcellular targets,^4^ including those associated with the pathogen’s defense against the host’s immune system.^5^

Pyrazinamide (PZA), a first-line antitubercular drug, has been in clinical practice since the 1950s. Despite this, its mechanism of action is still not fully understood. Next to purely nonspecific theories of action where the active metabolite pyrazinoic acid (POA) works as a protonophore,^6,7^ there are several specific subcellular targets associated with the action of PZA^8^ or its simple derivatives. As significant examples, 5-Cl-PZA is an inhibitor of mycobacterial Fatty Acid Synthase I complex,^9,10^ while POA and 6-Cl-POA inhibit the activity or trigger the degradation of mycobacterial aspartate decarboxylase (PanD).^11–13^ According to our CAS database search via SciFinder^n^ (November 2025, see Supporting Information, Section 1.7 for the query details), 962 simple derivatives of PZA were studied as antimycobacterial compounds, with structural changes focusing on substitution of the carboxamidic moiety^14^ and/or simple substituents on the pyrazine ring.^15^ Only morphazinamide (aka morinamide, *N*-(morpholinomethyl)­pyrazine-2-carboxamide)^16^ was registered but remained with little to no clinical usage.

In our research, we focus on developing derivatives of PZA to achieve novel active compounds with improved properties and potentially a new mechanism of action, addressing resistance. Mycobacteria possess a thick lipophilic cell wall rich in mycolic acids,^17^ which are fatty acids with extremely long alkyl chains (60–90 carbon atoms per molecule).^18^ Hence, we decided to investigate the influence of attaching an alkyl chain to the pyrazine core of PZA as an attempt to resemble the mycolic acid structure and enhance the penetration of such molecules into mycobacteria. Previously, we reported on the design, synthesis, and antimicrobial evaluation of derivatives with *N-*alkylamino substitution in positions 5 and 6 of the pyrazine core of PZA.^19–23^ In many of these homologues, we observed that increasing the lipophilicity of the derivatives often favors their whole-cell antimycobacterial activity.

In this paper, we report on the continuation of our efforts in preparing 5-alkyl- (series A) and 5-alkanamidopyrazine-2-carboxamides (series B) and their corresponding *N*-phenyl derivatives (series C and D, respectively). For proper evaluation of the structure–activity relationships, we also tested the corresponding free carboxylic acids of series A. For structures, refer to Figure 1. The terminal alkyl chain ranged from *n*-propyl to *n*-heptyl (in the paper abbreviated as Pr, Bu, Pe, Hx, and Hp). All prepared compounds were evaluated *in vitro* for their antimycobacterial activity against *M. tuberculosis* H37Rv, nontuberculous mycobacteria *M. kansasii* and *M. avium*, and *Mycolicibacterium smegmatis* and *M. aurum* (commonly applied surrogate model organisms in antimycobacterial research).^24^ Compounds that showed *in vitro* activity (here defined as MIC <25 μg/mL against *Mtb* H37Rv) were advanced into cytotoxicity screening in the HepG2 liver cancer cell line. Enoyl reductase InhA was speculated and experimentally tested as a possible mycobacterial molecular target for the title compounds. We also addressed the question of whether the derivatives with unsubstituted carboxamide can be substrates of mycobacterial pyrazinamidase (PncA), whose activity is a determinant of PZA susceptibility in mycobacteria.^25^ The obtained results were compared to previous works in order to draw structure–activity relationships (SAR) and recommendations that will direct future synthetic efforts.

**Figure 1:**
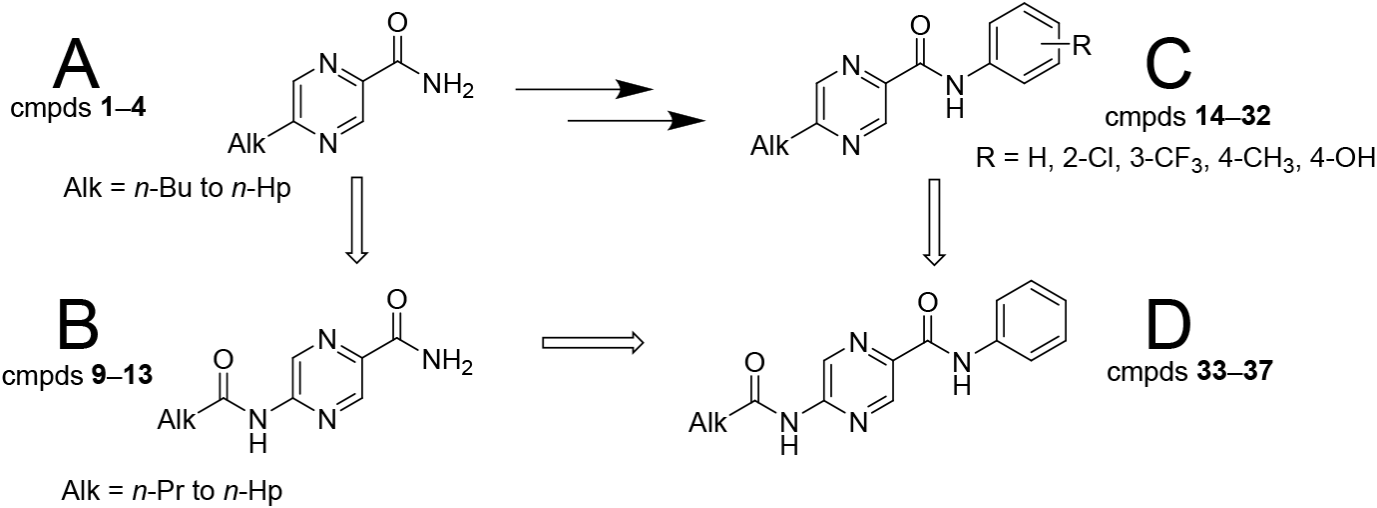
Chemical structures of the title compounds: (A) series A—5-alkyl derivatives **1**–**4**; (B) series B—5-alkanamido derivatives **9**–**13**; (C) series C—5-alkyl-*N*-phenyl derivatives **14**–**32;** and (D) series D—5-alkanamido-*N*-phenyl derivatives **33**–**37**. Compounds **5**–**8** are the corresponding carboxylic acids of compounds **1**–**4**.

## 2 Results and Discussion

### 2.1 Chemistry

5-Alkylpyrazine-2-carbonitriles (BuCN, PeCN, HxCN, and HpCN) were prepared with Minisci radical alkylation of pyrazine-2-carbonitrile according to a previously published procedure.^26,27^ The reaction was performed in water upon heating, the source of the alkyl radical was the corresponding alkanoic acid (one carbon longer than the intended alkyl to be introduced), and the radicals were produced using a silver nitrate/ammonium peroxydisulfate system (Scheme 1, a). The 5-alkylpyrazine-2-carbonitriles were isolated as viscous, colorless to slightly yellow liquids in moderate yields of 18–48%. The carbonitriles underwent partial hydrolysis (Scheme 1, b) using hydrogen peroxide and NaOH under strictly controlled pH = 9. The resulting 5-alkylpyrazine-2-carboxamides (**1**–**4**) were isolated as white solids in high yields of 82–92%. Finally, the corresponding 5-alkylpyrazine-2-carboxylic acids (**5**–**8**) were prepared with complete basic hydrolysis of the carboxamides (Scheme 1, c) and, after acidification, isolated as low-melting white solids in yields of 75–91%.

**Scheme 1:**
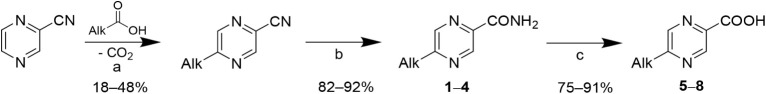
Synthesis of Simple Alkylated Derivatives^a^

a Reagents and conditions: a—Alk-COOH, AgNO_3_, (NH_4_)_2_S_2_O_8_, water, 80 °C; b—H_2_O_2_, NaOH, H_2_O, 55 °C, controlled pH = 9; c—NaOH (aq), products isolated as free acids after acidification.

5-Chloropyrazine-2-carboxamide (5-Cl-PZA, Scheme 2, **I**) was prepared from commercially available 5-hydroxypyrazinoic acid (5-OH-POA) following the previously published procedure.^19^ Briefly, 5-OH-POA was activated with thionyl chloride (SOCl_2_) with a catalytic amount of *N*,*N*-dimethylformamide (DMF) in anhydrous toluene. The solvents were evaporated, and repeated evaporation with toluene was used to remove excess SOCl_2_. The crude acyl chloride was dissolved in anhydrous dichloromethane (DCM), followed by the addition of aqueous ammonia (Scheme 2, a). 5-Cl-PZA precipitated from the reaction, and after a simple washing with cold water, it was isolated in 77% yield. The anilide of 5-chloropyrazinoic acid (5-Cl-POA-anilide, Scheme 2, **II**) was prepared analogously, following a previously described procedure,^28^ and was isolated in 70% yield.

**Scheme 2:**
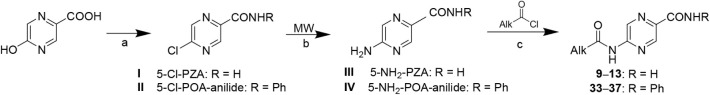
Synthesis of Alkanamido Derivatives[Fn sch2-fn2]

a Reagents and conditions: a—(1) SOCl_2_, DMF in anhydrous toluene, 110 °C, (2) NH_3_ (25% aq. sol.) in DCM for **I,** or aniline with TEA in DCM for **II**, for both ice bath to RT; b—NH_3_ (25% aq. sol): MeOH (3:1); MW (140 °C, 45 min, 60W); c—1.2 eq. of corresponding acyl chloride; 2 eq. of TEA, 2 h stirring in ice bath, then 2 h stirring at RT.

The substitution of the chlorine for the amino group (Scheme 2, b) was achieved using nucleophilic substitution of the corresponding chloro derivative (**I** or **II**) upon treatment with aqueous ammonia (25%) in MeOH (cosolvent) under microwave-assisted heating. The reaction was performed at 140 °C in a closed-vial system. The generated pressure (dependent on the volume of solvents) was approximately 7–8 bar. The closed system setup was convenient for keeping the volatile ammonia in the reaction. Earlier reported attempts to perform the reaction in boiling EtOH in an open atmosphere did not lead to satisfactory yields even with reaction times of several hours.^29^ The final alkanamido derivatives of PZA **9**–**13** and POA-anilide **33**–**37** were obtained using acylation with aliphatic acyl chlorides in yields of 32–58% and 42–80%, respectively.

Anilides of 5-alkylpyrazine-2-carboxylic acids (**14**–**32**) were prepared from 5-alkylpyrazinoic acids (**5**–**8**) using a standard 1,1′-carbonyldiimidazole (CDI) coupling reagent (Scheme 3) and isolated as solids with a typical yield 20–62%.

**Scheme 3:**
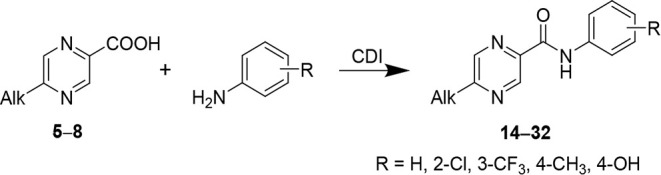
Synthesis of Anilides of 5-Alkylpyrazine-2-carboxylic acids (**14**–**32**) via CDI-Mediated Coupling

### 2.2 *In Vitro* Antimycobacterial Activity

All synthesized compounds, including isolated intermediates and the free acids (compounds **5**–**8**), were evaluated for *in vitro* antimycobacterial activity against *Mtb* H37Rv, *M. kansasii*, *M. avium*, *M. smegmatis*, and *M. aurum* using the Microplate Alamar Blue Assay. The antimycobacterial activity results were expressed as minimum inhibitory concentrations (MIC) in μg/mL; isoniazid (INH) and pyrazinamide (PZA) were used as standards. The results for *Mtb* H37Rv are presented in Table 1, along with the determined cytotoxicity for the human hepatocellular carcinoma cell line (HepG2) for the active compounds (defined as MIC < 25 μg/mL). TB treatment regimen is known to carry a significant risk of hepatotoxicity, and therefore HepG2 cell line—besides being the standard cell line for cytotoxicity evaluation—is of great value in our case.

**Table 1:**
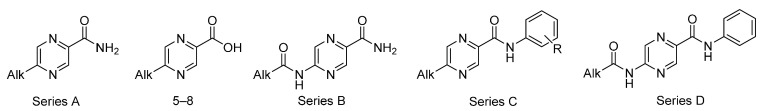
Prepared Compounds with Their Calculated Lipophilicity (log *P*), Antimycobacterial Activity against *Mtb* H37Rv Expressed as Minimum Inhibitory Concentration (MIC in μg/mL) Converted to μM in Parentheses, HepG2 Cytotoxicity Expressed as Half Maximal Inhibitory Concentration (IC_50_ in μM), and Selectivity Index (SI)^a^

					*Mtb* H37Rv	HepG2	
Series	Code	Alkyl	R	Log *P*	MIC [μg/mL] (μM)	IC_50_ [μM]	SI
A	1	Butyl	-	0.71	50	n.d.	
A	2	Pentyl	-	1.13	12.5 (64.7)	>1000	>15.5
A	3	Hexyl	-	1.55	6.25 (30.2)	>250^c^	>8.3
A	4	Heptyl	-	1.97	3.13 (14.1)	>50^c^	>3.5
	5	Butyl	-	1.37	>100	n.d.	
	6	Pentyl	-	1.78	>100	n.d.	
	7	Hexyl	-	2.2	>100	n.d.	
	8	Heptyl	-	2.62	25	n.d.	
B	9	Propyl	-	–0.62	>100	n.d.	
B	10	Butyl	-	–0.2	>100	n.d.	
B	11	Pentyl	-	0.22	50	n.d.	
B	12	Hexyl	-	0.64	>100	n.d.	
B	13	Heptyl	-	1.05	>100	n.d.	
C	14	Butyl	H	2.61	25	n.d.	
C	15	Pentyl	H	3.03	25	n.d.	
C	16	Hexyl	H	3.45	6.25 (22.1)	>50^c^	>2.3
C	17	Heptyl	H	3.87	6.25 (21.0)	>25^c^	>1.2
C	18	Butyl	2-Cl	3.17	100	n.d.	
C	19	Pentyl	2-Cl	3.59	>100	>100^c^	
C	20	Hexyl	2-Cl	4.01	12.5 (39.3)	313.6	8
C	21	Heptyl	2-Cl	4.42	6.25 (18.8)	>50^c^	>2.7
C	22	Butyl	3-CF_3_	3.53	25	n.d.	
C	23	Pentyl	3-CF_3_	3.95	3.13 (9.3)	238.3	25.7
C	24	Hexyl	3-CF_3_	4.37	12.5 (35.6)	>100^c^	>2.8
C	25	Heptyl	3-CF_3_	4.79	6.25 (17.1)	>100^c^	>5.8
C	26	Butyl	4-CH_3_	3.1	25	n.d.	
C	27	Pentyl	4-CH_3_	3.52	6.25 (22.1)	>100^c^	>4.5
C	28	Heptyl	4-CH_3_	4.35	25	n.d.	
C	29	Butyl	4-OH	2.22	100	n.d.	
C	30	Pentyl	4-OH	2.64	6.25 (21.9)	>100^c^	>4.6
C	31	Hexyl	4-OH	3.06	6.25 (20.9)	>25^c^	>1.2
C	32	Heptyl	4-OH	3.48	3.13 (10.0)	>50^c^	>5.0
D	33	Propyl	-	1.28	>100	n.d.	
D	34	Butyl	-	1.7	>100	n.d.	
D	35	Pentyl	-	2.12	>100	n.d.	
D	36	Hexyl	-	2.54	>100	n.d.	
D	37	Heptyl	-	2.95	>100	n.d.	
	PZA	-	-	–1.31	>100^b^	n.d.	
	INH	-	-	–0.64	0.2	n.d.	

a PZA, pyrazinamide; INH, isoniazid; n.d., not determined. Selectivity index (SI) = IC_50_ [μM]/MIC [μM] calculated for active compounds having MIC < 25 μg/mL.

b The MIC value from testing at pH = 5.6 (acidic) is 6.25–12.5 μg/mL. The value stated in the table is from testing at pH = 6.6 (neutral).

c Measurements at higher concentrations were not possible due to the precipitation of the tested compound in the cell culture medium.

Regarding antimycobacterial activity against *Mtb* H37Rv, the main observation is the superior activity of derivatives with direct alkyl chain attachment (series A: **1**–**4** and series C: **14**–**32**) over the alkanamido derivatives (series B and D), which were all inactive (mostly MIC >100 μg/mL). The inactivity might be explained, especially in compounds with unsubstituted carboxamide at C2, by the generally lowered lipophilicity (as seen in the predicted log *P*) of the alkanamido derivatives in comparison to alkyl derivatives. For example, the highest homologue from group B, compound **13** (log *P* = 1.05), barely leveled the lipophilicity of the alkyl derivative **2** (log *P* = 1.13), which was the first homologue to have significant activity. Another explanation might relate to the possibility of hydrolysis (chemical or enzymatic) of the amidic bond in the carboxamido linker, resulting in only weakly active 5-aminopyrazinoic acid (MIC = 0.8 mM).^30^ For one and only compound **1**, the antimycobacterial activity had been published previously, i.e., MIC = 25 μg/mL against *Mtb* H37Rv and MIC > 200 μg/mL against both *M. avium* and *M. kansasii*,^31,32^ which is in accordance with our findings, i.e., MIC = 50 μg/mL against *Mtb* H37Rv and MIC > 100 μg/mL against both *M. avium* and *M. kansasii*.

5-Alkylpyrazinoic acids (**5**–**8**) derived from group A carboxamides were inactive. The MIC = 25 μg/mL (which is the activity threshold set in this paper) of the highest homologue **8** might be attributed to nonspecific effects, possibly connected with the expected surface activity.

For series A and C, the activity is positively correlated with the length of the alkyl chain, with butyl being the least favorable, as seen in compounds **1** (MIC = 50 μg/mL) from series A, and **14** (R = H; MIC = 25 μg/mL), **18** (R = 2-Cl; MIC = 100 μg/mL), **22** (R = 3-CF_3_; MIC = 25 μg/mL), **26** (R = 4-CH_3_; MIC = 25 μg/mL), and **29** (R = 4-OH; MIC = 100 μg/mL) from series C. Besides compounds with the butyl side chain, other compounds of series A and C were active. For series A, the optimum log *P* value appears to be higher than 1.1, while in the C series, the activity is observed in derivatives with log *P* values above 3.5. In series C, the activity was present in derivatives with both electron-donating and electron-withdrawing substituents, and both lipophilic and hydrophilic substituents on the benzene ring. The most active compounds inhibiting the growth of *Mtb* H37Rv at MIC = 3.13–6.25 μg/mL showed biological activity comparable to that of the standard PZA (MIC = 6.25–12.5 μg/mL) at acidic pH. However, the compounds did not reach the activity of the other standard isoniazid (MIC = 0.2 μg/mL).

For the purpose of demonstrating the selectivity of the most active compounds, the selectivity index (SI) was calculated as the ratio of micromolar IC_50_ toward the cell line HepG2 to micromolar MIC against *Mtb* H37Rv. In the case of two compounds, the SI was higher than 15 (SI > 15.5 for compound **2** and SI = 25.7 for compound **23**), which is beneficial from the point of potential drug safety and especially low hepatotoxicity.

None of the title compounds exerted significant antimycobacterial activity (MIC ≤ 25 μg/mL) against any of the other tested (nontuberculous) mycobacterial strains, except for compounds **11**, **12**, **34–36** that were active against *M. kansasii* (MIC = 6.25 μg/mL), compound **23** was active against *M. kansasii* (MIC = 12.5 μg/mL), and compound **24** was active against *M. smegmatis* (MIC = 3.91 μg/mL). The full results of these screenings are shown in Table S4 in the Supporting Information.

Compounds **4** (HpPZA) and **24** (Hx-3-CF_3_-anilide) were selected as representatives of active compounds from their structural groups and tested for the inhibition of MDR clinical isolates of *Mtb*. Compound **4** was selected as the most active derivative in its structural group, while the selection of anilide **24** was motivated by its broad-spectrum antimycobacterial activity (see Supporting Information, Table S4). The MDR strains were resistant to streptomycin, isoniazid, rifampicin, and pyrazinamide; see Supporting Information Table S1 for the susceptibility profiles. Both compounds showed potent activity against MDR clinical isolates of *Mtb,* with compound **4** overshadowing compound **24,** showing excellent activity with MIC ≤1.56 μg/mL comparable to the first-line anti-TB drug ethambutol. The activity was preserved despite resistance to other first-line anti-TB drugs, pointing to a different mechanism of action or drug target. For results, see Table 2 and Supporting Information Figure S14.

**Table 2:** Inhibitory Activities of Compounds **4** and **24** against Virulent Reference Strain *Mtb* H37Rv and MDR Clinical Isolates of *Mtb* Expressed as Minimum Inhibitory Concentration (MIC in μg/mL)^a^

Strain	4 (HpPZA)	24 (Hx-3-CF_3_-anilide)	INH	CIP	EMB
***Mtb H37Rv***	≤1.56	12.5	0.39	0.2	0.39
***Mtb IZAK***	6.25	25	12.5 R	0.2	1.56
***Mtb MATI***	≤1.56	6.25	12.5 R	0.1	1.56

a INH, isoniazid; CIP, ciprofloxacin; EMB, ethambutol; R,resistant.

### 2.3 *In Vitro* Antibacterial and Antifungal Activity

As a complementary test, final compounds of series C were screened for their antibacterial and antifungal activity. The panel involved four Gram-positive and four Gram-negative bacterial strains and eight fungal strains of clinical significance. None of the tested compounds showed antibacterial or antifungal activity up to the highest tested concentration governed by compounds’ solubility (500 μM for most compounds). For detailed results, see Table S5 and Table S6 in Supporting Information.

### 2.4 Comparison to Previous Work

Results in Table 3 provide a direct comparison of the antimycobacterial activity against *Mtb* H37Rv of the title derivatives with unsubstituted carboxamide at C2 (alkyl derivatives of series A and alkanamido derivatives of series B) and the 5-alkylamino derivatives previously published by our group.^19^ 5-Alkylpyrazine-2-carboxamides (series A) reported in our current study were of superior activity compared to the other structures at all alkyl chain lengths. Direct attachment of the alkyl substituent was favorable, while inserting an amidic or amino spacer led to a decrease in the activity.

**Table 3:**
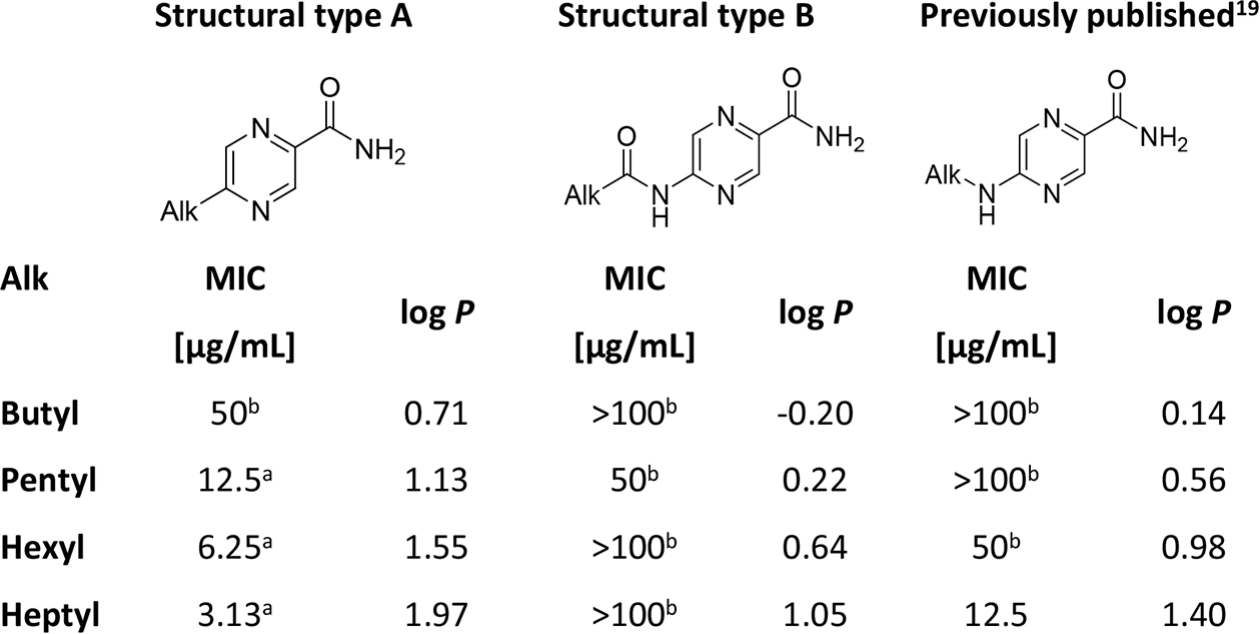
Comparison of Antimycobacterial Activity against *Mtb* H37Rv of Simple 5-Substituted Pyrazine-2-carboxamides

a Indicates the best activity for a given length of the alkyl chain.

b Denotes inactive compounds (defined as MIC >25 μg/mL). Log *P* was calculated with ChemDraw 22.2.0.

In an analogical comparison for *N*-phenylpyrazine-2-carboxamides (Table 4), we noticed the significant activity of previously published 5-alkylamino derivatives,^22^ the preserved activity of 5-alkyl derivatives (structural type C), and again the lack of activity of 5-alkanamido compounds (structural type D).

**Table 4:**
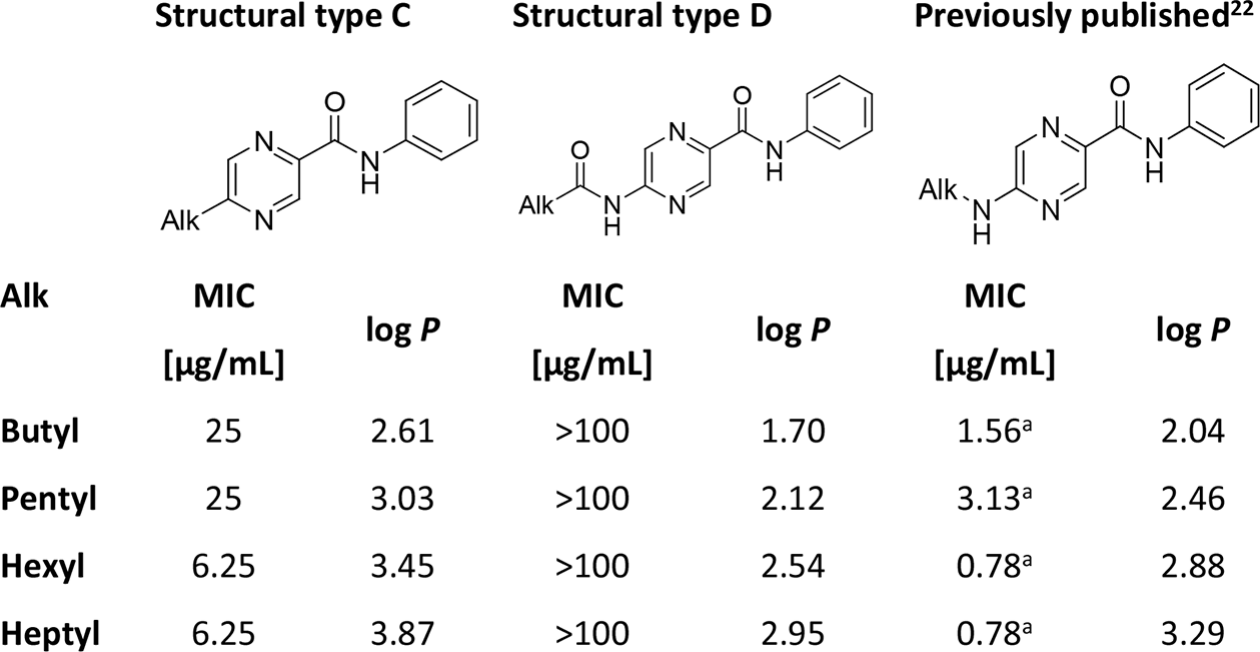
Comparison of Antimycobacterial Activity against *Mtb* H37Rv of 5-Substituted *N-*Phenylpyrazine-2-carboxamides

a Indicates the best activity for a given length of the alkyl chain. Log *P* was calculated with ChemDraw 22.2.0.

### 2.5 Investigation of the Mechanism of Action

#### 2.5.1 Inhibition of Enoyl-ACP Reductase (InhA)

Selected 5-alkyl-*N*-phenylpyrazine-2-carboxamides (structural type C) were evaluated for their potential to inhibit mycobacterial enoyl-[acyl-carrier-protein] reductase (enoyl-ACP reductase, InhA, UniProt P9WGR1). InhA belongs to the Fatty Acid Synthase II (FAS II) pathway and is responsible for the synthesis of mycolic acids. InhA is a clinically validated antimycobacterial target and is the primary target of the first-line anti-TB drug isoniazid (INH). The rationale for the testing is the presence of the InhA inhibitor pharmacophore^33^ (Figure 2) in our title compounds. The pharmacophore is visualized on two confirmed diphenyl ether class inhibitors, triclosan (TCL) and its alkylated derivative PT70 ^34^ (N.B. the corresponding position of the terminal alkyl relative to the linker).

**Figure 2:**
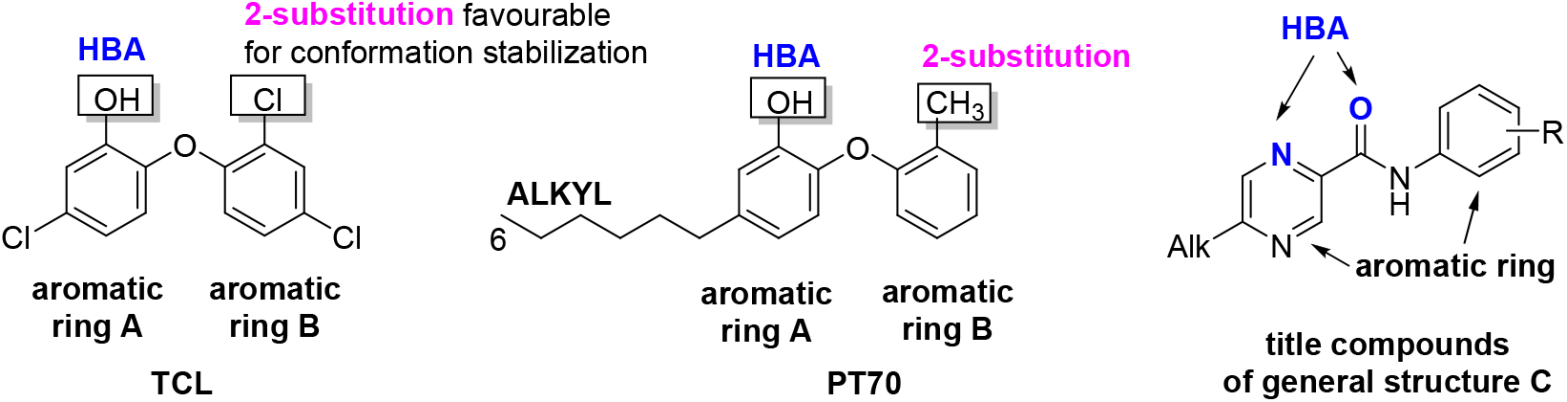
Enoyl-ACP-reductase inhibitor pharmacophore,^33^ depicted on confirmed inhibitors triclosan (TCL) and PT70, projected on the title compounds of series C.

Preliminary docking studies (see Supporting Information, Figures S16–S17) indeed predicted that our title compounds could bind to the active site of InhA in a manner similar to that of the confirmed inhibitors. The carbonyl oxygen of the amidic linker acted as an HBA (accepting an H-bond from Tyr158 and an H-bond from 2′–OH of the ribose of the NAD+ cofactor), and the alkyl chain was oriented into the entry tunnel, which, in a normal situation, is occupied by the alkyl chain of the growing fatty acid intermediate. Nevertheless, in the enzymatic assay,^35^ all tested compounds failed to inhibit InhA up to the highest tested concentration of 100 μM. Therefore, InhA is not the molecular target for the title compounds. For results, see Table S7 in the Supporting Information.

#### 2.5.2 Enzymatic Hydrolysis *via* Mycobacterial Pyrazinamidase Mtb-PncA

The first-line anti-TB drug pyrazinamide (PZA) is a multitarget inhibitor, but the most significant targets/mechanisms of action, including the inhibition of mycobacterial aspartate decarboxylase PanD,^12,13^ require the conversion of the prodrug PZA to active pyrazinoic acid (POA). The activation happens through the action of mycobacterial pyrazinamidase (PncA).^11^ To test whether the same activation pathway is possible for the title 5-alkylcarboxamides of series A, selected compounds (BuPZA, PePZA, and PePOA) were incubated with PncA from *M. tuberculosis* (Mtb-PncA, UniProt I6XD65). The hydrolysis product 5-alkylpyrazinoic acid was detected *via* the formation of the colored Fe^2+^-complex both visually and spectrophotometrically at λ = 458 nm (modified Wayne assay^36^). The extent of hydrolysis was compared to that of the natural substrate PZA. The Mtb-PncA enzyme worked correctly and hydrolyzed PZA to POA, but neither of the two tested 5-alkylcarboxamides (BuPZA and PePZA) was significantly hydrolyzed in comparison to PZA (Figure 3).

**Figure 3:**
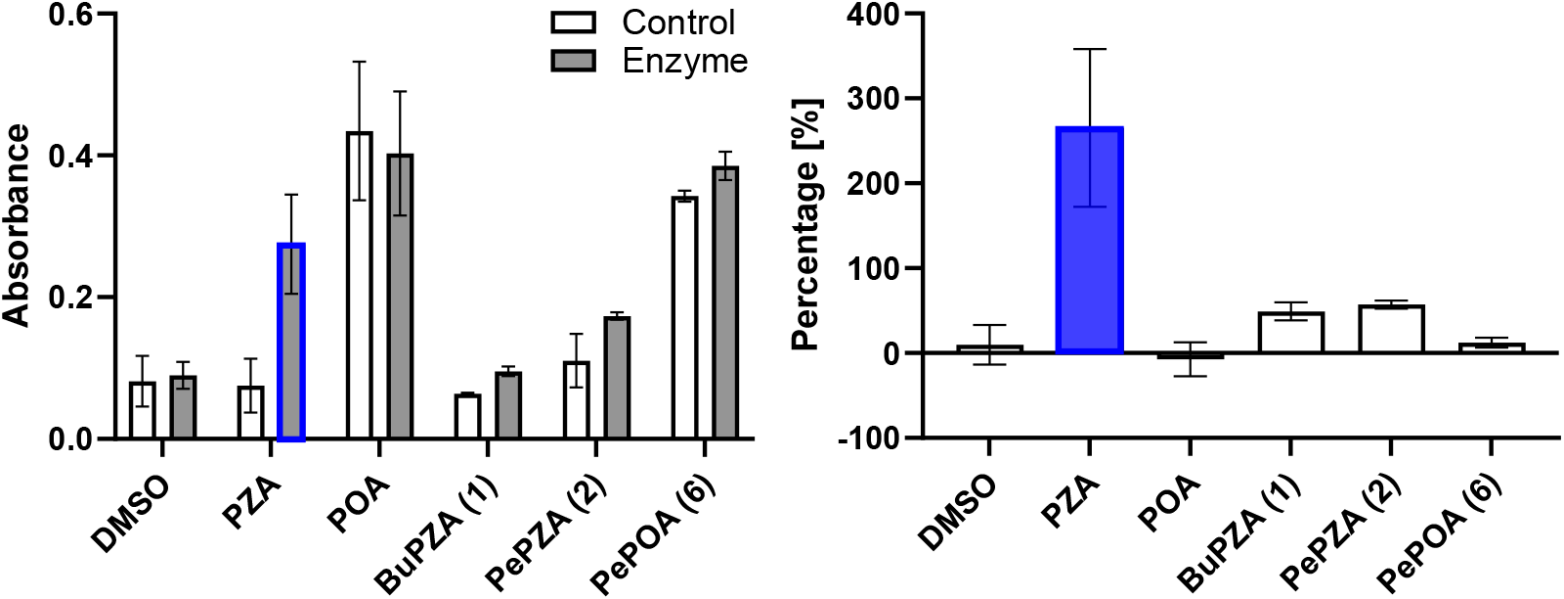
Hydrolysis by pyrazinamidase (PncA). The absorbance corresponds to the concentration of the hydrolytic product—carboxylic acid. Error bars represent 95% CI. Blue denotes significant hydrolysis. Left: Comparison of measured absorbance after incubation without (control) and with Mtb-PncA. POA and PePOA are included as controls to verify the formation and detection of the complex. Right: Normalized percentage increase of absorbance of the sample after incubation with Mtb-PncA.

To further experimentally support our findings, we attempted to test the activity of Mtb-PncA preincubated with the corresponding 5-alkylpyrazinecarbonitriles (1:100). Our rationale was that if the compounds could correctly enter the active site of Mtb-PncA, we should observe lower activity of the enzyme due to irreversible, covalent inhibition as previously described for unsubstituted pyrazine-2-carbonitrile.^37^ No inhibition of enzyme activity was detected (Figure 4). This means that the 5-alkylcarbonitriles, and by extension also the 5-alkylcarboxamides discussed above, were probably unable to correctly interact within the active site of Mtb-PncA.

**Figure 4:**
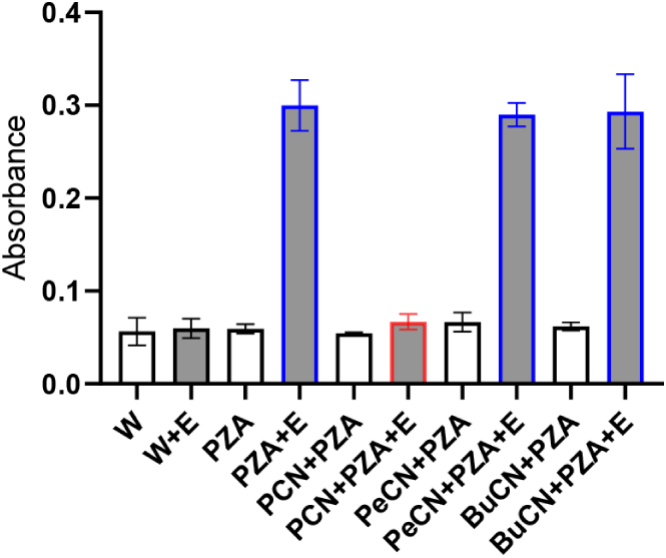
Inhibition of pyrazinamidase (PncA) with pyrazine-2-carbonitriles. Error bars represent 95% CI. W—water; E—enzyme (PncA); PCN—pyrazine-2-carbonitrile. Grayed columns—with enzyme; white columns—controls w/o enzyme. Blue—functional enzyme, significant hydrolysis. Red—enzyme inhibited.

Reasons for insufficient hydrolysis of 5-substituted PZA derivatives by Mtb-PncA were rationalized also computationally by analyzing the available crystallographic structure of Mtb-PncA (PDB ID: 3PL1), to which we modeled the product of the enzymatic hydrolysis, POA, from the complex of a related nicotinamidase from *Acinetobacter baumannii* (PDB ID: 2WTA). We found that the 5-substitution of the pyrazine core is incompatible with the small size and shape of the binding cavity of Mtb-PncA as exemplified in Figure 5. The pyrazine ring is buried deep inside the Mtb-PncA active site pocket, and the H5 hydrogen is facing toward the wall of the protein (specifically toward Trp68) and is thus unable to accommodate any larger substituent.

**Figure 5:**
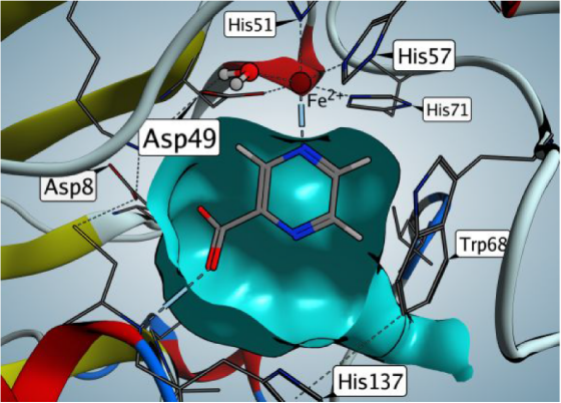
Active site of mycobacterial pyrazinamidase Mtb-PncA, PDB ID: 3PL1 (pyrazinoic acid as the hydrolytic product was modeled based on PDB ID: 2WTA).

#### 2.5.3 Final Considerations on the Mechanism of Action

The molecular mechanism of action of the active compounds remains to be determined. In the group of 5-alkylpyrazine-2-carboxamides (series A), we proved that the compounds are not hydrolyzed by Mtb-PncA. We cannot exclude hydrolysis by other less specific means. Still, if we take it as a premise that the carboxamides act in their nonhydrolyzed form, then the mechanism of action could be similar to 5-chloropyrazine-2-carboxamide (5-Cl-PZA), that is, inhibition of the fatty acid synthesis by interfering with Fatty Acid Synthase I (FAS I, UniProt P95029) complex.^9,10^ Unfortunately, the interaction of 5-Cl-PZA with FAS I was never described at the molecular level.

The activity in the 5-alkylpyrazinoic acids was observed only in the highest (heptyl) homologue **8**. In this case, the long alkyl chain might enhance penetration through the mycobacterial cell wall, increasing the effectiveness of the nonspecific mechanism of action based on proton shuttling and acidification of the cytoplasm, consistent with the theory of the nonspecific mechanism of action of PZA.^6^

In the series of binuclear 5-alkyl-*N*-phenylpyrazine-2-carboxamides (series C), we did not confirm InhA as the target. There are several publications on antimycobacterial *N*-phenylpyrazine-2-carboxamides,^20,22,23,28,38,39^ but none of them proved a specific target, except for the work of Bouz et al., in which the specifically substituted derivatives bearing the 4-aminosalicylic acid fragment inhibited the folate pathway of mycobacteria.^40^ However, this is not applicable to our compounds in series C, which had activity with various substituents on the benzene ring.

## 3 Conclusions

In this work, we have prepared 37 simple derivatives of pyrazinamide with 5-alkyl (series A and C) or 5-alkanamido (series B and D) substitution on the pyrazine ring. The series contained both derivatives with unsubstituted carboxamidic groups (series A and B), *N*-phenyl-substituted carboxamide (series C and D), and free carboxylic moiety (5-alkylpyrazinoic acids). Generally, elongating the 5-alkyl chain increased the *in vitro* antimycobacterial activity, whereas the 5-alkanamido-substituted compounds were inactive. In series C (*N*-phenylpyrazine-2-carboxamides), different simple substituents were tolerated on the benzene ring (both electron-donating and electron-withdrawing, both lipophilic and hydrophilic), and the length of the alkyl chain was the main determinant of the antimycobacterial activity. Compounds **4** (HpPZA) and **24** (Hx-3-CF_3_-anilide), selected as active representatives of their structural types, retained their antimycobacterial activity against MDR clinical isolates of *M. tuberculosis*.

Selected 5-alkyl-*N*-phenylpyrazine-2-carboxamides (series C) were tested for the inhibition of mycobacterial enoyl-ACP reductase (InhA). Despite their structural similarity to known inhibitors of the diphenyl ether type (triclosan and its alkyl derivatives) and the positive results of molecular docking to InhA, the title compounds showed no significant activity. As the last step, we tested whether the 5-alkylpyrazine-2-carboxamides (series A) could be the substrates of mycobacterial pyrazinamidase Mtb-PncA. We found that our compounds are not hydrolyzed by Mtb-PncA and concluded that the 5-alkyl substituent is incompatible with the small catalytic site of Mtb-PncA. This was also confirmed by the inability to inhibit Mtb-PncA with 5-alkylpyrazine-2-carbonitriles, while unsubstituted pyrazine-carbonitrile is a known covalent inhibitor of Mtb-PncA. Although 5-substitution was incompatible with proper binding to Mtb-PncA, analysis of the Mtb-PncA:POA complex suggested that 6-substitution might be tolerated in the case of minor conformational adjustments in the catalytic site.

To sum up, this study brought several promising 5-alkyl derivatives of the first-line anti-TB drug pyrazinamide. To our knowledge, for the first time, we addressed the issue of whether simple pyrazinamide derivatives can be substrates of Mtb-PncA, which is the enzyme responsible for the activation of pyrazinamide.

## 4 Materials and Methods

### 4.1 General Information

All chemicals, reagents, and solvents were of reagent or higher grade purity and were purchased from Merck (Darmstadt, Germany) unless stated otherwise. Solvents for flash chromatography (hexane and ethyl acetate) were purchased from Penta (Prague, Czech Republic). The progress of reactions was checked using Merck Silica 60 F254 TLC plates (Merck) with UV detection at 254 nm wavelength. Microwave-assisted reactions were performed in a CEM Discover microwave reactor with a focused field (CEM Corporation, Matthews, NC, USA) connected to an Explorer 24 autosampler (CEM Corporation). The instrument was running under CEM’s Synergy software to set and monitor the conditions of reactions. An internal infrared sensor monitored the bulk temperature of the reaction mixture. All obtained products were purified with a preparative flash chromatograph CombiFlash Rf (Teledyne Isco Inc., Lincoln, NE, USA). Gradient elution was performed by using a mixture of hexane and ethyl acetate as the mobile phase. Silica gel (0.040–0.063 mm, Merck) was used as the stationary phase.

NMR spectra were recorded using a Varian VNMR S500 (Varian, Palo Alto, CA, USA) at 500 MHz for ^1^H and 125 MHz for ^13^C. Chemical shifts were reported in ppm and were referred indirectly to tetramethylsilane *via* the signal of the solvent (2.49 for ^1^H and 39.7 for ^13^C in DMSO-*d*_6_; 7.26 for ^1^H and 77.0 for ^13^C in CDCl_3_). Infrared spectra were recorded with a spectrometer FT-IR Nicolet 6700 (Thermo Scientific, Waltham, MA, USA) using attenuated total reflectance (ATR) methodology on a germanium crystal. Elemental analysis was measured with a Vario MICRO cube Element Analyzer (Elementar Analysensysteme, Hanau, Germany). All values regarding elemental analyses are given as percentages. Melting points were determined in open capillaries on a Stuart SMP30 melting point apparatus (Bibby Scientific Limited, Staffordshire, UK) and are uncorrected. Yields are expressed as percentages of theoretical yields and refer to the isolated products (chromatographically pure) after all purification steps.

The theoretical lipophilicity parameter log *P* was calculated with ChemDraw 22.2.0 (64-bit version, PerkinElmer Informatics, USA). Molecular modeling was done in MOE 2022.09 (Chemical Computing Group, Montreal, Canada) under the AMBER10:EHT force field.

### 4.2 Chemistry

Full characterization and purity checks of the synthesized compounds can be found in Supporting Information, Section 2.

#### 4.2.1 5-Alkylpyrazine-2-carbonitriles (BuCN, PeCN, HxCN, HpCN)

A 250 mL beaker was charged with a solution of pyrazine-2-carbonitrile (5.0 g, 0.048 mol) in water (150 mL), heated to 80 °C, and silver nitrate (0.82 g, 0.005 mol, 0.1 equiv), and the corresponding carboxylic acid (0.048 mol, 1 equiv) were added. Ammonium peroxydisulfate (12.1 g, 0.053 mol, 1.1 equiv) in water (40 mL) was then added dropwise while stirring, and the temperature was maintained at 75–80 °C for 1 h. After cooling, the mixture was extracted with EtOAc (3 × 100 mL). The combined organic layers were dried over anhydrous sodium sulfate; the solvents were evaporated under reduced pressure, and residue was subjected to flash chromatography on silica, using gradient elution with 0–25% EtOAc in hexane.

#### 4.2.2 5-Alkylpyrazine-2-carboxamides (**1–4**)

In a 250 mL beaker, a mixture of concentrated hydrogen peroxide (30% v/v water solution, 10 equiv, 20 mL) and distilled water (140 mL) was stirred at 50 °C, and its pH was adjusted to pH = 9 using a 10% (w/w) aqueous solution of sodium hydroxide. The corresponding 5-alkylpyrazine-2-carbonitrile (0.02 mol) was added dropwise. The mixture was heated to 55 °C and stirred for 2 h with continuous pH control, maintaining pH = 9 by intermittently adding several drops of 10% NaOH solution. During the reaction, the carboxamide product usually precipitated as a white solid. After the reaction, the mixture was cooled in an ice bath and the precipitate was filtered. The crude product was washed with a small amount of hexane to get rid of the unreacted carbonitrile (liquid), dried, and submitted to flash chromatography on silica, using gradient elution with 0–50% EtOAc in hexane.

#### 4.2.3 5-Alkylpyrazine-2-carboxylic Acids (**5–8**)

In a 250 mL beaker, 10 mmol of the corresponding 5-alkylpyrazine-2-carboxamide (**1**–**4**) was mixed with 80 mL of 10% (m/m) aqueous solution of sodium hydroxide. The mixture was stirred and heated to 50 °C for 1 h. After the reaction, the still-warm reaction mixture was acidified to pH = 3 by diluted hydrochloric acid (10% v/v). The final acids precipitated as solids (**6**–**8**) were filtered and washed with cold water to yield products of sufficient purity. The butyl-substituted acid (**5**) formed as a viscous, water-immiscible liquid, which was extracted using EtOAc. The combined organic layers were dried over anhydrous sodium sulfate and evaporated to yield the final product **5** as a viscous liquid, which solidified after prolonged standing. All acids **5**–**8** were low-melting solids (mp <70 °C).

#### 4.2.4 5-Alkanamidopyrazine-2-carboxamides (**9–13** and **33–37**)

The 5-amino intermediates were prepared in bulk following the previously reported procedures, that is 5-aminopyrazine-2-carboxamide (5-NH_2_-PZA, **III**)^19^ and 5-amino-*N*-phenyl-pyrazine-2-carboxamide (5-NH_2_-POA-anilide, **IV**).^22^ Final 5-alkanamidopyrazine-2-carboxamides (**9**–**13**: R = H, **33**–**37**: R = Ph) were prepared by the acylation of the corresponding 5-aminopyrazine-2-carboxamide **III** or **IV** with aliphatic acyl chlorides as follows. The corresponding 5-aminopyrazine-2-carboxamide **III** or **IV** (1.5 mmol) was placed in a round-bottom flask (50 mL), dissolved in 30 mL of dichloromethane (DCM), and 237 mg of pyridine (3 mmol, 2 equiv) was added. The flask was covered with parafilm and placed in an ice bath. In a separate flask, the corresponding alkanoyl chloride (1.8 mmol, 1.2 equiv) was diluted with 10 mL of DCM. This solution was added dropwise into the main flask stirring in the ice bath, covered with parafilm, and stirred for 2 h. Then the flask was removed from the ice bath and stirred for an additional 2 h at room temperature. After the reaction, the reaction mixture was adsorbed on silica under reduced pressure and subjected to flash chromatography on silica, using gradient elution with 0–50% EtOAc in hexane.

#### 4.2.5 Anilides of 5-Alkylpyrazine-2-carboxylic Acids (**14–32**)

The corresponding 5-alkylpyrazine-2-carboxylic acid (1 mmol) was mixed with 1,1′-carbonyldiimidazole (CDI) in a round-bottom flask (305 mg, 1.9 mmol, 1.9 equiv) in dimethyl sulfoxide (DMSO, 1 mL). The flask with the reactants was covered with foil and left to stir for 15–20 min. During the reaction course, CO_2_ bubbles were released, and once the effervescence subsided, 1 mmol (1 equiv) of the corresponding substituted aniline was added. The mixture was then stirred for 12 h at room temperature with a TLC check (mobile phase: hexane + EtOAc 2:1 (v/v)). The contents of the flask were diluted with distilled water (10 mL), and the product precipitated as a solid. The product was then extracted into EtOAc (3 × 20 mL), and the combined organic phases were dried over anhydrous sodium sulfate, adsorbed on silica gel under reduced pressure, and subjected to flash chromatography (silica, gradient elution 0–20% EtOAc in hexane).

### 4.3 *In Silico* Studies

*In silico* studies were performed in Molecular Operating Environment (MOE) 2022.09 (Chemical Computing Group Inc., Montreal, QC, Canada) under the Amber10:EHT force field. The standard docking protocol as implemented in the software was used. For the full description of the methodology used, see Supporting Information, Section 1.6.

### 4.4 Biology

#### 4.4.1 In Vitro Antimycobacterial Activity

The microdilution broth method based on the Microplate Alamar Blue Assay (MABA) was used. The initial antimycobacterial assay was performed with fast-growing *Mycolicibacterium smegmatis* DSM 43465 (ATCC 607) and *Mycolicibacterium Aurum* DSM 43999 (ATCC 23366), obtained from the German Collection of Microorganisms and Cell Cultures (Braunschweig, Germany). Subsequent assays were performed with the reference strains *Mycobacterium avium* subsp. *avium* Chester CNCTC My 80/72 (ATCC 15769), *Mycobacterium kansasii* Hauduroy CNCTC My 235/80 (ATCC 12478), and *Mycobacterium tuberculosis* H37Rv CNCTC My 331/88 (ATCC 27294), obtained from the Czech National Collection of Type Cultures (CNCTC), National Institute of Public Health (Prague, Czech Republic). The Middlebrook 7H9 broth with a declared pH = 6.6 (Merck), enriched with 0.4% glycerol (Merck) and 10% OADC growth supplement (Himedia, Mumbai, India), was used for cultivation. Tested compounds were dissolved in DMSO and diluted with broth (the final concentration of DMSO did not exceed 2.5% (v/v) and did not affect the growth of mycobacteria). Standards used for activity determination were isoniazid (INH), rifampicin (RIF), and ciprofloxacin (CIP) (Merck). Bacterial viability was assessed after the addition of Alamar Blue (resazurin sodium salt 0.01%), with growth determined by visual inspection of the blue-to-pink color change. The activity was expressed as minimum inhibitory concentration (MIC) in μg/mL. Multidrug-resistant isolates of *M. tuberculosis* (MDR *Mtb*) were obtained from the Department of Clinical Microbiology, University Hospital Hradec Králové, Hradec Králové, Czech Republic, and tested by the same procedure. For a full description of the methodology used, see Supporting Information, Section 1.1.

#### 4.4.2 *In Vitro* Antibacterial Activity

Antibacterial activity evaluation was performed by using a microdilution broth method. Antibacterial evaluation was performed against five reference bacterial strains from the Czech Collection of Microorganisms (CCM, Brno, Czech Republic) (*Staphylococcus aureus* subsp. *aureus* CCM 4223 (ATCC 29213), methicillin-resistant *Staphylococcus aureus* subsp. *aureus* CCM 4750 (ATCC 43300), *Enterococcus faecalis* CCM 4224 (ATCC 29212), *Escherichia coli* CCM 3954 (ATCC 25922), *Pseudomonas aeruginosa* CCM 3955 (ATCC 27853)), and three clinical isolate strains kindly provided from the Department of Clinical Microbiology, University Hospital and Faculty of Medicine in Hradec Králové, Charles University, Czech Republic (*Staphylococcus epidermidis* lab. id. 112-2016, *Klebsiella pneumoniae* lab. id. 64-2016, *Serratia marcescens* lab. id. 62-2016). Antibacterial activity of the tested compounds was expressed as minimum inhibitory concentration (MIC in μM) after 24 and 48 h of static incubation in a dark and humidified atmosphere at 35 ± 2 °C. Visual inspection was used for MIC endpoint evaluation. The internal quality standards of gentamicin and ciprofloxacin (both from Merck) were involved in the assays. For a full description of the methodology used, see Supporting Information, Section 1.2.

#### 4.4.3 *In Vitro* Antifungal Activity

Antifungal activity evaluation was performed using a microdilution broth method. Eight fungal strains (four yeasts and four molds) were used for antifungal activity screening, namely: *Candida albicans* CCM 8320 (ATCC 24433), *Candida krusei* CCM 8271 (ATCC 6258), *Candida parapsilosis* CCM 8260 (ATCC 22019), *Candida tropicalis* CCM 8264 (ATCC 750), *Aspergillus fumigatus* ATCC 204305, *Aspergillus flavus* CCM 8363, *Lichtheimia corymbifera* CCM 8077, and *Trichophyton interdigitale* CCM 8377 (ATCC 9533). Tested strains were purchased from the Czech Collection of Microorganisms (CCM, Brno, Czech Republic) or the American Type Culture Collection (ATCC, Manassas, VA, USA). Visual inspection was used for MIC endpoint evaluation. The internal quality standards amphotericin B (Merck) and voriconazole (Toronto Research Chemicals, CA) were involved in the assays. For a full description of the methodology used, see Supporting Information, Section 1.3.

#### 4.4.4 *In Vitro* Cytotoxicity

The human hepatocellular liver carcinoma cell line HepG2 purchased from Health Protection Agency Culture Collections (ECACC, Salisbury, UK) was cultured in EMEM (Minimum Essential Medium Eagle) (Sigma-Aldrich via Merck, Darmstadt, Germany) supplemented with 10% fetal bovine serum (Sigma-Aldrich), 1% l-glutamine solution (Sigma-Aldrich), and nonessential amino acid solution (Sigma-Aldrich) in a humidified atmosphere containing 5% CO_2_ at 37 °C. The cytotoxicity of the tested compounds was investigated spectrophotometrically at 490 nm (TECAN, Infinite M200, Austria) using the CellTiter 96 AQueous One Solution Cell Proliferation Assay kit (CellTiter 96, PROMEGA, Fitchburg, WI, USA). A standard toxicological parameter IC_50_ was calculated with nonlinear regression from a semilogarithmic plot of incubation concentration versus the percentage of absorbance relative to untreated controls using GraphPad Prism 10.1 software (GraphPad Software, San Diego, CA, USA). For a full description of the methodology used, see Supporting Information, Section 1.4.

### 4.5 Investigation of the Mechanism of Action

#### 4.5.1 Preparation of Pyrazinamidase from *M. tuberculosis* (Mtb-PncA)

The coding sequence of *M. tuberculosis* pyrazinamidase (Mtb-PncA, Uniprot ID: I6XD65), gene *pncA* (Rv2043c), GenBank accession NC_000962.3, coordinates 2 288 681–2 289 241 (− strand), was amplified from genomic DNA isolated from *M. tuberculosis* H37Rv by polymerase chain reaction (PCR). The reaction was performed by using Q5 DNA polymerase (New England Biolabs, Ipswich, MA, USA) with the forward primer 5′-gcgaacagattggtggtggaATGCGGGCGTTGATCATCGTC-3′ and the reverse primer 5′-ttgttagcagaagcttattaGGAGCTGCAAACCAACTCGAC-3′, where the capital letters represent the annealing sequence, while the lowercase letters represent the adaptor sequence complementary to the plasmid. An additional GGA (underlined lowercase in the forward primer) corresponding to the glycine residue was introduced in front of the start codon to facilitate the proteolytic removal of the SUMO tag during protein purification. After separation *via* agarose gel electrophoresis and gel extraction, the purified *Mtb*-PncA PCR product was cloned into a linearized pETRUK vector, encoding an N-terminal charge-modified SUMO fusion tag, by In Fusion (Takara, Kusatsu, Japan. The sequence was validated by Sanger sequencing (LGC Genomics, Germany).

The recombinant construct was transformed into *Escherichia coli* Rosetta 2 (DE3) pLysS and overexpressed by using ZYP-5052 autoinduction media at 18 °C. The bacterial cells were harvested by centrifugation for 10 min at 4 °C at 6000 × *g*. After removing the medium, the cell pellets were frozen and stored at −80 °C. For cell lysis, the frozen pellets were resuspended in lysis buffer containing 20 mM Hepes-NaOH with pH = 7, NaCl 200 mM, and β-mercaptoethanol (β-ME) 5 mM supplemented with 5 mM MgCl_2_ and cold-active cryonase (Takara, Kusatsu, Japan). The resuspended cell pellet was lysed on ice by repeating three times a 5 min sonication step at an amplitude of 70%, waiting 30 min between each cycle. The lysate was clarified by centrifugation at 18,000 × *g* for 45 min at 4 °C. The supernatant was applied onto a 5 mL SP HP column (Cytiva, Marlborough, USA) pre-equilibrated with cation exchange buffer A (20 mM Hepes-NaOH with pH = 7, NaCl 200 mM, β-ME 5 mM). The protein was eluted using a linear gradient of cation exchange buffer B (20 mM Hepes-NaOH pH 7, NaCl 1000 mM, β-ME 5 mM) over 30 column volumes. After examination by SDS-PAGE, the fractions corresponding to the tagged proteins were combined. To remove the SUMO tag, SUMO hydrolase was added (1:1000 mass ratio) to the protein, and the cleavage reaction was then transferred to dialysis tubing and dialyzed overnight at 4 °C in 1 L of buffer (20 mM Bis-tris with pH = 6, 30 mM NaCl, 5 mM β-ME, 10% (v/v) glycerol). The salt and small molecular weight impurities were further removed by an additional dialysis step in fresh dialysis buffer for another 4 h. Then the protein was loaded onto a 5 mL SP HP column (Cytiva, Marlborough, USA) to remove the SUMO tag and SUMO hydrolase, and the flow-through, containing the *Mtb-*PncA, was applied directly onto a Q HP column (Cytiva, Marlborough, USA) and eluted using a linear gradient of dialysis buffer containing an additional 1000 mM NaCl. Pooled fractions were further purified by size exclusion chromatography in a column equilibrated with 10 mM Tris-HCl pH 7, 100 mM NaCl, and 5 mM β-ME. Fractions containing the protein were then concentrated to 20 mg/mL. Aliquots were then flash frozen in liquid nitrogen and stored at −80 °C.

#### 4.5.2 Enzymatic Hydrolysis with Mycobacterial Pyrazinamidase (Mtb-PncA)

Enzymatic hydrolysis by mycobacterial pyrazinamidase (Mtb-PncA) was tested as previously described with minor adjustments.^41^ The method is based on the formation of colored Fe^2+^ complexes of pyrazine-2-carboxylic acid as the product of enzymatic hydrolysis. In a 96-well microtiter plate, 10 μM Mtb-PncA in 50 mM PBS with pH = 6.5 was mixed with 2 mM of tested compounds dissolved in DMSO (or DMSO as a control). The total volume in the well was 100 μL, and the final concentration of DMSO during incubation was 2%. The plate was incubated at room temperature for 15 min followed by the addition of 10 μL of freshly prepared 20% (NH_4_)_2_Fe­(SO_4_)_2_ (Merck) solution in Milli-Q water. The plate was manually shaken and immediately measured. Pyrazinamide (PZA) was used as a positive control. The results were additionally confirmed visually by observing a dark orange-brown coloration.

For the carbonitrile inhibition assay, 50 μM Mtb-PncA in 50 mM PBS with pH = 6.5 was preincubated on ice with 5 mM of the respective pyrazine-2-carbonitrile in Milli-Q water for 2–3 min. The molar ratio of Mtb-PncA to carbonitrile was 1:100. In the 96-well microtiter plate, 20 μL of the enzyme-carbonitrile preincubated solution was mixed with 2 μL of 100 mM PZA in Milli-Q water (the substrate) and diluted with 50 mM PBS with pH = 6.5 to a final volume of 100 μL and incubated for 15 min at laboratory temperature. The activity was determined spectrophotometrically with visual confirmation. Pyrazine-2-carbonitrile was used as a positive control (known as an inhibitor of Mtb-PncA).

All enzymatic assays were measured on a Spark Multimode Microplate Reader (Tecan Austria GmbH, Grödig, Austria) spectrophotometer at 458 nm (maximum absorbance of the POA–Fe^2+^ complex). Experiments were run in quadruplicates, and the mean and 95% confidence intervals (CI) were reported. All data processing was done using GraphPad Prism 10.1 (GraphPad Software, LLC).

## Supplementary Material



## Notes

Materials and Methods used for antimicrobial screening against mycobacteria, bacteria and fungi, cytotoxicity screening, InhA enzymatic assay, and *in silico* modeling. Analytical section, representative ^1^H NMR and ^13^C NMR, HPLC-HRMS chromatograms, and SDS-PAGE analysis of Mtb-PncA. Additional results of antimycobacterial, antibacterial and antifungal screening, and cytotoxicity screening. Results of docking to InhA and InhA enzymatic assays (PDF)
