# Supplementary material for: Targeting Mycobacterium tuberculosis: The Role of Alkyl Substitution in Pyrazinamide Derivatives
Source: ACS Omega. 2026 Jan 14;11(3):3937–48. doi: 10.1021/acsomega.5c07249 (PMC12854518; doi:10.1021/acsomega.5c07249)
Supplement: Supplementary file 1 [file ao5c07249_si_001.pdf]

Targeting *Mycobacterium tuberculosis*: The Role of Alkyl Substitution in Pyrazinamide Derivatives

Martin Juhás[1,2,‡,*], Ghada Bouz[1,3,‡], Luping Pang[4,5], Stephen D. Weeks[5,6], Ondřej Janďourek[1], Klára Konečná[1], Pavla Paterová[7], Pavel Bárta[1], Martina Halířová[1], Marta Kučerová-Chlupáčová[1], Martin Doležal[1], and Jan Zitko[1,*]

[1] Faculty of Pharmacy in Hradec Králové, Charles University, Ak. Heyrovského 1203, 500 03 Hradec Králové, Czech Republic
[2] Faculty of Science, University of Hradec Králové, Rokitanského 62, 500 03 Hradec Králové, Czech Republic
[3] Faculty of Pharmacy, University Business Academy, Heroja Pinkija 4, 21101 Novi Sad, Serbia
[4] Department of Medical Genetics and Cell Biology, School of Basic Medical Sciences, Zhengzhou University, Zhengzhou, Henan, China
[5] Medicinal Chemistry, Rega Institute for Medical Research, KU Leuven, Herestraat 49—Box 1041, 3000 Leuven, Belgium
[6] Pledge Therapeutics, Gaston Geenslaan 1, 3001 Leuven, Belgium
[7] Department of Clinical Microbiology, University Hospital Hradec Králové, Sokolská 581, 500 05 Hradec Králové, Czech Republic
‡ These authors contributed equally.

SUPPORTING INFORMATION

Contents

1. Materials and methods ... 2
- 1.1. Antimycobacterial screening ... 2
- 1.2. Antibacterial screening ... 4
- 1.3. Antifungal screening ... 5
- 1.4. Cytotoxicity screening ... 6
- 1.5. InhA inhibition assay ... 6
- 1.6. *In silico* modelling ... 6
- 1.7. CAS database search ... 7

2. Characterization and analysis ... 8
- 2.1. Characterization of compounds ... 8
- 2.2. Representative ^{1}H and ^{13}C NMR spectra (selected final compounds) ... 15
- 2.3. HPLC-HRMS analysis ... 22
- 2.4. SDS-PAGE analysis of mycobacterial pyrazinamidase Mtb-PncA ... 28

3. Additional results ... 29
- 3.1. MABA assay on MDR strains of *M. tuberculosis* (photos) ... 29
- 3.2. Antimycobacterial activity against non-tuberculous mycobacteria ... 30
- 3.3. Antibacterial screening of compounds of series C ... 31
- 3.4. Antifungal screening of compounds of series C ... 32
- 3.5. Cytotoxicity to HepG2 cell line – viability dose-response curves ... 33
- 3.6. Molecular docking to mycobacterial enoyl-ACP-reductase (InhA) ... 35
- 3.7. Inhibition of enoyl-ACP reductase (InhA) ... 36

4. References ... 37

1. Materials and methods

1.1. *A*ntimycobacterial screening

M. aurum and M. smegmatis

Performed at the Department of Biological and Medical Sciences, Faculty of Pharmacy, Charles University. The microdilution broth method was based on Microplate Alamar Blue Assay (MABA). The initial antimycobacterial assay was performed with **fast-growing** *Mycolicibacterium smegmatis* DSM 43465 (ATCC 607) and *Mycolicibacterium aurum* DSM 43999 (ATCC 23366) obtained from the German Collection of Microorganisms and Cell Cultures (Braunschweig, Germany). The technique used for activity determination was the microdilution broth panel method using 96-well microtiter plates. The cultivation medium was Middlebrook 7H9 broth (Merck, Darmstadt, Germany) enriched with 0.4% of glycerol (Merck) and 10% of Middlebrook OADC growth supplement (Himedia, Mumbai, India). Mycobacterial strains were cultured on Middlebrook 7H9 agar and suspensions were prepared in Middlebrook 7H9 broth. The final density was adjusted to value 1.0 according to the McFarland scale and diluted in a ratio of 1:20 with broth. The tested compounds were dissolved in DMSO (Merck), and then Middlebrook broth was added to obtain a concentration of 2000 µg/mL. Standards used for activity determination were isoniazid (INH), rifampicin (RIF), and ciprofloxacin (CIP) (Merck). Final concentrations were reached by binary dilution and addition of mycobacterial suspension and were set as 500, 250, 125, 62.5, 31.25, 15.625, 7.81, and 3.91 µg/mL. INH was diluted in the range 500–3.91 µg/mL, RIF final concentrations ranged from 50 to 0.39 µg/mL. CIP was used in final concentrations of 1, 0.5, 0.25, 0.125, 0.0625, 0.0313, 0.0156, and 0.0078 µg/mL. The final concentration of DMSO did not exceed 2.5% (v/v) and did not affect the growth of all strains. Positive (broth, DMSO, and bacteria) and negative (broth and DMSO) growth controls were included. Plates containing slow-growing mycobacteria were sealed with polyester adhesive film, and all plates were incubated in the dark at 37 °C without agitation. The addition of a 0.01% solution of resazurin sodium salt followed after 48 hours of incubation for *M. smegmatis* and 72 hours for *M. aurum*. Microtitration panels were then incubated for 2.5 hours to determine the activity against *M. smegmatis* and 4 hours for *M. aurum*. The activity was expressed as minimum inhibitory concentration (MIC in µg/mL). All experiments were conducted in duplicate.

M. avium, M. kansasii, M. tuberculosis H37Rv

Performed at the Department of Clinical Microbiology, University Hospital Hradec Králové, Czech Republic. The microdilution broth method was based on Microplate Alamar Blue Assay (MABA). The reference strains *M. avium* subsp. *avium* Chester CNCTC My 80/72 (ATCC 15769), *Mycobacterium kansasii* Hauduroy CNCTC My 235/80 (ATCC 12478), and *Mycobacterium tuberculosis* H37Rv CNCTC My 331/88 (ATCC 27294) were obtained from the Czech National Collection of Type Cultures (CNCTC), National Institute of Public Health (Prague, Czech Republic). Middlebrook 7H9 broth of declared pH = 6.6 (Merck) enriched with 0.4% of glycerol (Merck) and 10% of OADC growth supplement (Himedia, Mumbai, India) was used for cultivation.

The tested compounds were dissolved and diluted in DMSO and mixed with broth (25 µL of DMSO solution in 2.475 mL of broth) and placed (100 µL) into microplate wells. Mycobacterial inocula were suspended in isotonic saline solution and the density was adjusted to 0.5–1.0 according to the McFarland scale. These suspensions were diluted by 10^{-1} and used to inoculate the testing wells, adding 100 µL of mycobacterial suspension per well. The final concentrations of the tested compounds in wells were 100, 50, 25, 12.5, 6.25, 3.13 and 1.56 µg/mL. INH was used as a standard (inhibition of growth). Positive control (visible growth) consisted of broth, mycobacterial suspension, and DMSO. A total of 30 µL of Alamar Blue working solution (1:1 mixture of 0.02% resazurin sodium salt (aq. sol.) and 10% Tween 80) were added after five days of incubation. Results were then determined after 24 h of incubation. The MIC (in µg/mL) was determined as the lowest concentration that prevented the blue-to-pink color change. All experiments were conducted in duplicate.

Multidrug-resistant clinical isolates of M. tuberculosis

Multidrug-resistant clinical isolates of *M. tuberculosis* (MDR *Mtb*) were tested according to the procedure in the previous section. Three standards (INH – isoniazid; CIP – ciprofloxacin; EMB – ethambutol) were tested alongside our compounds, each standard in concentration range covering the defined resistance breakpoints. Multidrug-resistant isolates were obtained from the Department of Clinical Microbiology, University Hospital Hradec Králové: *Mtb* laboratory ID IZAK, isolated from a 63-year-old man from bronchial aspirate in 2020; *Mtb* laboratory ID MATI, isolated from a 23-year-old man from sputum in 2021. The susceptibility profile of the clinical isolates (Table S1) was determined according to the methodology and breakpoints as defined by CLSI (Clinical and Laboratory Standards Institute).[1]

Table S1. *Susceptibility profiles for the used MDR Mtb strains*

	**MIC [μM]**	
**Standard**	***Mtb* IZAK**	***Mtb* MATI**
STM	4.0 **R**	>16.0 **R**
INH	4.0 **R**	>8.0 **R**
RIF	>8.0 **R**	>8.0 **R**
EMB	0.5 **S**	0.5 **S**
PZA	>16.0 **R**	>128.0 **R**

Standards: STM – streptomycin; INH – isoniazid; RIF – rifampicin; EMB – ethambutol; PZA – pyrazinamide; **R** – resistant; **S** – susceptible

1.2. Antibacterial screening

The microdilution broth method was performed according to EUCAST (The European Committee on Antimicrobial Susceptibility Testing) instructions [2] with slight modifications. Antibacterial evaluation was performed against five reference bacterial strains from the Czech Collection of Microorganisms (CCM, Brno, Czech Republic) (*Staphylococcus aureus* subsp. *aureus* CCM 4223 (ATCC 29213), methicilin-resistant *Staphylococcus aureus* subsp. *aureus* CCM 4750 (ATCC 43300), *Enterococcus faecalis* CCM 4224 (ATCC 29212), *Escherichia coli* CCM 3954 (ATCC 25922), *Pseudomonas aeruginosa* CCM 3955 (ATCC 27853)) and three clinical isolate strains kindly provided from the Department of Clinical Microbiology, University Hospital and Faculty of Medicine in Hradec Králové, Charles University, Czech Republic (*Staphylococcus epidermidis* lab. id. 112-2016, *Klebsiella pneumoniae* lab. id. 64-2016, *Serratia marcescens* lab. id. 62-2016). The cultivation was done in Cation-Adjusted Mueller-Hinton broth (CAMHB, M-H 2 Broth, Merck, Darmstadt, Germany) at 35 ± 2 °C.

The tested compounds were dissolved in DMSO (Merck) to produce stock solutions. The final concentration of DMSO in the cultivation medium did not exceed 1% (v/v) of the total solution composition and did not affect the growth of bacteria. Positive growth controls consisted of test microbe solely, while negative growth controls consisted of cultivation medium and DMSO. Antibacterial activity of tested compounds was expressed as minimum inhibitory concentration (MIC in µM) after 24 and 48 h of static incubation in the dark and humidified atmosphere, at 35 ± 2 °C. Visual inspection was used for MIC endpoint evaluation. The internal quality standards of gentamicin and ciprofloxacin (both from Merck) were involved in assays. MIC of standards was evaluated after 24 of static incubation in the dark and humidified atmosphere, at 35 ± 2 °C (for MIC of standards, see Table S2).

Table S2. *Susceptibility of selected Gram-positive and Gram-negative bacterial strains to the antibacterial drugs, ciprofloxacin and gentamicin*

**Internal quality control**	**ciprofloxacin (µg/mL)**		**gentamicin (µg/mL)**	
**Bacterial strain**	**MIC (spectrophotometric detection*)**	**MIC (visual detection**)**	**MIC (spectrophotometric detection*)**	**MIC (visual detection**)**
Staphylococcus aureus subsp. *aureus* ATCC 29213, CCM 4223	0.128	0.128–0.256	1	1
Staphylococcus aureus subsp. *aureus* **MRSA**, ATCC 43300, CCM 4750	0.128	0.128	16–32	16–32
Staphylococcus epidermidis, clinical isolate	>1.024	>1.024	>8	>8
Enterococcus faecalis, ATCC 29212, CCM 4224	0.512	0.512	16	8
Escherichia coli, ATCC 25922, CCM 3954	0.008	0.008	1–2	1–2
Klebsiella pneumoniae, clinical isolate	>1.024	>1.024	>8	>8
Serratia marcescens, clinical isolate	0.256	0.256	2	2
Pseudomonas aeruginosa, ATCC 27853, CCM 3955	0.128	0.128	0.5	0.5

Notes: Spectrophotometric detection – results were read with a microplate reader (Synergy HTX, BioTek Instruments, Inc., USA) at wavelength 530 nm, MIC – minimum inhibitory concentration

* The MIC of antibacterial agents was defined as the lowest concentration that inhibits 95% of bacterial growth compared to the drug-free control. Results were read 24 h after incubation without agitation at 35 ± 2 °C in a humidified atmosphere.

** The MIC was determined by the naked eye as the lowest drug concentration well in which no visible growth of microbial agent was detected. Results were read after 24 h incubation without agitation at 35 ± 2 °C in a humidified atmosphere.

1.3. Antifungal screening

Antifungal activity evaluation was performed using a microdilution broth method according to EUCAST instructions [3, 4] with slight modifications. Eight fungal strains (four yeast and four molds) were used for antifungal activity screening, namely: *Candida albicans* CCM 8320 (ATCC 24433), *Candida krusei* CCM 8271 (ATCC 6258), *Candida parapsilosis* CCM 8260 (ATCC 22019), *Candida tropicalis* CCM 8264 (ATCC 750), *Aspergillus fumigatus* ATCC 204305, *Aspergillus flavus* CCM 8363, *Lichtheimia corymbifera* CCM 8077, and *Trichophyton interdigitale* CCM 8377 (ATCC 9533). Tested strains were purchased from the Czech Collection of Microorganisms (CCM, Brno, Czech Republic) or the American Type Culture Collection (ATCC, Manassas, VA, USA).

The tested compounds were dissolved in DMSO and diluted in a two-fold manner with RPMI 1640 medium, glutamine, and 2% glucose, buffered to pH = 7.0 with MOPS (3-morpholinopropane-1-sulfonic acid). The final concentration of DMSO in the testing medium did not exceed 1% (v/v) of the total solution composition. Static incubation was performed in the dark and a humid atmosphere, at 35 ± 2 °C, for 24 and 48 h (72 and 120 h for *Trichophyton interdigitale,* respectively). Positive growth controls consisted of test microbe solely, while negative growth controls consisted of cultivation medium and DMSO. Visual inspections were used for MIC endpoint evaluation. The internal quality standards, amphotericin B (Merck) and voriconazole (Toronto Research Chemicals, CA) were involved in assays (for IC_{50}, IC_{90}, and MIC of standards and incubation time, see Table S3).

Table S3. *Susceptibility of yeast and filamentous fungal strains to the antifungal drugs, amphotericin B and voriconazole*

**Internal quality control**	**amphotericin B (µg/mL)**		**voriconazole (µg/mL)**	
**Yeast/mould strain**	**IC_{90} (spectrophotometric detection*)**	**MIC (visual detection**)**	**IC_{50} (spectrophotometric detection***)**	**MIC (visual detection**)**
Candida albicans CCM 8320 (ATCC 24433)	0.5	0.5	0.03	>16
Candida krusei CCM 8271 (ATCC 6258)	1	1	0.25	0.5
Candida parapsilosis CCM 8260 (ATCC 22019)	0.5	0.5	0.03	8
Candida tropicalis CCM 8264 (ATCC 750)	1	1	0.0625	>16
Aspergillus fumigatus (ATCC 204305)	1	1	0.5	1
Aspergillus flavus CCM 8363	8	8	4	>16
Lichtheimia corymbifera CCM 8077	0.5	0.5	>16	>16
Trichophyton interdigitale CCM 8377 (ATCC 9533)	2	2	>16	>16

Notes: Spectrophotometric detection results were read with a microdilution plate reader (Synergy HTX) at wavelength 530 nm.
* The IC_{90} of amphotericin B is defined as the lowest concentration that results in a 90% inhibition of growth compared to the drug-free control. Results were read after 24 h (yeasts) or 48 h (molds) without agitation at 35 ± 2 °C in a humidified atmosphere.
** The MIC was determined by the naked eye as the lowest drug concentration well in which no visible growth of microbial agent was detected. Results were read after 24 h (yeasts) or 48 h (molds) of incubation without agitation at 35 ± 2 °C in a humidified atmosphere.
*** The IC_{50} of voriconazole is defined as the lowest concentration that results in 50% inhibition of growth compared to the drug-free control. Results were read after 24 h (yeasts) or 48 h (molds) microdilution plates cultivation without agitation at 35 ± 2 °C in a humidified atmosphere.

1.4. Cytotoxicity screening

The human hepatocellular liver carcinoma cell line HepG2 purchased from Health Protection Agency Culture Collections (ECACC, Salisbury, UK) was cultured in EMEM (Minimum Essentials Medium Eagle) (Sigma-Aldrich via Merck, Darmstadt, Germany) supplemented with 10% fetal bovine serum (Sigma-Aldrich), 1% L-glutamine solution (Sigma-Aldrich), and non-essential amino acid solution (Sigma-Aldrich) in a humidified atmosphere containing 5% CO_2 at 37 °C.
For subculturing, the cells were harvested after trypsin/EDTA (Sigma-Aldrich) treatment at 37 °C. For cytotoxicity evaluation, the cells treated with the tested substances were used as experimental groups. Untreated HepG2 cells served as controls. Cells from passages 11 to 13 were used.

The cells were seeded in a density of 10,000 cells per well in a 96-well plate. After 24 hours, the cells were treated with each of the tested compounds dissolved in DMSO. The tested compounds were prepared at different incubation concentrations (1–1000 µM) in triplicates according to their solubility. Concurrently, the controls representing 100% cell viability, 0% cell viability (the cells treated with 10% DMSO), no cell control, and vehiculum controls were prepared in triplicates. After 24 h of incubation in a humidified atmosphere containing 5% CO_2 at 37 °C, the reagent from the kit CellTiter 96 AQueous One Solution Cell Proliferation Assay (CellTiter 96, PROMEGA, Fitchburg, WI, USA) was added. After 2 h of incubation at 37 °C, the absorbance of the samples was recorded at 490 nm (TECAN, Infinite M200, Austria). A standard toxicological parameter IC_{50} was calculated with nonlinear regression from a semilogarithmic plot of incubation concentration versus the percentage of absorbance relative to untreated controls using GraphPad Prism 10.1 software (GraphPad Software, San Diego, CA, USA).

1.5. InhA inhibition assay

Inhibition of mycobacterial enoyl-acyl carrier protein reductase (InhA) was tested as described previously[5]. Triclosan was used as the positive control. The experiment was run in independent triplicates, and the results (the residual activity) were reported as mean and 95% confidence intervals (CI).

1.6. *In silico* modelling

Software
In silico calculations were performed in Molecular Operating Environment (MOE) 2022.09 (Chemical Computing Group Inc., Montreal, QC, Canada) under Amber10:EHT force field.

Molecular docking
The ligands for docking (compounds **15**, **20**, **31**) were generated from SMILES, using the MOE built-in function to predict the dominant protonation state at pH = 7.4. In phenolic derivative **31**, the dominant protomer was the neutral species with the non-deprotonated phenolic group (abundance >99%). 3D coordinates were minimized until RMS gradient 0.01 $kcal.mol^{-1}.Å^{-1}$. 3D coordinates of mycobacterial enoyl-ACP-reductase (InhA) were downloaded from the PDB database (PDB ID: 4R9S). The system was prepared by MOE QuickPrep functionality with default settings, which included corrections of structural errors, the addition of hydrogens, calculation of partial charges, 3D optimization of protonation/tautomeric states and H-bond network (Protonate3D), deletion of water molecules further than 4.5 Å from ligand or protein, and a restrained minimization (to RMS gradient of 0.01 $kcal.mol^{-1}.Å^{-1}$) of ligand and pocket residues within 8 Å from the ligand. Subsequently, all solvent molecules were removed, and the NAD cofactor was defined as a part of the receptor.
The docking was focused on the pocket, which was defined as a set of residues having at least one atom within 4.5 Å from the co-crystalized ligand. Parameters of the MOE docking protocol: Docking stage – Placement: Triangle Matcher; Score: London dG; retain 30 poses. Refinement stage – Rigid receptor; Score: GBVI/WSA dG; retain 5 poses. Ligand conformations – Rotate bonds.

1.7. CAS database search

The search was performed via SciFinder^n on November 19, 2025. The search was performed by querying the database and filtering of the results as follows:

1. Query for pyrazine-2-carboxamide as a substructure. The pyrazine ring was locked from forming additional cycles, the nitrogen atoms of the pyrazine ring and the carbonyl atoms of the carboxamide were locked from being substituted. The resulting structure is therefore pyrazinamide substituted on the carboxamide and/or carbons of the pyrazine ring.

2. The resulting compounds were filtered based on the following criteria:

Number of Components:	1
Molecular Weight:	123 to 600
Isotopes:	Not Containing Isotopes
Metals:	Not Containing Metals
Elements:	Compounds containing other elements than C, H, O, N, S, P and halogens were excluded
Results:	**344,893** results (compound entries)

3. Filter: References: Have References	Results: **169,197** Results (compounds)
4. Filter: Bioactivity Indicator: Anti-infective agents	Results: **7,032** Results (compounds)
5. For resulting compounds, all References were looked up:	Results: **27,872** Results (references)
6. References were searched for keyword ”mycobacterium”:	Results: **2,736** Results (references)
7. From the reference, all compounds were looked up …	
… and filtered according to criteria in point 2:	Results: **1,237** Results (compounds)
8. Filter: Bioactivity Indicator: Anti-infective agents	Results: **962** Results (compounds)

2. Characterization and analysis

2.1. Characterization of compounds

Yields refer to isolated, chromatographically pure compounds and the last single step of the synthesis. The results of the elemental analyses are expressed in percentages. In the NMR interpretations, the pyrazine hydrogens are denoted with numbers and benzene hydrogens with primed (‘) numbers.

5-butylpyrazine-2-carboxamide (**1**). White solid. Yield: 85%. mp 150.3–152.4 °C. ^{1}H NMR (500 MHz, $CDCl_3$) δ 9.27 (d, *J* = 1.5 Hz, 1H, H3), 8.37 (d, *J* = 1.5 Hz, 1H, H6), 7.62 (bs, 1H, $CONH_2$), 6.31 (bs, 1H, $CONH_2$), 2.91–2.85 (m, 2H, CH_2), 1.78–1.69 (m, 2H, CH_2), 1.44–1.33 (m, 2H, CH_2), 0.93 (t, *J* = 7.4 Hz, 3H, CH_3). ^{13}C NMR (126 MHz, $CDCl_3$) δ 166.0, 161.2, 143.6, 142.2, 141.6, 35.3, 31.3, 22.3, 13.7. IR (ATR-Ge, cm^{-1}): 3420 (ν(NH), amide), 2966, 2931, 2862 (ν(CH), alkyl), 1666 (ν(C=O), amide), 1413, 1309, 1036, 810, 738, 727. Anal. calcd. for $C_9H_{13}N_3O$ (MW 179.22): C, 60.32; H, 7.31; N, 23.45. Found: C, 60.08; H, 7.32; N, 23.4. HPLC purity 99.68%. HRMS(ESI$^+$): $[M+H]^+$ calcd. for $C_9H_{14}N_3O^+$ (m/z): 180.11314, found 180.11293. CAS: 74416-51-6

5-pentylpyrazine-2-carboxamide (**2**). White solid. Yield: 89%. mp 152.5–153.6 °C. ^{1}H NMR (500 MHz, $CDCl_3$) δ 9.29 (d, *J* = 1.5 Hz, 1H, H3), 8.39 (d, *J* = 1.5 Hz, 1H, H6), 7.63 (bs, 1H, $CONH_2$), 6.34 (bs, 1H, $CONH_2$), 2.88 (t, *J* = 7.9 Hz, 2H, CH_2), 1.82–1.72 (m, 2H, CH_2), 1.41–1.29 (m, 4H, $(CH_2)_2$), 0.89 (t, *J* = 6.8 Hz, 3H, CH_3). ^{13}C NMR (126 MHz, $CDCl_3$) δ 166.0, 161.2, 143.6, 142.2, 141.6, 35.6, 31.4, 28.9, 22.4, 13.9. IR (ATR-Ge, cm^{-1}): 3423 (ν(NH), amide), 2954, 2929, 2870 (ν(CH), alkyl), 1668 (ν(C=O), amide), 1413, 1326, 1036, 802, 738, 725. Anal. calcd. for $C_{10}H_{15}N_3O$ (MW 193.25): C, 62.15; H, 7.82; N, 21.74. Found: C, 62.08; H, 7.92; N, 21.83. HPLC purity 99.52%. HRMS(ESI$^+$): $[M+H]^+$ calcd. for $C_{10}H_{16}N_3O^+$ (m/z): 194.12879, found 194.12863.

5-hexylpyrazine-2-carboxamide (**3**). White solid. Yield: 92%. mp 149.5–150.5 °C. ^{1}H NMR (500 MHz, $CDCl_3$) δ 9.28 (d, *J* = 1.4 Hz, 1H, H3), 8.38 (d, *J* = 1.5 Hz, 1H, H6), 7.62 (bs, 1H, $CONH_2$), 6.29 (bs, 1H, $CONH_2$), 2.88 (t, *J* = 7.8 Hz, 2H, CH_2), 1.83–1.69 (m, 2H, CH_2), 1.42–1.23 (m, 6H, $(CH_2)_3$), 0.86 (t, *J* = 7.1 Hz, 3H, CH_3). ^{13}C NMR (126 MHz, $CDCl_3$) δ 166.0, 161.3, 143.6, 142.2, 141.6, 35.7, 31.5, 29.2, 28.9, 22.4, 14.0. IR (ATR-Ge, cm^{-1}): 3427 (ν(NH), amide), 2952, 2928, 2857 (ν(CH), alkyl), 1664 (ν(C=O), amide), 1413, 1307, 1038, 808, 749, 720. Anal. calcd. for $C_{11}H_{17}N_3O$ (MW 207.28): C, 63.74; H, 8.27; N, 20.27. Found: C, 63.44; H, 8.35; N, 20.29. CAS: 74416-45-8

5-heptylpyrazine-2-carboxamide (**4**). White solid. Yield: 82%. mp 150.0–151.3 °C. ^{1}H NMR (500 MHz, $CDCl_3$) δ 9.29 (d, *J* = 1.5 Hz, 1H, H3), 8.38 (d, *J* = 1.5 Hz, 1H, H6), 7.62 (bs, 1H, $CONH_2$), 6.21 (bs, 1H, $CONH_2$), 2.91–2.84 (m, 2H, CH_2), 1.81–1.71 (m, 2H, CH_2), 1.44–1.17 (m, 8H, $(CH_2)_4$), 0.86 (t, *J* = 6.9 Hz, 3H, CH_3). ^{13}C NMR (126 MHz, $CDCl_3$) δ 165.9, 161.3, 143.6, 142.2, 141.6, 35.7, 31.6, 29.3, 29.2, 29.0, 22.5, 14.0. IR (ATR-Ge, cm^{-1}): 3424 (ν(NH), amide), 2954, 2922, 2850 (ν(CH), alkyl), 1668 (ν(C=O), amide), 1415, 1328, 1036, 801, 739, 722. Anal. calcd. for $C_{12}H_{19}N_3O$ (MW 221.3): C, 65.13; H, 8.65; N, 18.99. Found: C, 64.72; H, 8.75; N, 18.97.

5-butylpyrazine-2-carboxylic acid (**5**). White solid. Yield: 75%. ^{1}H NMR (500 MHz, DMSO-*d$_6$*) δ 13.55 (bs, 1H, COOH), 9.07 (d, *J* = 1.5 Hz, 1H, H3), 8.68 (d, *J* = 1.5 Hz, 1H, H6), 2.88–2.82 (m, 2H, CH_2), 1.73–1.63 (m, 2H, CH_2), 1.37–1.26 (m, 2H, CH_2), 0.89 (t, *J* = 7.4 Hz, 3H, CH_3). ^{13}C NMR (126 MHz, DMSO-*d$_6$*) δ 165.5, 160.8, 144.9, 144.2, 141.5, 34.5, 30.7, 21.9, 13.9. Anal. calcd. for $C_9H_{12}N_2O_2$ (MW 180.21): C, 59.99; H, 6.71; N, 15.55. Found: C, 60.22; H, 6.78; N, 15.42.

5-pentylpyrazine-2-carboxylic acid (**6**). White solid. Yield: 91%. mp 68.1–69.5 °C. ^{1}H NMR (500 MHz, DMSO-*d$_6$*) δ 13.54 (bs, 1H, COOH), 9.07 (d, *J* = 1.4 Hz, 1H, H3), 8.67 (d, *J* = 1.4 Hz, 1H, H6), 2.83 (t, *J* = 7.7 Hz, 2H, CH_2), 1.73–1.64 (m, 2H, CH_2), 1.33–1.22 (m, 4H, $(CH_2)_2$), 0.83 (t, *J* = 6.9 Hz, 3H, CH_3). ^{13}C NMR (126 MHz, DMSO-*d$_6$*) δ 165.5, 160.8, 144.9, 144.2, 141.5, 34.8, 31.0, 28.3, 22.0, 14.0. IR (ATR-Ge, cm^{-1}): 2952, 2929, 2869 (ν(CH), alkyl), 1731 (ν(C=O), monomer H-bonded, carboxylic acid), 1705 (ν(C=O), dimer H-bonded, carboxylic acid), 1422, 1271, 1039, 810, 774, 735. Anal. calcd. for $C_{10}H_{14}N_2O_2$ (MW 194.23): C, 61.84; H, 7.27; N, 14.42. Found: C, 62.05; H, 7.48; N, 14.11. HPLC purity 99.92%. HRMS(ESI$^+$): $[M+H]^+$ calcd. for $C_{10}H_{15}N_2O_2^+$ (m/z): 195.11280, found 195.11266. CAS: 89967-35-1

5-hexylpyrazine-2-carboxylic acid (**7**). White solid. Yield: 89%. mp 60.9–63.2 °C. ^{1}H NMR (500 MHz, DMSO-d_6) δ 13.51 (bs, 1H, COOH), 9.07 (d, *J* = 1.5 Hz, 1H, H3), 8.67 (d, *J* = 1.5 Hz, 1H, H6), 2.84 (t, *J* = 7.7 Hz, 2H, CH_2), 1.74–1.64 (m, 2H, CH_2), 1.34–1.19 (m, 6H, $(CH_2)_3$), 0.83 (t, *J* = 7.2 Hz, 3H, CH_3). ^{13}C NMR (126 MHz, DMSO-d_6) δ 165.5, 160.8, 144.9, 144.2, 141.5, 34.8, 31.2, 28.6, 28.4, 22.2, 14.1. IR (ATR-Ge, cm^{-1}): 2955, 2921, 2855 (ν(CH), alkyl), 1737 (ν(C=O), monomer H-bonded, carboxylic acid), 1710 (ν(C=O), dimer H-bonded, carboxylic acid), 1434, 1278, 1039, 809, 737, 723. Anal. calcd. for $C_{11}H_{16}N_2O_2$ (MW 208.26): C, 63.44; H, 7.74; N, 13.45. Found: C, 63.21; H, 7.99; N, 13.68.

5-heptylpyrazine-2-carboxylic acid (**8**). White solid. Yield: 85%. mp 60.4–62.1 °C. ^{1}H NMR (500 MHz, DMSO-d_6) δ 9.07 (d, *J* = 1.4 Hz, 1H, H3), 8.67 (d, *J* = 1.4 Hz, 1H, H6), 2.88–2.81 (m, 2H, CH_2), 1.74–1.64 (m, 2H, CH_2), 1.33–1.16 (m, 8H, $(CH_2)_4$), 0.83 (t, *J* = 6.9 Hz, 3H, CH_3). COOH proton not visible. ^{13}C NMR (126 MHz, DMSO-d_6) δ 165.5, 160.9, 144.9, 144.2, 141.5, 34.8, 31.3, 28.7, 28.6, 28.6, 22.2, 14.1. IR (ATR-Ge, cm^{-1}): 2954, 2927, 2852 (ν(CH), alkyl), 1694 (ν(C=O), carboxylic acid), 1465, 1302, 814, 740, 725. Anal. calcd. for $C_{12}H_{18}N_2O_2$ (MW 222.29): C, 64.84; H, 8.16; N, 12.6. Found: C, 65.13; H, 8.4; N, 12.37.

5-butyramidopyrazine-2-carboxamide (**9**). White solid. Yield: 33%. mp 249.6–251.6 °C. ^{1}H NMR (500 MHz, DMSO-d_6) δ 11.05 (s, 1H, NHCO), 9.35 (d, *J* = 1.5 Hz, 1H, H3), 8.90 (d, *J* = 1.5 Hz, 1H, H6), 8.10 (s, 1H, $CONH_2$), 7.67 (s, 1H, $CONH_2$), 2.44 (t, *J* = 7.3 Hz, 2H, CH_2), 1.67–1.56 (m, 2H, CH_2), 0.91 (t, *J* = 7.4 Hz, 3H, CH_3). ^{13}C NMR (126 MHz, DMSO-d_6) δ 172.9, 165.1, 150.6, 142.2, 140.1, 133.9, 37.9, 18.3, 13.7. IR (ATR-Ge, cm^{-1}): 3430, 3300 (ν(NH), amide), 3176 (ν(NH), amide), 2968 (ν(CH), alkyl), 1687 (ν(C=O), amide), 1657 (ν(C=O), amide), 1601, 1535, 1500, 1200, 1025. Anal. calcd. for $C_9H_{12}N_4O_2$ (MW 208.22): C, 51.92; H, 5.81; N, 26.91. Found: C, 52.1; H, 5.73; N, 26.83.

5-pentanamidopyrazine-2-carboxamide (**10**). White solid. Yield: 46%. mp 221.3–224.0 °C. ^{1}H NMR (500 MHz, DMSO-d_6) δ 11.05 (s, 1H, NHCO), 9.34 (d, *J* = 1.5 Hz, 1H, H3), 8.90 (d, *J* = 1.5 Hz, 1H, H6), 8.10 (s, 1H, $CONH_2$), 7.67 (s, 1H, $CONH_2$), 2.46 (t, *J* = 7.4 Hz, 2H, CH_2), 1.64–1.52 (m, 2H, CH_2), 1.37–1.26 (m, 2H, CH_2), 0.88 (t, *J* = 7.4 Hz, 3H, CH_3). ^{13}C NMR (126 MHz, DMSO-d_6) δ 173.0, 165.1, 150.6, 142.2, 140.1, 133.9, 35.7, 26.9, 21.9, 13.9. IR (ATR-Ge, cm^{-1}): 3421, 3337 (ν(NH), amide), 3178 (ν(NH), amide), 2955, 2872 (ν(CH), alkyl), 1691 (ν(C=O), amide), 1636 (ν(C=O), amide), 1592, 1542, 1505, 1200, 1034. Anal. calcd. for $C_{10}H_{14}N_4O_2$ (MW 222.25): C, 54.04; H, 6.35; N, 25.21. Found: C, 54.01; H, 6.24; N, 25.16.

5-hexanamidopyrazine-2-carboxamide (**11**). White solid. Yield: 42%. mp 223.7–225.4 °C. ^{1}H NMR (500 MHz, DMSO-d_6) δ 11.05 (s, 1H, NHCO), 9.35 (d, *J* = 1.5 Hz, 1H, H3), 8.90 (d, *J* = 1.5 Hz, 1H, H6), 8.11 (s, 1H, $CONH_2$), 7.68 (s, 1H, $CONH_2$), 2.45 (t, *J* = 7.4 Hz, 2H, CH_2), 1.64–1.55 (m, 2H, CH_2), 1.28 (m, 4H, $(CH_2)_2$), 0.86 (t, *J* = 6.8 Hz, 3H, CH_3). ^{13}C NMR (126 MHz, DMSO-d_6) δ 173.0, 165.1, 150.6, 142.2, 140.1, 133.9, 36.0, 30.9, 24.5, 22.0, 14.0. IR (ATR-Ge, cm^{-1}): 3437, 3210 (ν(NH), amide), 3176 (ν(NH), amide), 2950, 2871 (ν(CH), alkyl), 1686 (ν(C=O), amide), 1637 (ν(C=O), amide), 1537, 1504, 1471, 1201, 1024. Anal. calcd. for $C_{11}H_{16}N_4O_2$ (MW 236.28): C, 55.92; H, 6.83; N, 23.71. Found: C, 55.98; H, 6.75; N, 23.72.

5-heptanamidopyrazine-2-carboxamide (**12**). White solid. Yield: 58%. mp 218.9–220.0 °C. ^{1}H NMR (500 MHz, DMSO-d_6) δ 11.05 (s, 1H, NHCO), 9.34 (s, 1H, H3), 8.90 (s, 1H, H6), 8.10 (s, 1H, $CONH_2$), 7.67 (s, 1H, $CONH_2$), 2.45 (t, *J* = 7.4 Hz, 2H, CH_2), 1.63–1.53 (m, 2H, CH_2), 1.38–1.16 (m, 6H, $(CH_2)_3$), 0.84 (t, *J* = 6.5 Hz, 3H, CH_3). ^{13}C NMR (126 MHz, DMSO-d_6) δ 173.0, 165.1, 150.6, 142.2, 140.1, 133.9, 36.0, 31.2, 28.4, 24.8, 22.1, 14.1. IR (ATR-Ge, cm^{-1}): 3445, 3322 (ν(NH), amide), 3174 (ν(NH), amide), 2918, 2856 (ν(CH), alkyl), 1684 (ν(C=O), amide), 1640 (ν(C=O), amide), 1538, 1504, 1471, 1201, 1023. Anal. calcd. for $C_{12}H_{18}N_4O_2$ (MW 250.3): C, 57.58; H, 7.25; N, 22.38. Found: C, 57.43; H, 7.12; N, 22.4.

5-octanamidopyrazine-2-carboxamide (**13**). White solid. Yield: 32%. mp 215.6–217.1 °C. ^{1}H NMR (500 MHz, DMSO-d_6) δ 11.05 (s, 1H, NHCO), 9.34 (d, *J* = 1.5 Hz, 1H, H3), 8.90 (d, *J* = 1.5 Hz, 1H, H6), 8.10 (s, 1H, $CONH_2$), 7.67 (s, 1H, $CONH_2$), 2.45 (t, *J* = 7.4 Hz, 2H, CH_2), 1.63–1.54 (m, 2H, CH_2), 1.33–1.18 (m, 8H, $(CH_2)_4$), 0.84 (t, *J* = 6.8 Hz, 3H, CH_3). ^{13}C NMR (126 MHz, DMSO-d_6) δ 173.0, 165.0, 150.6, 142.2, 140.1, 133.9, 36.0, 31.3, 28.7, 28.6, 24.8, 22.2, 14.1. IR (ATR-Ge, cm^{-1}): 3445, 3315 (ν(NH), amide), 3172 (ν(NH), amide), 2927, 2854 (ν(CH), alkyl), 1686, 1639 (ν(C=O), amide), 1539, 1504, 1471, 1201, 1024. Anal. calcd. for $C_{13}H_{20}N_4O_2$ (MW 264.33): C, 59.07; H, 7.63; N, 21.2. Found: C, 58.52; H, 7.53; N, 21.09.

5-butyl-*N*-phenylpyrazine-2-carboxamide (**14**). White solid. Yield: 23%. mp 120.5–120.9 °C. ^{1}H NMR (500 MHz, $CDCl_3$) δ 9.64 (s, 1H, CONH), 9.40 (d, *J* = 1.4 Hz, 1H, H3), 8.43 (d, *J* = 1.4 Hz, 1H, H6), 7.80–7.73 (m, 2H, H2´, H6´), 7.44–7.36 (m, 2H, H3´, H5´), 7.21–7.14 (m, 1H, H4´), 2.93 (t, *J* = 7.6 Hz, 2H, CH_2), 1.84–1.74 (m, 2H, CH_2), 1.49–1.37 (m, 2H, CH_2), 0.98 (t, *J* = 7.4 Hz, 3H, CH_3). ^{13}C NMR (126 MHz, $CDCl_3$) δ 161.3, 161.0, 143.7, 141.8, 137.4, 129.1, 124.6, 119.7, 35.4, 31.4, 22.4, 13.8. IR (ATR-Ge, cm^{-1}): 3445, 3315 (ν(NH), amide), 3172 (ν(NH), amide), 2927, 2854 (ν(CH), alkyl), 1686 (ν(C=O), amide), 1639 (ν(C=O), amide), 1539, 1504, 1471, 1201, 1024. Anal. calcd. for $C_{15}H_{17}N_3O$ (MW 255.32): C, 70.56; H, 6.71; N, 16.46. Found: C, 70.55; H, 6.74; N, 16.3.

5-pentyl-*N*-phenylpyrazine-2-carboxamide (**15**). White solid. Yield: 70%. mp 112.7–113.5 °C. ^{1}H NMR (500 MHz, DMSO-*d*$_6$) δ 10.61 (s, 1H, CONH), 9.18 (d, *J* = 1.4 Hz, 1H, H3), 8.68 (d, *J* = 1.4 Hz, 1H, H6), 7.92–7.84 (m, 2H, H2´, H6´), 7.39–7.30 (m, 2H, H3´, H5´), 7.16–7.07 (m, 1H, H4´), 2.89 (t, *J* = 7.5 Hz, 2H, CH_2), 1.77–1.67 (m, 2H, CH_2), 1.37–1.24 (m, 4H, $(CH_2)_2$), 0.85 (t, *J* = 7.0 Hz, 3H, CH_3). ^{13}C NMR (126 MHz, DMSO-*d*$_6$) δ 161.9, 160.7, 143.3, 142.8, 142.6, 138.4, 128.8, 124.2, 120.6, 34.7, 31.0, 28.4, 22.0, 14.0. IR (ATR-Ge, cm^{-1}): 3346 (ν(NH), amide), 2961, 2932, 2871 (ν(CH), alkyl), 1673 (ν(C=O), amide), 1532, 1445, 1030, 753, 691, 671. Anal. calcd. for $C_{16}H_{19}N_3O$ (MW 269.35): C, 71.35; H, 7.11; N, 15.6. Found: C, 71.84; H, 7.07; N, 15.75.

5-hexyl-*N*-phenylpyrazine-2-carboxamide (**16**). White solid. Yield: 58%. mp 118.6–120.0 °C. ^{1}H NMR (500 MHz, $CDCl_3$) δ 9.64 (bs, 1H, CONH), 9.40 (d, *J* = 1.4 Hz, 1H, H3), 8.43 (d, *J* = 1.5 Hz, 1H, H6), 7.80–7.73 (m, 2H, H2´, H6´), 7.44–7.37 (m, 2H, H3´, H5´), 7.20–7.15 (m, 1H, H4´), 2.92 (t, *J* = 7.9 Hz, 2H, CH_2), 1.84–1.75 (m, 2H, CH_2), 1.45–1.27 (m, 6H, $(CH_2)_3$), 0.90 (t, *J* = 7.0 Hz, 3H, CH_3). ^{13}C NMR (126 MHz, $CDCl_3$) δ 161.3, 161.0, 143.7, 141.8, 137.4, 129.1, 124.6, 119.7, 35.7, 31.5, 29.3, 28.9, 22.5, 14.0. IR (ATR-Ge, cm^{-1}): 3344 (ν(NH), amide), 2948, 2932, 2854 (ν(CH), alkyl), 1670 (ν(C=O), amide), 1523, 1443, 1157, 1036, 752, 691, 677. Anal. calcd. for $C_{17}H_{21}N_3O$ (MW 283.38): C, 72.06; H, 7.47; N, 14.83. Found: C, 72.32; H, 7.45; N, 14.85.

5-heptyl-*N*-phenylpyrazine-2-carboxamide (**17**). White solid. Yield: 36%. mp 98.0–100.2 °C. ^{1}H NMR (500 MHz, $CDCl_3$) δ 9.64 (bs, 1H, CONH), 9.40 (d, *J* = 1.5 Hz, 1H, H3), 8.42 (d, *J* = 1.5 Hz, 1H, H6), 7.80–7.73 (m, 2H, H2´, H6´), 7.43–7.35 (m, 2H, H3´, H5´), 7.20–7.13 (m, 1H, H4´), 2.92 (t, *J* = 7.8 Hz, 2H, CH_2), 1.84–1.75 (m, 2H, CH_2), 1.45–1.23 (m, 8H, $(CH_2)_4$), 0.89 (t, *J* = 6.9 Hz, 3H, CH_3). ^{13}C NMR (126 MHz, $CDCl_3$) δ 161.3, 161.0, 143.7, 141.8, 137.4, 129.1, 124.6, 119.7, 35.7, 31.7, 29.3, 29.2, 29.0, 22.6, 14.0. IR (ATR-Ge, cm^{-1}): 3346 (ν(NH), amide), 2957, 2933, 2850 (ν(CH), alkyl), 1670 (ν(C=O), amide), 1522, 1443, 1155, 1035, 752, 691, 676. Anal. calcd. for $C_{18}H_{23}N_3O$ (MW 297.4): C, 72.7; H, 7.8; N, 14.13. Found: C, 72.75; H, 7.83; N, 14.08.

5-butyl-*N*-(2-chlorophenyl)pyrazine-2-carboxamide (**18**). White solid. Yield: 18%. mp 74.2–74.8 °C. ^{1}H NMR (500 MHz, $CDCl_3$) δ 10.33 (bs, 1H, CONH), 9.40 (d, *J* = 1.4 Hz, 1H, H6), 8.63 (dd, *J* = 1.6, 8.3 Hz, 1H, H6´), 8.49 (d, *J* = 1.4 Hz, 1H, H3), 7.44 (dd, *J* = 1.5, 8.1 Hz, 1H, H3´), 7.38–7.32 (m, 1H, H5´), 7.14–7.07 (m, 1H , H4´), 2.94 (t, *J* = 7.7 Hz, 2H, CH_2), 1.84–1.75 (m, 2H, CH_2), 1.49–1.38 (m, 2H, CH_2), 0.98 (t, *J* = 7.4 Hz, 3H, CH_3). ^{13}C NMR (126 MHz, $CDCl_3$) δ 161.6, 161.2, 143.7, 142.2, 141.8, 134.4, 129.2, 127.8, 124.9, 123.4, 121.2, 35.4, 31.4, 22.4, 13.8. IR (ATR-Ge, cm^{-1}): 3336 (ν(NH), amide), 2963, 2932, 2872 (ν(CH), alkyl), 1693 (ν(C=O), amide), 1593, 1533, 1442, 1306, 1032, 755, 677. Anal. calcd. for $C_{15}H_{16}ClN_3O$ (MW 289.76): C, 62.18; H, 5.57; N, 14.5. Found: C, 61.95; H, 5.37; N, 14.1.

***N*-(2-chlorophenyl)-5-pentylpyrazine-2-carboxamide** (**19**). White solid. Yield: 16%. mp 80.1–80.2 °C. ^{1}H NMR (500 MHz, $CDCl_3$) δ 10.33 (s, 1H, CONH), 9.39 (d, *J* = 1.5 Hz, 1H, H3), 8.64 (dd, *J* = 1.5, 8.2 Hz, 1H, H6´), 8.48 (d, *J* = 1.5 Hz, 1H, H6), 7.43 (dd, *J* = 1.5, 8.0 Hz, 1H, H3´), 7.38–7.32 (m, 1H, H5´), 7.13–7.07 (m, 1H, H4´), 2.93 (t, *J* = 7.7 Hz, 2H, CH_2), 1.86–1.76 (m, 2H, CH_2), 1.43–1.34 (m, 4H, $(CH_2)_2$), 0.92 (t, *J* = 6.9 Hz, 3H, CH_3). ^{13}C NMR (126 MHz, $CDCl_3$) δ 161.5, 161.2, 143.7, 142.1, 141.8, 134.4, 129.2, 127.8, 124.9, 123.4, 121.1, 35.7, 31.4, 28.9, 22.4, 13.9. IR (ATR-Ge, cm^{-1}): 3336 (ν(NH), amide), 2963, 2933, 2857 (ν(CH), alkyl), 1693 (ν(C=O), amide), 1593, 1527, 1442, 1308, 1032, 755, 677. Anal. calcd. for $C_{16}H_{18}ClN_3O$ (MW 303.79): C, 63.26; H, 5.97; N, 13.83. Found: C, 63.68; H, 6.05; N, 13.54.

***N*-(2-chlorophenyl)-5-hexylpyrazine-2-carboxamide** (**20**). White solid. Yield: 57%. mp 84.3–84.5 °C. ^{1}H NMR (500 MHz, $CDCl_3$) δ 10.33 (bs, 1H, CONH), 9.39 (d, *J* = 1.4 Hz, 1H, H3), 8.63 (dd, *J* = 1.5, 8.3 Hz, 1H, H6′), 8.48 (d, *J* = 1.5 Hz, 1H, H6), 7.43 (dd, *J* = 1.5, 8.0 Hz, 1H, H3′), 7.37–7.32 (m, 1H, H5′), 7.12–7.07 (m, 1H, H4′), 2.93 (t, *J* = 7.7 Hz, 2H, CH_2), 1.84–1.76 (m, 2H, CH_2), 1.44–1.27 (m, 6H, $(CH_2)_3$), 0.90 (t, *J* = 7.1 Hz, 3H, CH_3). ^{13}C NMR (126 MHz, $CDCl_3$) δ 161.5, 161.2, 143.7, 142.1, 141.8, 134.4, 129.2, 127.8, 124.9, 123.4, 121.1, 35.7, 31.5, 29.2, 28.9, 22.5, 14.0. IR (ATR-Ge, cm^{-1}): 3338 (ν(NH), amide), 2952, 2931, 2854 (ν(CH), alkyl), 1693 (ν(C=O), amide), 1594, 1536, 1442, 1307, 1032, 754, 676. Anal. calcd. for $C_{17}H_{20}ClN_3O$ (MW 317.82): C, 64.25; H, 6.34; N, 13.22. Found: C, 64.66; H, 6.39; N, 13.01.

***N*-(2-chlorophenyl)-5-heptylpyrazine-2-carboxamide** (**21**). White solid. Yield: 5%. mp 86.9–87.8 °C. ^{1}H NMR (500 MHz, $CDCl_3$) δ 10.34 (bs, 1H, CONH), 9.40 (d, *J* = 1.4 Hz, 1H, H3), 8.64 (dd, *J* = 1.6, 8.3 Hz, 1H, H6′), 8.49 (d, *J* = 1.4 Hz, 1H, H6), 7.44 (dd, *J* = 1.5, 8.0 Hz, 1H, H3′), 7.38–7.33 (m, 1H, H5′), 7.13–7.08 (m, 1H, H4′), 2.94 (t, *J* = 7.7 Hz, 2H, CH_2), 1.85–1.76 (m, 2H, CH_2), 1.45–1.22 (m, 8H, $(CH_2)_4$), 0.89 (t, *J* = 6.9 Hz, 3H, CH_3). ^{13}C NMR (126 MHz, $CDCl_3$) δ 161.6, 161.2, 143.7, 142.2, 141.8, 134.4, 129.2, 127.8, 124.9, 123.4, 121.2, 35.8, 31.7, 29.3, 29.2, 29.0, 22.6, 14.0. IR (ATR-Ge, cm^{-1}): 3336 (ν(NH), amide), 2953, 2930, 2854 (ν(CH), alkyl), 1693 (ν(C=O), amide), 1594, 1533, 1442, 1307, 1032, 755, 678. Anal. calcd. for $C_{18}H_{22}ClN_3O$ (MW 331.84): C, 65.15; H, 6.68; N, 12.66. Found: C, 65.31; H, 6.45; N, 12.31.

5-butyl-*N*-[3-(trifluoromethyl)phenyl]pyrazine-2-carboxamide (**22**). White solid. Yield: 20%. mp 67.9–68.3 °C. ^{1}H NMR (500 MHz, $CDCl_3$) δ 9.77 (bs, 1H, CONH), 9.40 (d, *J* = 1.4 Hz, 1H, H3), 8.44 (d, *J* = 1.4 Hz, 1H, H6), 8.06 (bs, 1H, H2′), 8.00–7.94 (m, 1H, H4′), 7.51 (t, *J* = 8.0 Hz, 1H, H5′), 7.42 (d, *J* = 7.8 Hz, 1H, H6′), 2.94 (t, *J* = 7.7 Hz, 2H, CH_2), 1.84–1.74 (m, 2H, CH_2), 1.49–1.37 (m, 2H, CH_2), 0.98 (t, *J* = 7.4 Hz, 3H, CH_3). ^{13}C NMR (126 MHz, $CDCl_3$) δ 161.8, 161.3, 143.8, 141.9, 141.3, 137.9, 131.55 (q, *J* = 32.6 Hz), 129.7, 123.79 (q, *J* = 274.6 Hz), 122.71, 121.11 (q, *J* = 3.9 Hz), 116.45 (q, *J* = 4.0 Hz), 35.4, 31.3, 22.4, 13.8. IR (ATR-Ge, cm^{-1}): 3360 (ν(NH), amide), 2958, 2929, 2872 (ν(CH), alkyl), 1688 (ν(C=O), amide), 1539, 1448, 1338, 1321, 1167, 1122, 1071, 1033, 901, 790, 697, 660. Anal. calcd. for $C_{16}H_{16}F_3N_3O$ (MW 323.32): C, 59.44; H, 4.99; N, 13.00 Found: C, 59.87; H, 4.92; N, 12.88.

5-pentyl-*N*-[3-(trifluoromethyl)phenyl]pyrazine-2-carboxamide (**23**). White solid. Yield: 20%. mp 70.1–70.9 °C. ^{1}H NMR (500 MHz, $CDCl_3$) δ 9.77 (s, 1H, CONH), 9.40 (d, *J* = 1.3 Hz, 1H, H3), 8.43 (d, *J* = 1.3 Hz, 1H, H6), 8.06 (bs, 1H, H2′), 7.97 (d, *J* = 8.1 Hz, 1H, H4′), 7.51 (t, *J* = 7.9 Hz, 1H, H5′), 7.42 (d, *J* = 7.8 Hz, 1H, H6′), 2.93 (t, *J* = 7.6 Hz, 2H, CH_2), 1.86–1.76 (m, 2H, CH_2), 1.43–1.33 (m, 4H, $(CH_2)_2$), 0.92 (t, *J* = 6.9 Hz, 3H, CH_3). ^{13}C NMR (126 MHz, $CDCl_3$) δ 161.8, 161.3, 143.8, 141.9, 141.3, 137.9, 131.55 (q, *J* = 32.6 Hz), 129.7, 123.79 (q, *J* = 272.7 Hz), 122.70, 121.10 (q, *J* = 3.8 Hz), 116.45 (q, *J* = 4.0 Hz), 35.7, 31.4, 28.9, 22.4, 13.9. IR (ATR-Ge, cm^{-1}): 3348 (ν(NH), amide), 2955, 2930, 2861 (ν(CH), alkyl), 1688 (ν(C=O), amide), 1545, 1450, 1337, 1322, 1166, 1122, 1070, 1032, 900, 791, 698, 660. Anal. calcd. for $C_{17}H_{18}F_3N_3O$ (MW 337.35): C, 60.53; H, 5.38; N, 12.46. Found: C, 60.42; H, 5.5; N, 12.17.

5-hexyl-*N*-[3-(trifluoromethyl)phenyl]pyrazine-2-carboxamide (**24**). White solid. Yield: 44%. mp 64–64.9 °C. ^{1}H NMR (500 MHz, $CDCl_3$) δ 9.77 (bs, 1H, CONH), 9.40 (d, *J* = 1.4 Hz, 1H, H3), 8.43 (d, *J* = 1.4 Hz, 1H, H6), 8.06 (bs, 1H, H2′), 7.97 (dd, *J* = 2.1, 8.2 Hz, 1H, H4′), 7.51 (t, *J* = 8.0 Hz, 1H, H5′), 7.42 (d, *J* = 7.8 Hz, 1H, H6′), 2.93 (t, *J* = 7.7 Hz, 2H, CH_2), 1.85–1.75 (m, 2H, CH_2), 1.44–1.27 (m, 6H, $(CH_2)_3$), 0.90 (t, *J* = 6.8 Hz, 3H, CH_3). ^{13}C NMR (126 MHz, $CDCl_3$) δ 161.8, 161.3, 143.8, 141.9, 141.3, 137.9, 131.56 (q, *J* = 32.5 Hz), 129.7, 123.8 (q, *J* = 272.3 Hz), 122.71, 121.11 (q, *J* = 3.9 Hz), 116.46 (q, *J* = 3.9 Hz), 35.7, 31.5, 29.2, 28.9, 22.5, 14.0. IR (ATR-Ge, cm^{-1}): 3349 (ν(NH), amide), 2931, 2856 (ν(CH), alkyl), 1689 (ν(C=O), amide), 1544, 1450, 1337, 1322, 1166, 1122, 1070, 1032, 902, 799, 698, 661. Anal. calcd. for $C_{18}H_{20}F_3N_3O$ (MW 351.37): C, 61.53; H, 5.74; N, 11.96. Found: C, 61.71; H, 5.52; N, 12.07.

5-heptyl-*N*-[3-(trifluoromethyl)phenyl]pyrazine-2-carboxamide (**25**). White solid. Yield: 9%. mp 59.1–60.1 °C. ^{1}H NMR (500 MHz, $CDCl_3$) δ 9.77 (bs, 1H, CONH), 9.40 (d, *J* = 1.4 Hz, 1H, H3), 8.43 (d, *J* = 1.4 Hz, 1H, H6), 8.06 (bs, 1H, H2′), 7.97 (dd, *J* = 2.0, 8.3 Hz, 1H, H4′), 7.51 (t, *J* = 7.9 Hz, 1H, H5′), 7.42 (d, *J* = 7.8 Hz, 1H, H6′), 2.93 (t, *J* = 7.8 Hz, 2H, CH_2), 1.84–1.75 (m, 2H, CH_2), 1.44–1.22 (m, 8H, $(CH_2)_4$), 0.89 (t, *J* = 6.9 Hz, 3H, CH_3). ^{13}C NMR (126 MHz, $CDCl_3$) δ 161.8, 161.3, 143.8, 141.9, 141.3, 137.9, 131.55 (q, *J* = 32.4 Hz), 129.7, 123.80 (q, *J* = 272.9 Hz), 122.70, 121.11 (q, *J* = 3.8 Hz), 116.45 (q, *J* = 4.0 Hz), 35.7, 31.7, 29.3, 29.2, 29.0, 22.6, 14.0. IR (ATR-Ge, cm^{-1}): 3357 (ν(NH), amide), 2930, 2858 (ν(CH), alkyl), 1688 (ν(C=O), amide), 1541, 1449, 1337, 1322, 1163, 1122, 1072, 1033, 903, 791, 698, 660. Anal. calcd. for $C_{19}H_{22}F_3N_3O$ (MW 365.4): C, 62.45; H, 6.07; N, 11.5. Found: C, 62.68; H, 5.99; N, 11.21.

5-butyl-*N*-(*p*-tolyl)pyrazine-2-carboxamide (**26**). White solid. Yield: 22%. mp 126.9–127.1 °C. ^{1}H NMR (500 MHz, $CDCl_3$) δ 9.58 (bs, 1H, CONH), 9.39 (d, *J* = 1.4 Hz, 1H, H3), 8.42 (d, *J* = 1.4 Hz, 1H, H6), 7.68–7.61 (m, 2H, AA', BB', H2', H4'), 7.23–7.17 (m, 2H, AA', BB', H3', H5'), 2.93 (t, *J* = 7.7 Hz, 2H, CH_2), 2.36 (s, 3H, Ar-CH_3), 1.83–1.73 (m, 2H, CH_2), 1.48–1.37 (m, 2H, CH_2), 0.97 (t, *J* = 7.4 Hz, 3H, CH_3). ^{13}C NMR (126 MHz, $CDCl_3$) δ 161.1, 160.9, 143.6, 141.9, 141.8, 134.8, 134.3, 129.6, 119.7, 35.4, 31.4, 22.4, 20.9, 13.8. IR (ATR-Ge, cm^{-1}): 3346 (ν(NH), amide), 2954, 2931, 2871 (ν(CH), alkyl), 1670 (ν(C=O), amide), 1518, 1320, 1030, 819, 674. Anal. calcd. for $C_{16}H_{19}N_3O$ (MW 269.35): C, 71.35; H, 7.11; N, 15.6. Found: C, 71.56; H, 7.15; N, 15.55.

5-pentyl-*N*-(*p*-tolyl)pyrazine-2-carboxamide (**27**). White solid. Yield: 68%. mp 115.5–117.7 °C. ^{1}H NMR (500 MHz, DMSO-*d$_6$*) δ 10.54 (s, 1H, CONH), 9.17 (d, *J* = 1.4 Hz, 1H, H3), 8.68 (d, *J* = 1.4 Hz, 1H, H6), 7.79–7.73 (m, 2H, AA', BB', H2', H4'), 7.19–7.13 (m, 2H, AA', BB', H3', H5'), 2.89 (t, *J* = 7.6 Hz, 2H, CH_2), 2.28 (s, 3H, Ar-CH_3), 1.78–1.68 (m, 2H, CH_2), 1.38–1.24 (m, 4H, $(CH_2)_2$), 0.86 (t, *J* = 6.8 Hz, 3H, CH_3). ^{13}C NMR (126 MHz, DMSO-*d$_6$*) δ 161.7, 160.6, 143.2, 142.9, 142.6, 135.9, 133.3, 129.2, 120.6, 34.7, 31.0, 28.4, 22.0, 20.7, 14.0. IR (ATR-Ge, cm^{-1}): 3343 (ν(NH), amide), 2960, 2931, 2854 (ν(CH), alkyl), 1667 (ν(C=O), amide), 1515, 1317, 1157, 1034, 813, 677. Anal. calcd. for $C_{17}H_{21}N_3O$ (MW 283.38): C, 72.06; H, 7.47; N, 14.83. Found: C, 72.31; H, 7.66; N, 14.94.

5-heptyl-*N*-(*p*-tolyl)pyrazine-2-carboxamide (**28**). White solid. Yield: 13%. mp 120.5–121.3 °C. ^{1}H NMR (500 MHz, $CDCl_3$) δ 9.59 (bs, 1H, CONH), 9.40 (d, *J* = 1.5 Hz, 1H, H3), 8.42 (d, *J* = 1.4 Hz, 1H, H6), 7.68–7.61 (m, 2H, AA', BB', H2', H4'), 7.22–7.18 (m, 2H, AA', BB', H3', H5'), 2.92 (t, *J* = 7.7 Hz, 2H, CH_2), 2.36 (s, 3H, Ar-CH_3), 1.85–1.75 (m, 2H, CH_2), 1.46–1.21 (m, 8H, $(CH_2)_4$), 0.89 (t, *J* = 6.9 Hz, 3H, CH_3). ^{13}C NMR (126 MHz, $CDCl_3$) δ 161.2, 160.9, 143.7, 141.9, 141.8, 134.9, 134.3, 129.6, 119.7, 35.7, 31.7, 29.3, 29.2, 29.0, 22.6, 20.9, 14.0. IR (ATR-Ge, cm^{-1}): 3344 (ν(NH), amide), 2930, 2851 (ν(CH), alkyl), 1670 (ν(C=O), amide), 1522, 1156, 1035, 813, 680. Anal. calcd. for $C_{19}H_{25}N_3O$ (MW 311.43): C, 73.28; H, 8.09; N, 13.49. Found: C, 72.98; H, 8.13; N, 13.22.

5-butyl-*N*-(4-hydroxyphenyl)pyrazine-2-carboxamide (**29**). Pale yellow solid. Yield: 31%. mp 154.1–155.5 °C. ^{1}H NMR (500 MHz, DMSO-*d$_6$*) δ 10.40 (s, 1H, CONH), 9.31 (s, 1H, OH), 9.15 (d, *J* = 1.4 Hz, 1H, H3), 8.65 (d, *J* = 1.4 Hz, 1H, H6), 7.69–7.61 (m, 2H, AA', BB', H2', H4'), 6.79–6.71 (m, 2H, AA', BB', H3', H5'), 2.88 (t, *J* = 7.7 Hz, 2H, CH_2), 1.75–1.65 (m, 2H, CH_2), 1.39–1.27 (m, 2H, CH_2), 0.90 (t, *J* = 7.4 Hz, 3H, CH_3). ^{13}C NMR (126 MHz, DMSO-*d$_6$*) δ 161.3, 160.5, 154.2, 143.1, 143.0, 142.6, 130.0, 122.3, 115.2, 34.5, 30.9, 21.9, 13.9. IR (ATR-Ge, cm^{-1}): 3337 (ν(NH), amide), 2957, 2932, 2871 (ν(CH), alkyl), 1661 (ν(C=O), amide), 1520, 1440, 1250, 1227, 1036, 829, 675. Anal. calcd. for $C_{15}H_{17}N_3O_2$ (MW 271.32): C, 66.4; H, 6.32; N, 15.49. Found: C, 66.71; H, 6.27; N, 15.54.

***N*-(4-hydroxyphenyl)-5-pentylpyrazine-2-carboxamide** (**30**). White solid. Yield: 32%. mp 136.5–136.7 °C. ^{1}H NMR (500 MHz, DMSO-*d$_6$*) δ 10.39 (s, 1H, CONH), 9.29 (s, 1H, OH), 9.15 (d, *J* = 1.5 Hz, 1H, H3), 8.65 (d, *J* = 1.4 Hz, 1H, H6), 7.67–7.62 (m, 2H, AA', BB', H2', H4'), 6.77–6.72 (m, 2H, AA', BB', H3', H5'), 2.87 (t, *J* = 7.7 Hz, 2H, CH_2), 1.76–1.67 (m, 2H, CH_2), 1.36–1.24 (m, 4H, $(CH_2)_2$), 0.85 (t, *J* = 7.0 Hz, 3H, CH_3). ^{13}C NMR (126 MHz, DMSO-*d$_6$*) δ 161.3, 160.5, 154.2, 143.1, 143.0, 142.5, 130.0, 122.3, 115.2, 34.7, 31.0, 28.4, 22.0, 14.0. IR (ATR-Ge, cm^{-1}): 3566 (ν(OH)), 3349 (ν(NH), amide), 2957, 2931 (ν(CH), alkyl), 1661 (ν(C=O), amide), 1537, 1242, 1106, 1031, 830, 676. Anal. calcd. for $C_{16}H_{19}N_3O_2$ (MW 285.35): C, 67.35; H, 6.71; N, 14.73. Found: C, 67.79; H, 6.67; N, 14.62.

5-hexyl-*N*-(4-hydroxyphenyl)pyrazine-2-carboxamide (**31**). Yellow solid. Yield: 50%. mp 141–141.5 °C. ^{1}H NMR (500 MHz, DMSO-*d$_6$*) δ 10.40 (s, 1H, CONH), 9.31 (bs, 1H, OH), 9.15 (d, *J* = 1.4 Hz, 1H, H3), 8.66 (d, *J* = 1.4 Hz, 1H, H6), 7.68–7.61 (m, 2H, AA', BB', H2', H4'), 6.78–6.70 (m, 2H, AA', BB', H3', H5'), 2.88 (t, *J* = 7.6 Hz, 2H, CH_2), 1.76–1.66 (m, 2H, CH_2), 1.34–1.22 (m, 6H, $(CH_2)_3$), 0.85 (t, *J* = 7.0 Hz, 3H, CH_3). ^{13}C NMR (126 MHz, DMSO-*d$_6$*) δ 161.3, 160.5, 154.2, 143.1, 143.0, 142.6, 130.0, 122.3, 115.2, 34.8, 31.2, 28.7, 28.4, 22.1, 14.1. IR (ATR-Ge, cm^{-1}): 3369, 3336 (ν(NH), amide), 2958, 2927, 2852 (ν(CH), alkyl), 1657 (ν(C=O), amide), 1520, 1448, 1251, 1030, 834, 684. Anal. calcd. for $C_{17}H_{21}N_3O_2$ (MW 299.37): C, 68.2; H, 7.07; N, 14.04. Found: C, 68.43; H, 7.13; N, 13.91.

5-heptyl-*N*-(4-hydroxyphenyl)pyrazine-2-carboxamide (**32**). Yellow solid. Yield: 21%. mp 139.5–141.0 °C. ^{1}H NMR (500 MHz, $CDCl_3$) δ 9.56 (s, 1H, CONH), 9.38 (d, *J* = 1.4 Hz, 1H, H3), 8.43 (d, *J* = 1.4 Hz, 1H, H6), 7.60–7.53 (m, 2H, AA', BB', H2', H4'), 6.90–6.83 (m, 2H, AA', BB', H3', H5'), 6.38 (bs, 1H, OH), 2.92 (t, *J* = 7.6 Hz, 2H, CH_2), 1.83–1.73 (m, 2H, CH_2), 1.43–1.23 (m, 8H, $(CH_2)_4$), 0.88 (t, *J* = 6.9 Hz, 3H, CH_3). ^{13}C NMR (126 MHz, $CDCl_3$) δ 161.2, 161.0, 153.3, 143.5, 142.0, 141.9, 130.0, 122.0, 115.9, 35.6, 31.7, 29.3, 29.2, 29.0, 22.6, 14.0. IR (ATR-Ge, cm^{-1}): 3367, 3335 (ν(NH), amide), 2928, 2852 (ν(CH), alkyl), 1658 (ν(C=O), amide), 1521, 1447, 1252, 1029, 835, 687. Anal. calcd. for $C_{18}H_{23}N_3O_2$ (MW 313.4): C, 68.98; H, 7.4; N, 13.41. Found: C, 69.35; H, 7.36; N, 13.51.

5-butyramido-*N*-phenylpyrazine-2-carboxamide (**33**). White scaly. Yield: 42%. mp 210.8–212 °C. ^{1}H NMR (500 MHz, DMSO-*d*$_6$) δ 11.15 (s, 1H, NHCO), 10.56 (s, 1H, CONH), 9.45 (d, *J* = 1.4 Hz, 1H, H3), 9.02 (d, *J* = 1.4 Hz, 1H, H6), 7.91–7.85 (m, 2H, H2´, H6´), 7.39–7.31 (m, 2H, H3´, H5´), 7.11 (tt, *J* = 7.3, 1.2 Hz, 1H, H4´), 2.45 (t, *J* = 7.3 Hz, 2H, CH_2), 1.68–1.57 (m, 2H, CH_2), 0.91 (t, *J* = 7.4 Hz, 3H, CH_3). ^{13}C NMR (126 MHz, DMSO-*d*$_6$) δ 173.0, 161.6, 150.8, 142.7, 139.9, 138.5, 133.7, 128.8, 124.1, 120.6, 37.9, 18.2, 13.7. IR (ATR-Ge, cm^{-1}): 3238 (ν(NH), amide), 2963, 2873 (ν(CH), alkyl), 1714 (ν(C=O), amide), 1673 (ν(C=O), amide), 1595, 1533, 1512, 1195, 1014, 692. Anal. calcd. for $C_{15}H_{16}N_4O_2$ (MW 284.32): C, 63.37; H, 5.67; N, 19.71. Found: C, 63.57; H, 5.65; N, 19.87.

5-pentanamido-*N*-phenylpyrazine-2-carboxamide (**34**). White scaly. Yield: 80%. mp 213.5–215 °C. ^{1}H NMR (500 MHz, DMSO-*d*$_6$) δ 11.15 (s, 1H, NHCO), 10.55 (s, 1H, CONH), 9.45 (d, *J* = 1.4 Hz, 1H, H3), 9.01 (d, *J* = 1.4 Hz, 1H, H6), 7.91–7.85 (m, 2H, H2´, H6´), 7.38–7.31 (m, 2H, H3´, H5´), 7.10 (tt, *J* = 7.5, 1.2 Hz, H4´, 1H), 2.47 (t, *J* = 7.4 Hz, 2H, CH_2), 1.63–1.54 (m, 2H, CH_2), 1.37–1.26 (m, 2H, CH_2), 0.89 (t, *J* = 7.4 Hz, 3H, CH_3). ^{13}C NMR (126 MHz, DMSO-*d*$_6$) δ 173.1, 161.6, 150.9, 142.7, 139.9, 138.5, 133.7, 128.8, 124.1, 120.6, 35.7, 26.9, 21.9, 13.9. IR (ATR-Ge, cm^{-1}): 3251 (ν(NH), amide), 2965, 2873 (ν(CH), alkyl), 1714 (ν(C=O), amide), 1674 (ν(C=O), amide), 1596, 1537, 1509, 1195, 1015, 692. Anal. calcd. for $C_{16}H_{18}N_4O_2$ (MW 298.35): C, 64.41; H, 6.08; N, 18.78. Found: C, 64.01; H, 5.87; N, 18.29.

5-hexanamido-*N*-phenylpyrazine-2-carboxamide (**35**). White scaly. Yield: 49%. mp 212.4–213.8 °C. ^{1}H NMR (500 MHz, $CDCl_3$) δ 9.57 (s, 1H, NHCO), 9.55 (d, *J* = 1.4 Hz, 1H, H3), 9.16 (d, *J* = 1.4 Hz, 1H, H6), 8.22 (s, 1H, CONH), 7.80–7.73 (m, 2H, H2´, H6´), 7.44–7.36 (m, 2H, H3´, H5´), 7.17 (tt, *J* = 7.4, 1.1 Hz, 1H, H4´), 2.50 (t, *J* = 7.6 Hz, 2H, CH_2), 1.83–1.73 (m, 2H, CH_2), 1.45–1.35 (m, 4H, $(CH_2)_2$), 0.94 (t, *J* = 7.2 Hz, 3H, CH_3). ^{13}C NMR (126 MHz, $CDCl_3$) δ 171.9, 160.7, 149.9, 142.6, 140.0, 137.4, 133.9, 129.1, 124.6, 119.8, 37.4, 31.3, 24.8, 22.4, 13.9. IR (ATR-Ge, cm^{-1}): 3246 (ν(NH), amide), 2957, 2932 (ν(CH), alkyl), 1716 (ν(C=O), amide), 1674 (ν(C=O), amide), 1596, 1537, 1509, 1015, 693. Anal. calcd. for $C_{17}H_{20}N_4O_2$ (MW 312.37): C, 65.37; H, 6.45; N, 17.94. Found: C, 65.72; H, 6.44; N, 18.09.

5-heptanamido-*N*-phenylpyrazine-2-carboxamide (**36**). White scaly. Yield: 45%. mp 206.5–208.4 °C. ^{1}H NMR (500 MHz, DMSO-*d*$_6$) δ 11.15 (s, 1H, NHCO), 10.56 (s, 1H, CONH), 9.45 (d, *J* = 1.5 Hz, 1H, H3), 9.02 (d, *J* = 1.5 Hz, 1H, H6), 7.92–7.86 (m, 2H, H2´, H6´), 7.39–7.31 (m, 2H, H3´, H5´), 7.11 (tt, *J* = 7.4, 1.2 Hz, 1H, H4´), 2.47 (t, *J* = 7.6 Hz, 2H, CH_2), 1.65–1.54 (m, 2H, CH_2), 1.35–1.21 (m, 6H, $(CH_2)_3$), 0.86 (t, *J* = 7.0 Hz, 3H, CH_3). ^{13}C NMR (126 MHz, DMSO-*d*$_6$) δ 173.1, 161.6, 150.9, 142.7, 139.9, 138.5, 133.7, 128.8, 124.1, 120.6, 36.0, 31.2, 28.4, 24.7, 22.1, 14.1. IR (ATR-Ge, cm^{-1}): 3234 (ν(NH), amide), 2932, 2859 (ν(CH), alkyl), 1716 (ν(C=O), amide), 1674 (ν(C=O), amide), 1597, 1537, 1509, 1195, 1015, 693. Anal. calcd. for $C_{18}H_{22}N_4O_2$ (MW 326.4): C, 66.24; H, 6.79; N, 17.17. Found: C, 65.72; H, 6.72; N, 17.11.

5-octanamido-*N*-phenylpyrazine-2-carboxamide (**37**). White scaly. Yield: 61%. mp 191.7–193.4 °C. ^{1}H NMR (500 MHz, DMSO-*d*$_6$) δ 11.15 (s, 1H, NHCO), 10.56 (s, 1H, CONH), 9.45 (s, 1H, H3), 9.02 (s, 1H, H6), 7.92–7.87 (m, 2H, H2´, H6´), 7.39–7.32 (m, 2H, H3´, H5´), 7.15–7.08 (m, 1H, H4´), 2.47 (t, *J* = 7.5 Hz, 2H, CH_2), 1.65–1.56 (m, 2H, CH_2), 1.35–1.19 (m, 8H, $(CH_2)_4$), 0.85 (t, *J* = 6.6 Hz, 3H, CH_3). ^{13}C NMR (126 MHz, DMSO-*d*$_6$) δ 173.1, 161.6, 150.9, 142.7, 139.9, 138.5, 133.7, 128.8, 124.1, 120.6, 36.0, 31.3, 28.7, 28.6, 24.8, 22.2, 14.1. IR (ATR-Ge, cm^{-1}): 3235 (ν(NH), amide), 2929, 2854 (ν(CH), alkyl), 1716 (ν(C=O), amide), 1673 (ν(C=O), amide), 1596, 1537, 1510, 1195, 1014, 692. Anal. calcd. for $C_{19}H_{24}N_4O_2$ (MW 340.43): C, 67.04; H, 7.11; N, 16.46. Found: C, 66.48; H, 7.04; N, 16.41.

5-butylpyrazine-2-carbonitrile (**BuCN**). Colorless liquid. Yield: 43%. ^{1}H NMR (500 MHz, $CDCl_3$) δ 8.79 (d, *J* = 1.5 Hz, 1H, H3), 8.54 (d, *J* = 1.5 Hz, 1H, H6), 2.89 (t, *J* = 7.7 Hz, 2H, CH_2), 1.78–1.69 (m, 2H, CH_2), 1.44–1.33 (m, 2H, CH_2), 0.93 (t, *J* = 7.4 Hz, 3H, CH_3). ^{13}C NMR (126 MHz, $CDCl_3$) δ 161.7, 147.4, 145.2, 127.9, 115.7, 35.5, 30.9, 22.2, 13.7. HPLC purity 91.67%. HRMS(ESI$^+$): $[M+H]^+$ calcd. for $C_9H_{12}N_3^+$ (m/z): 162.10257, found 162.10237. CAS: 182306-59-8

5-pentylpyrazine-2-carbonitrile (**PeCN**). Colorless liquid. Yield: 48%. ^{1}H NMR (500 MHz, $CDCl_3$) δ 8.80 (d, *J* = 1.5 Hz, 1H, H3), 8.55 (d, *J* = 1.5 Hz, 1H, H6), 2.92–2.86 (m, 2H, CH_2), 1.82–1.70 (m, 2H, CH_2), 1.47–1.23 (m, 4H, $(CH_2)_2$), 0.88 (t, *J* = 7.0 Hz, 3H, CH_3). ^{13}C NMR (126 MHz, $CDCl_3$) δ 161.7, 147.4, 145.2, 127.9, 115.7, 35.8, 31.3, 28.6, 22.3, 13.8. HPLC purity 98.92%. HRMS(ESI$^+$): $[M+H]^+$ calcd. for $C_{10}H_{14}N_3^+$ (m/z): 176.11822, found 176.11797. CAS: 916333-54-5

5-hexylpyrazine-2-carbonitrile (**HxCN**). Colorless liquid. Yield: 27%. ^{1}H NMR (500 MHz, $CDCl_3$) δ 8.80 (d, *J* = 1.5 Hz, 1H, H3), 8.55 (d, *J* = 1.5 Hz, 1H, H6), 2.89 (t, *J* = 7.8 Hz, 2H, CH_2), 1.80–1.70 (m, 2H, CH_2), 1.41–1.24 (m, 6H, $(CH_2)_3$), 0.87 (t, *J* = 7.1 Hz, 3H, CH_3). ^{13}C NMR (126 MHz, $CDCl_3$) δ 161.7, 147.4, 145.2, 127.9, 115.7, 35.8, 31.4, 28.9, 28.8, 22.4, 14.0. CAS: 916333-55-6

5-heptylpyrazine-2-carbonitrile (**HpCN**). Colorless liquid. Yield: 18%. ^{1}H NMR (500 MHz, $CDCl_3$) δ 8.80 (d, *J* = 1.5 Hz, 1H, H3), 8.55 (d, *J* = 1.5 Hz, 1H, H6), 2.93–2.86 (m, 2H, CH_2), 1.80–1.71 (m, 2H, CH_2), 1.40–1.20 (m, 8H, $(CH_2)_4$), 0.87 (t, *J* = 6.9 Hz, 3H, CH_3). ^{13}C NMR (126 MHz, $CDCl_3$) δ 161.7, 147.4, 145.2, 127.9, 115.7, 35.8, 31.6, 29.1, 28.9, 22.5, 14.0. CAS: 916333-56-7

2.2. Representative ^{1}H and ^{13}C NMR spectra (selected final compounds)

Compound 1: BuPZA

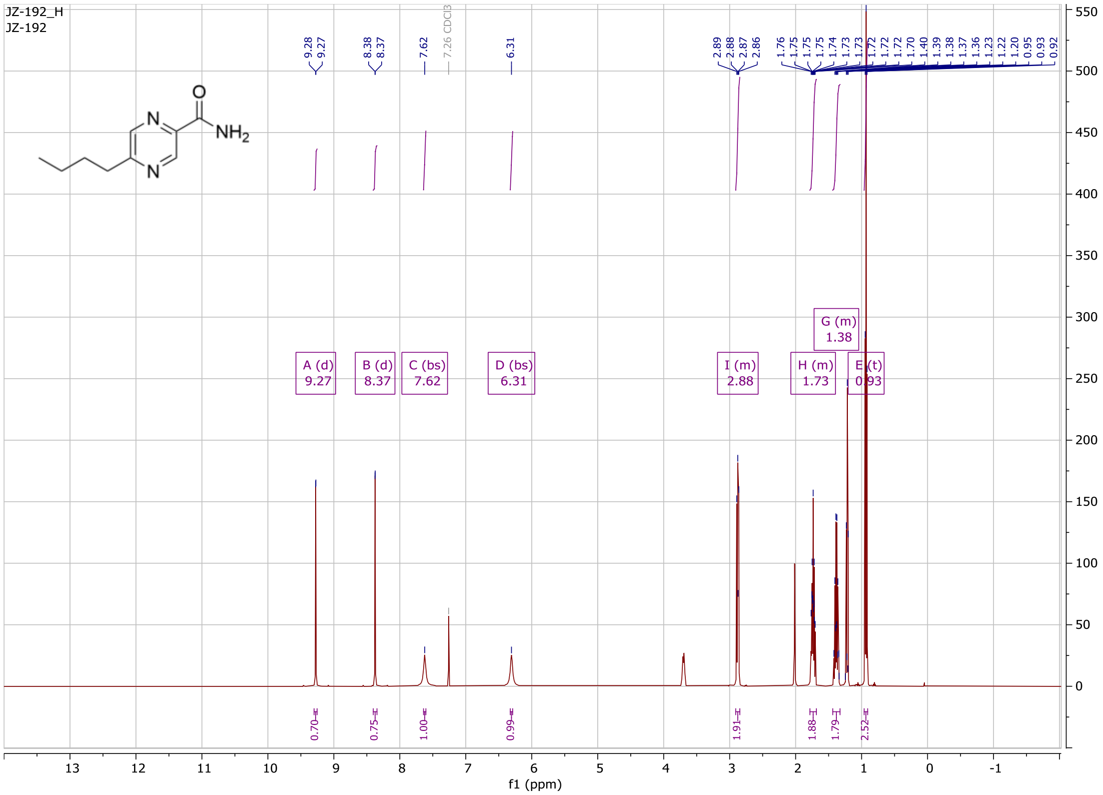

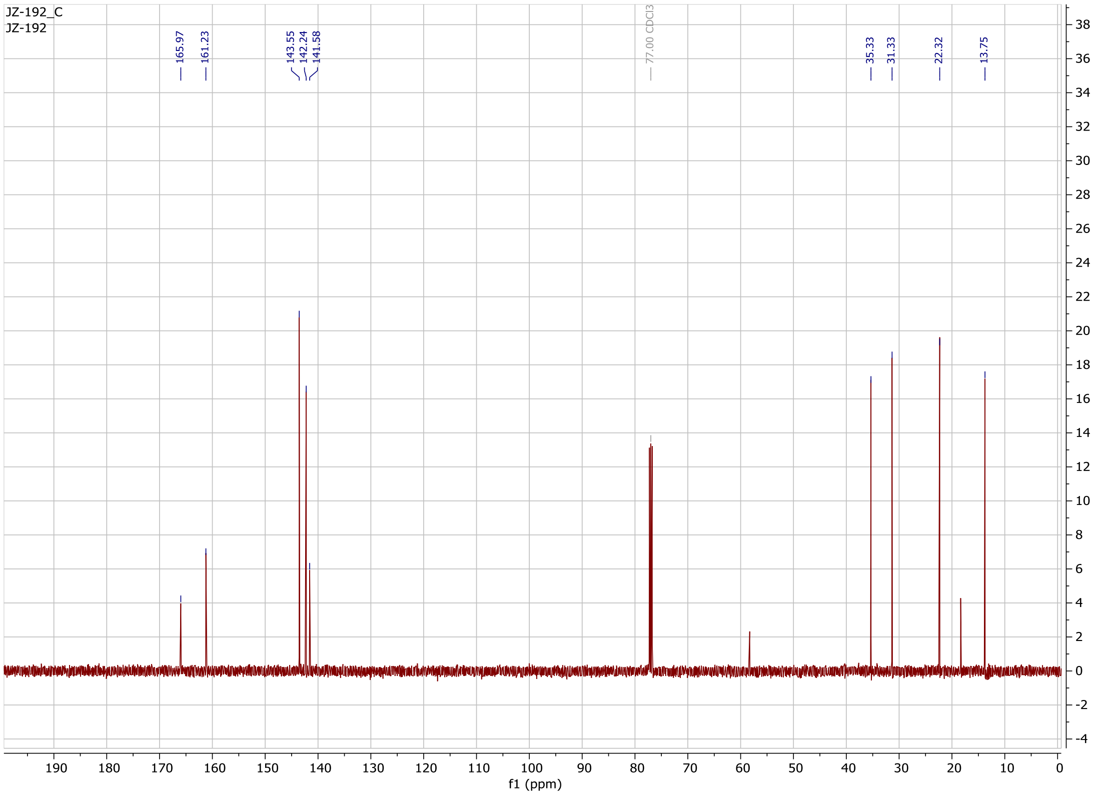

Figure S1. ^{1}H-NMR (top) and ^{13}C-NMR (bottom) spectra of compound **1**.

Compound 4: HpPZA

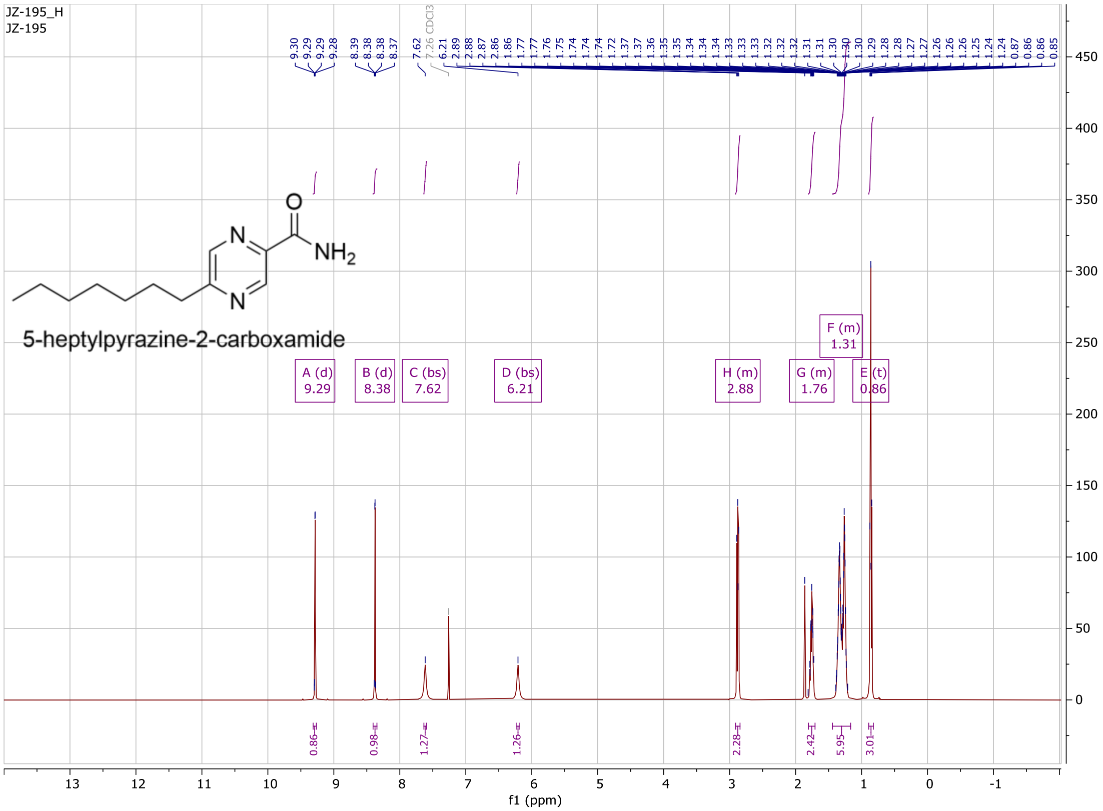

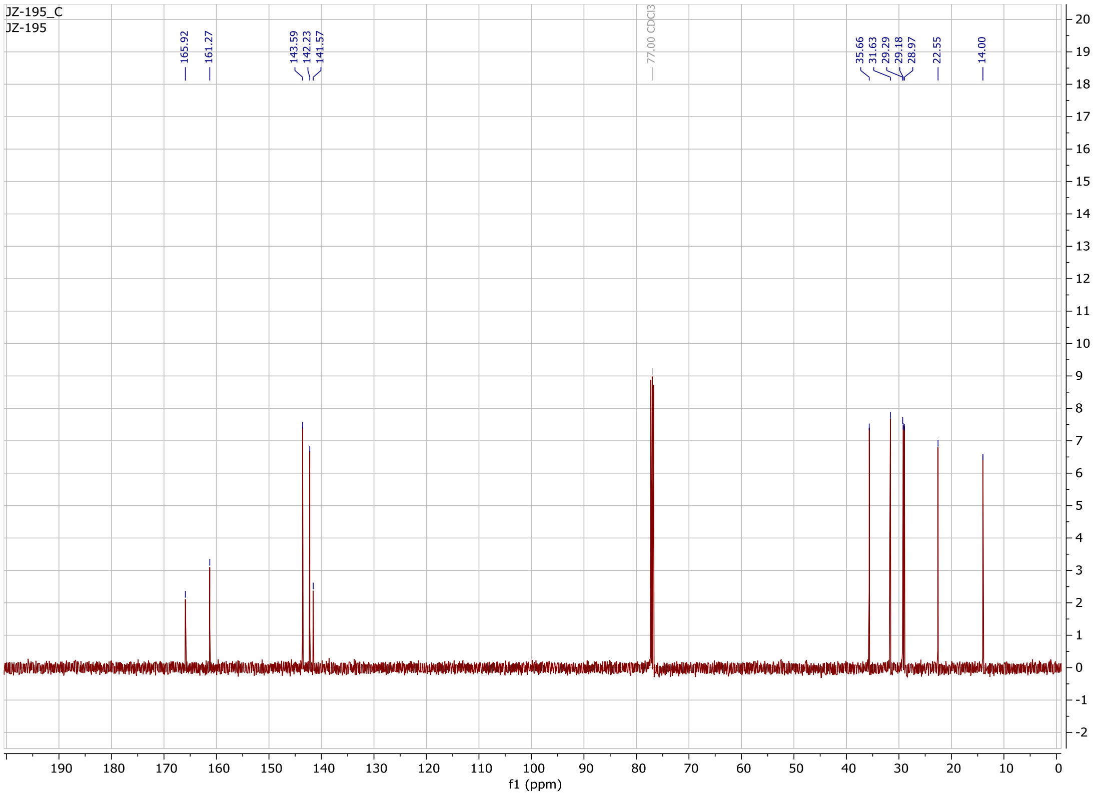

Figure S2. ^{1}H-NMR (top) and ^{13}C-NMR (bottom) spectra of compound **4**.

Compound 6: PePOA

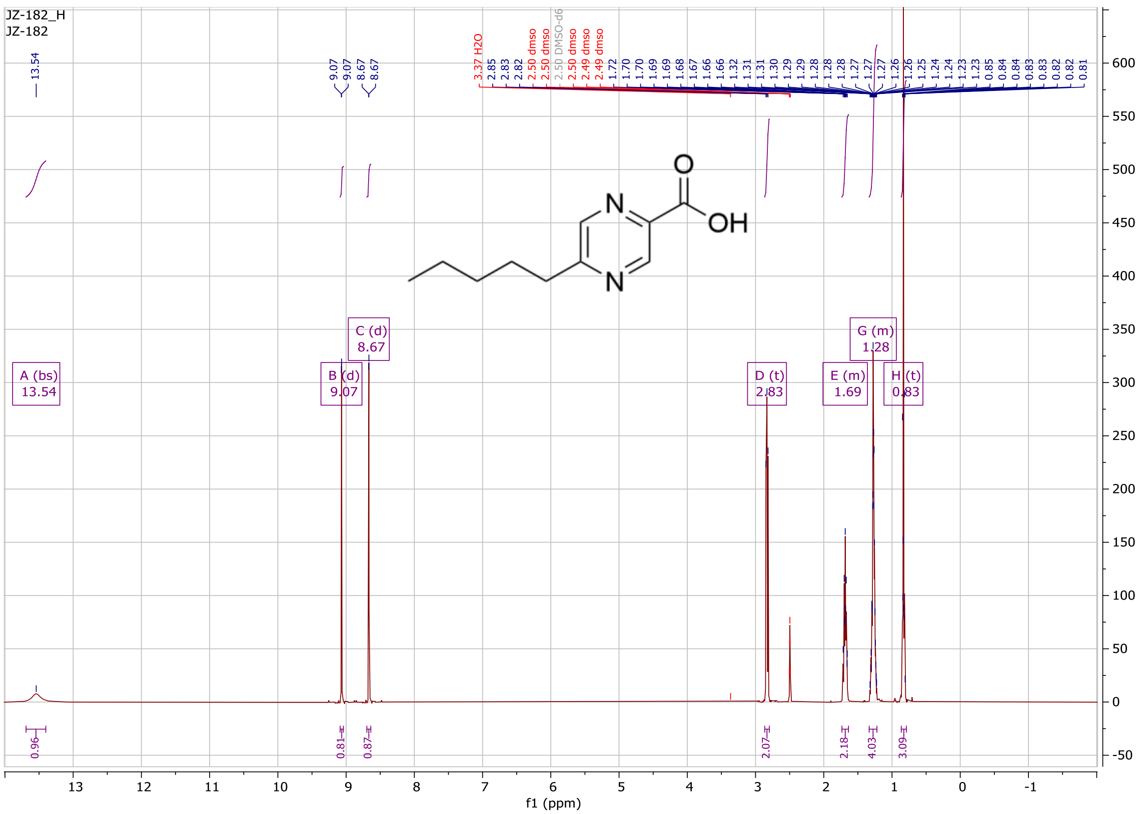

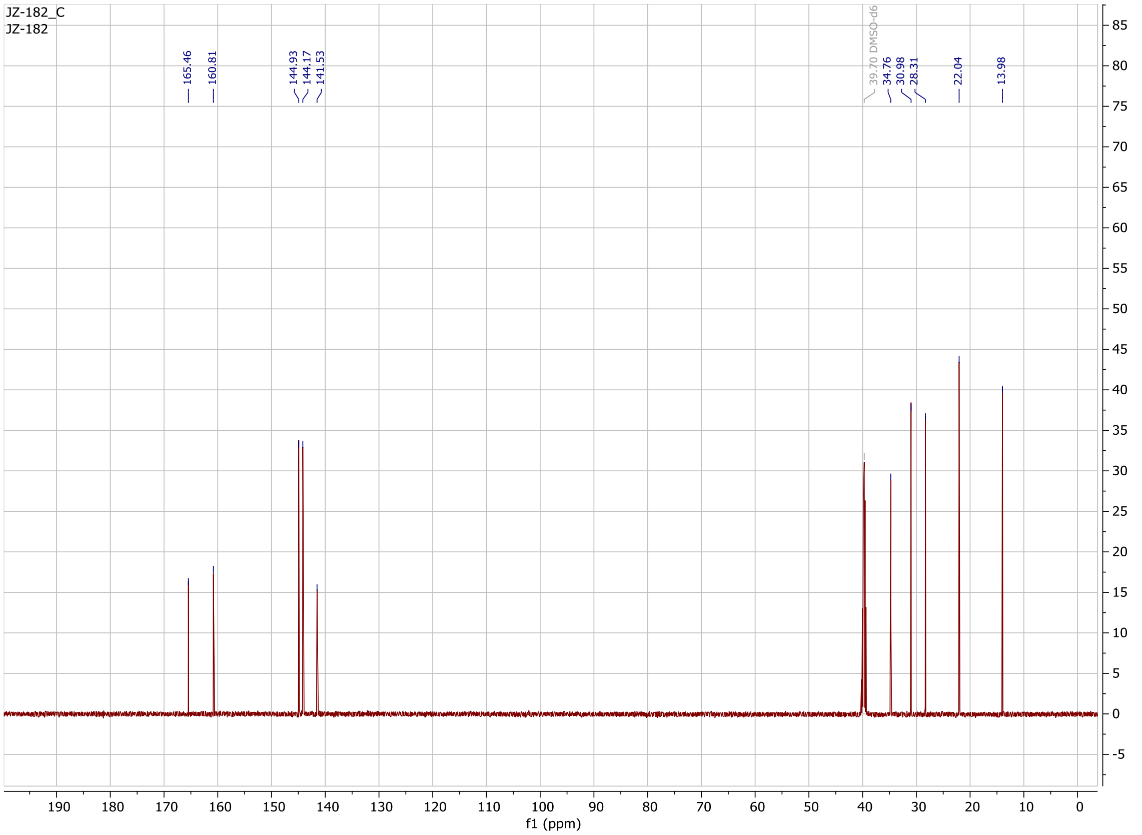

Figure S3. ^{1}H-NMR (top) and ^{13}C-NMR (bottom) spectra of compound **6**.

Compound BuCN: butylpyrazine-2-carbonitrile

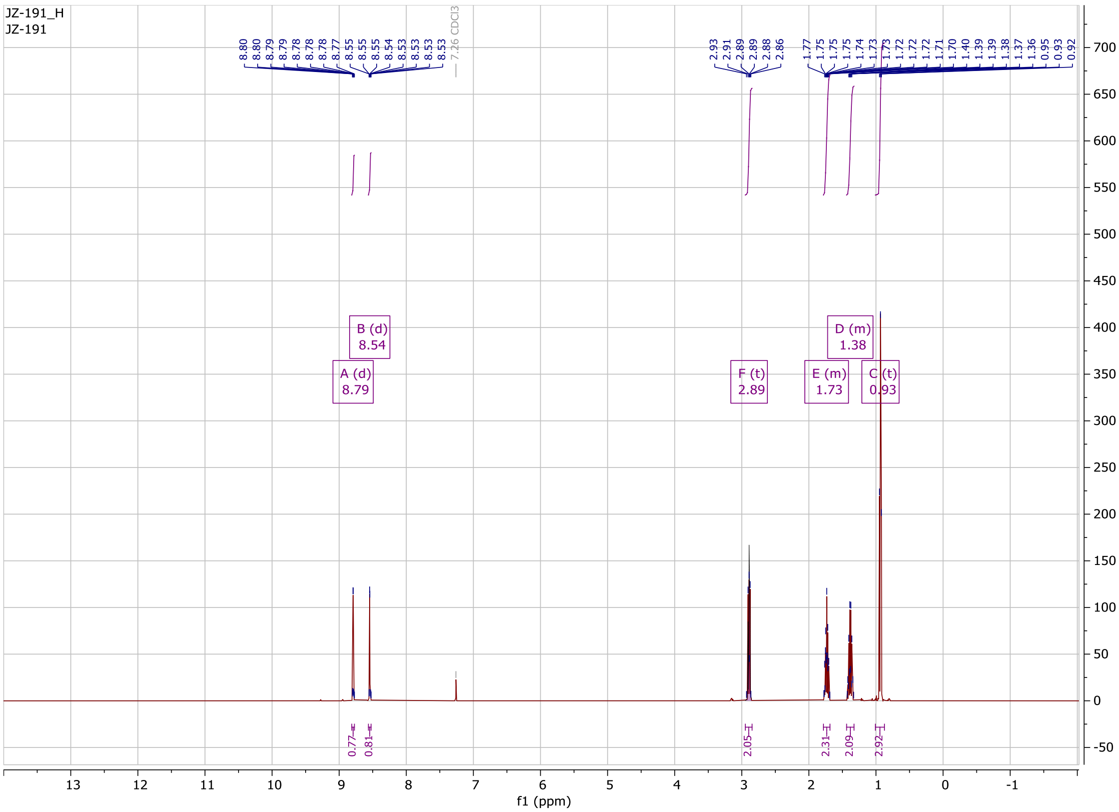

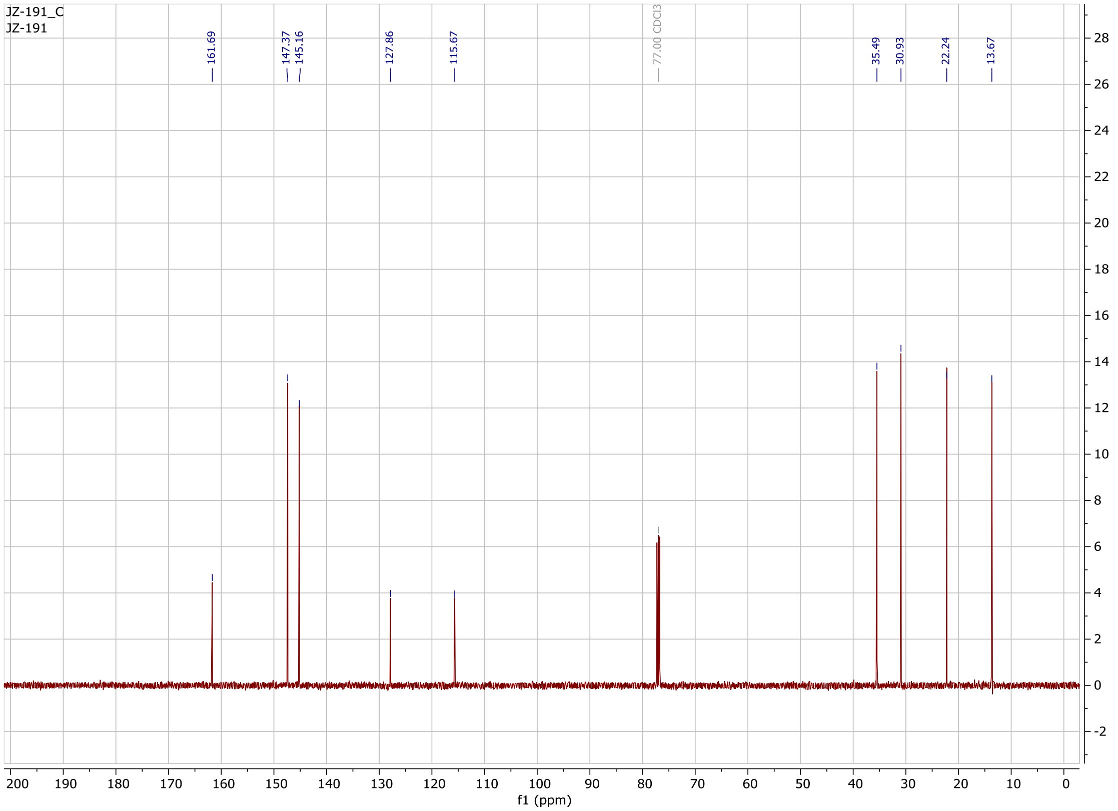

Figure S4. ^{1}H-NMR (top) and ^{13}C-NMR (bottom) spectra of compound BuCN.

Compound 34: 5-pentanamido-*N*-phenylpyrazine-2-carboxamide

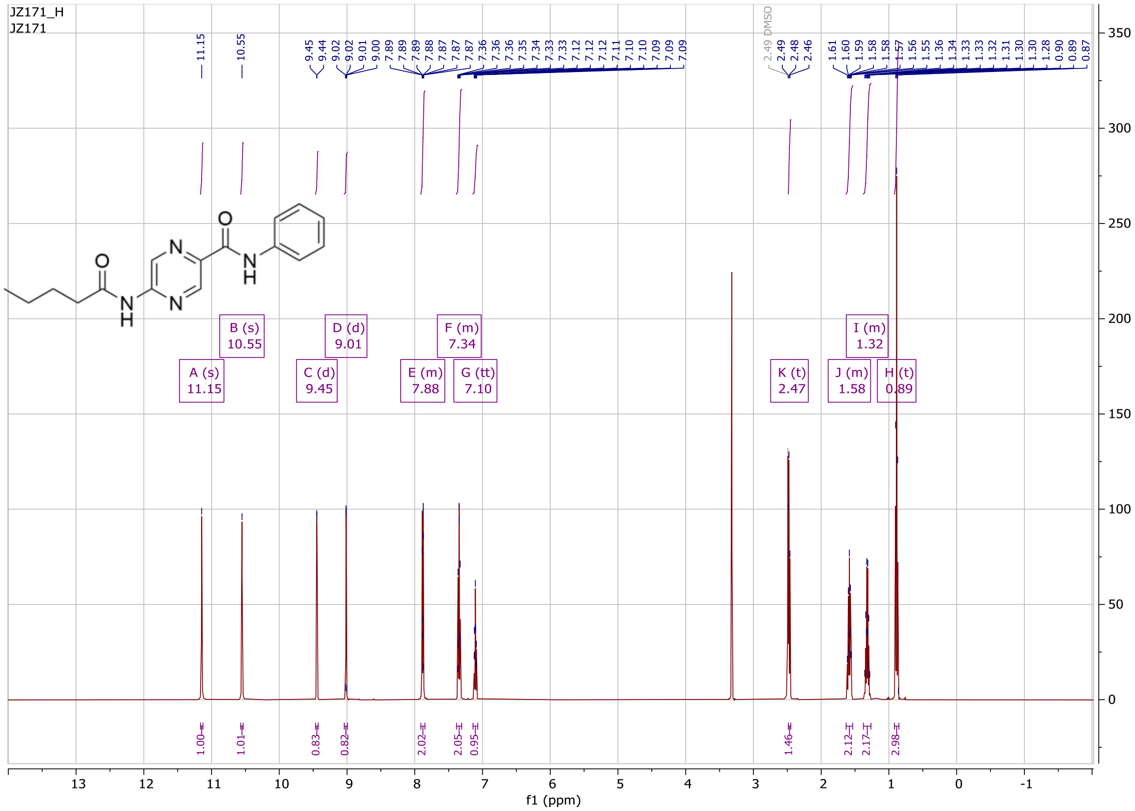

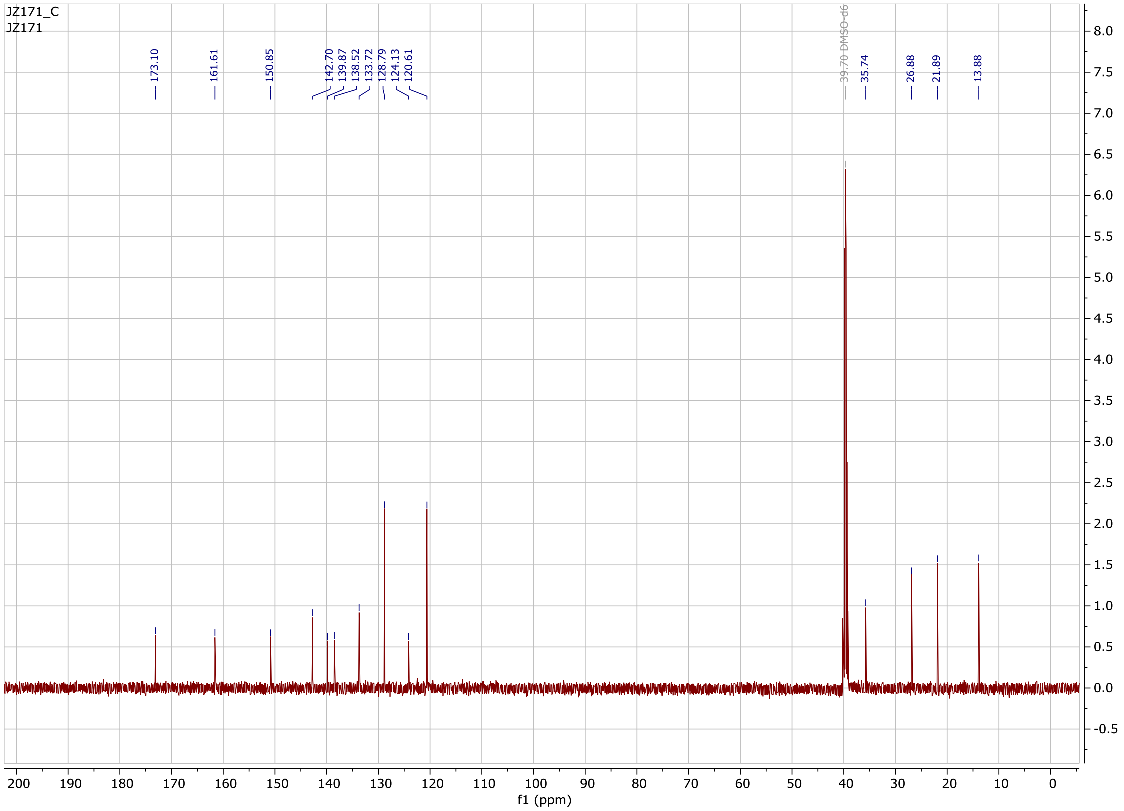

Figure S5. ^{1}H-NMR (top) and ^{13}C-NMR (bottom) spectra of compound **34**.

Compound 10: 5-pentanamidopyrazine-2-carboxamide

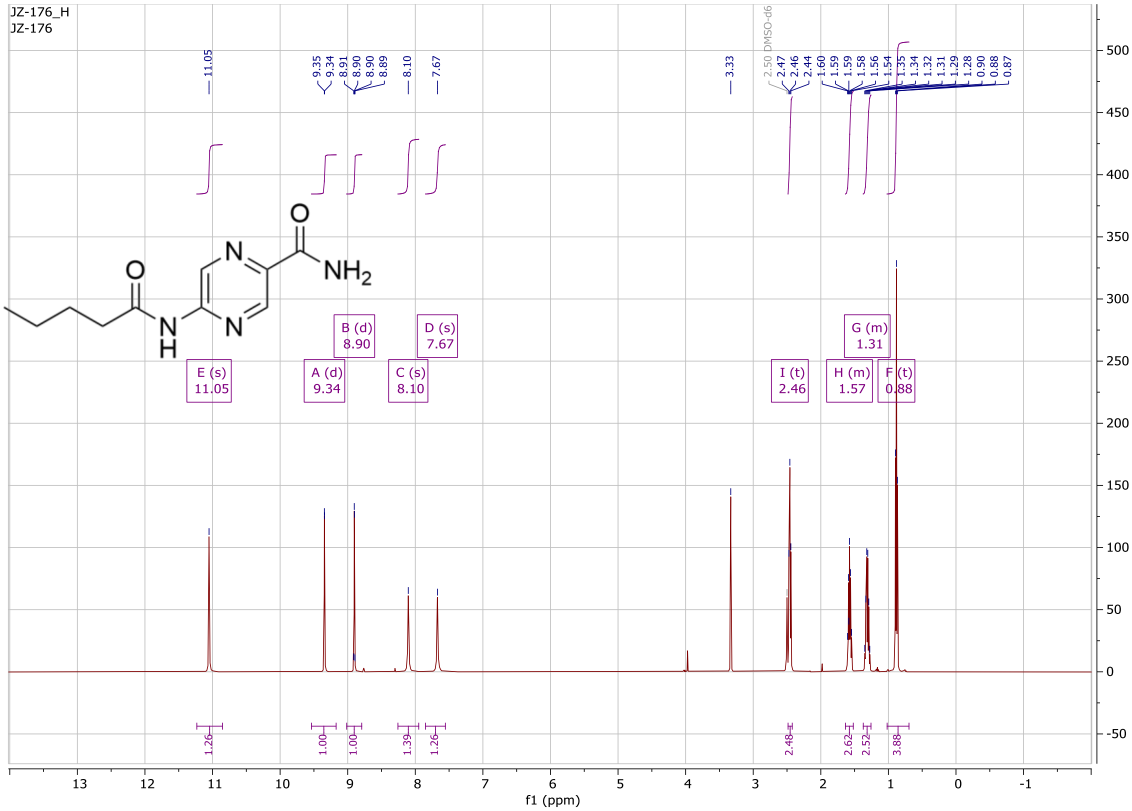

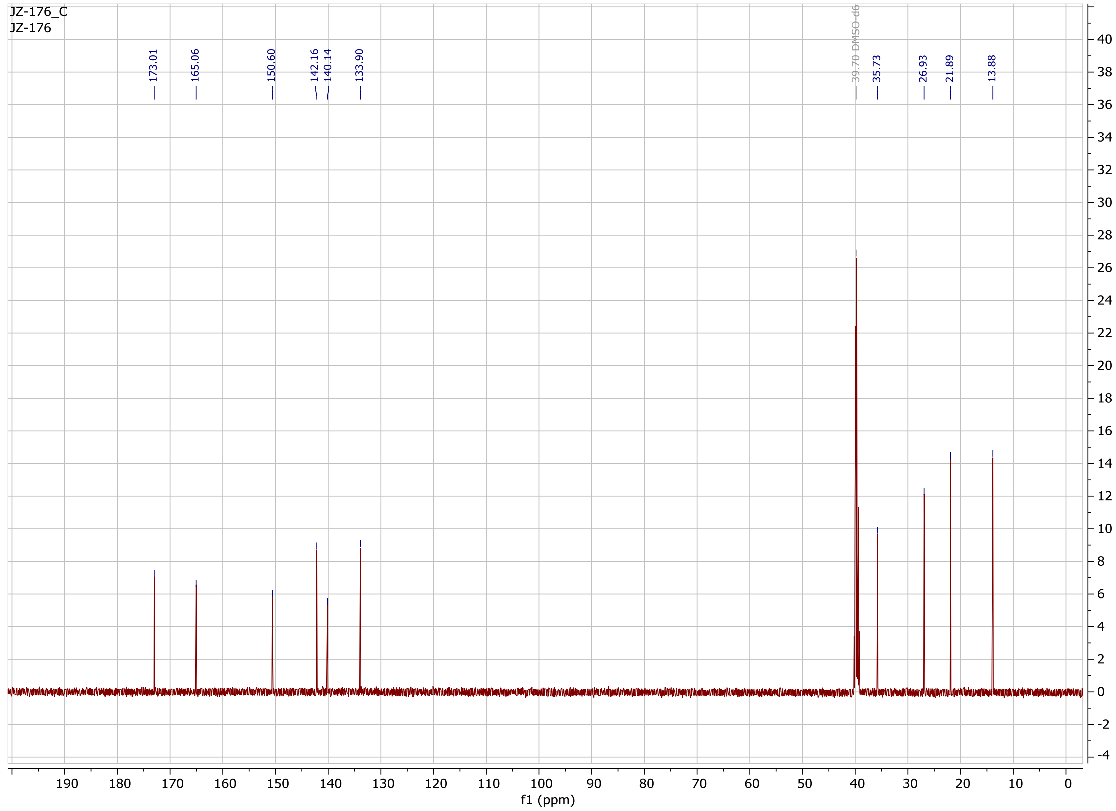

Figure S6. ^{1}H-NMR (top) and ^{13}C-NMR (bottom) spectra of compound **10**.

Compound 24: **5-hexyl-*N*-(3-(trifluoromethyl)phenyl)pyrazine-2-carboxamide**

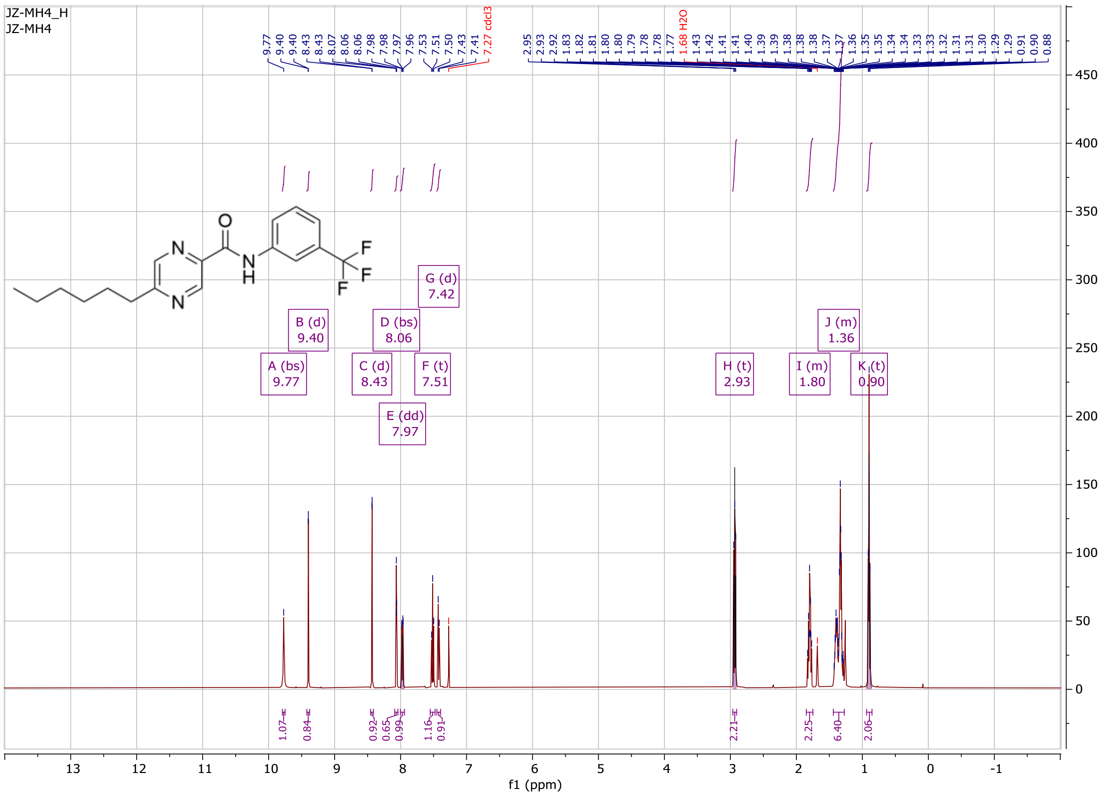

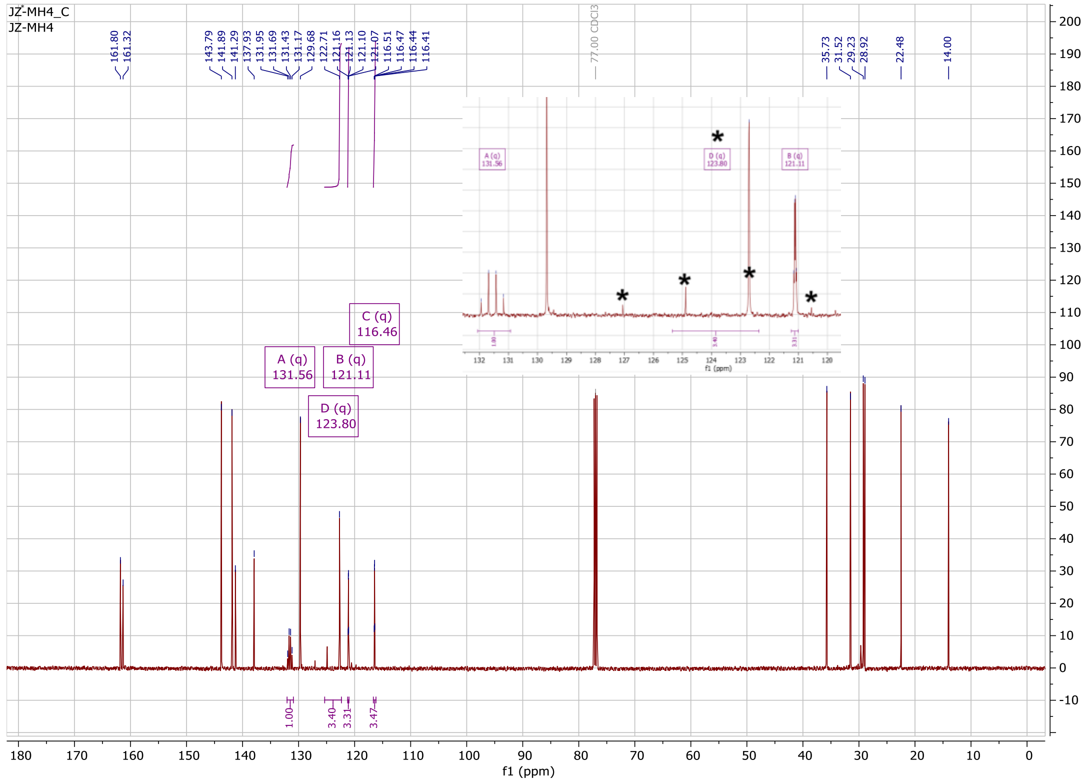

Figure S7. ^{1}H-NMR (top) and ^{13}C-NMR (bottom) spectra of compound **24**.

2.3. HPLC-HRMS analysis

Methodology

The compounds were analyzed in LC-MS system to obtain high-resolution mass spectra. Briefly, a reverse phase C18 column Kinetex EVO (Phenomenex, Torrance, USA) was used as a stationary phase, and purified water with 0.1% formic acid (mobile phase A) and LC-MS grade acetonitrile with 0.1% formic acid (mobile phase B) were used as the mobile phases. Gradient elution was set up to determine the mass spectra. The method started with 5% B for 0.3 min, the gradient then switched to 100% B in the third min, remained at 100% B for 0.7 min, and then reverted to 5% B with equilibration for 3.5 min. The total run time of the method was 7.5 min. The column temperature was kept constant at 27 ℃, the flow of the mobile phase was 0.5 mL/min, and the injection volume was 1 μL.

Compound 1: BuPZA

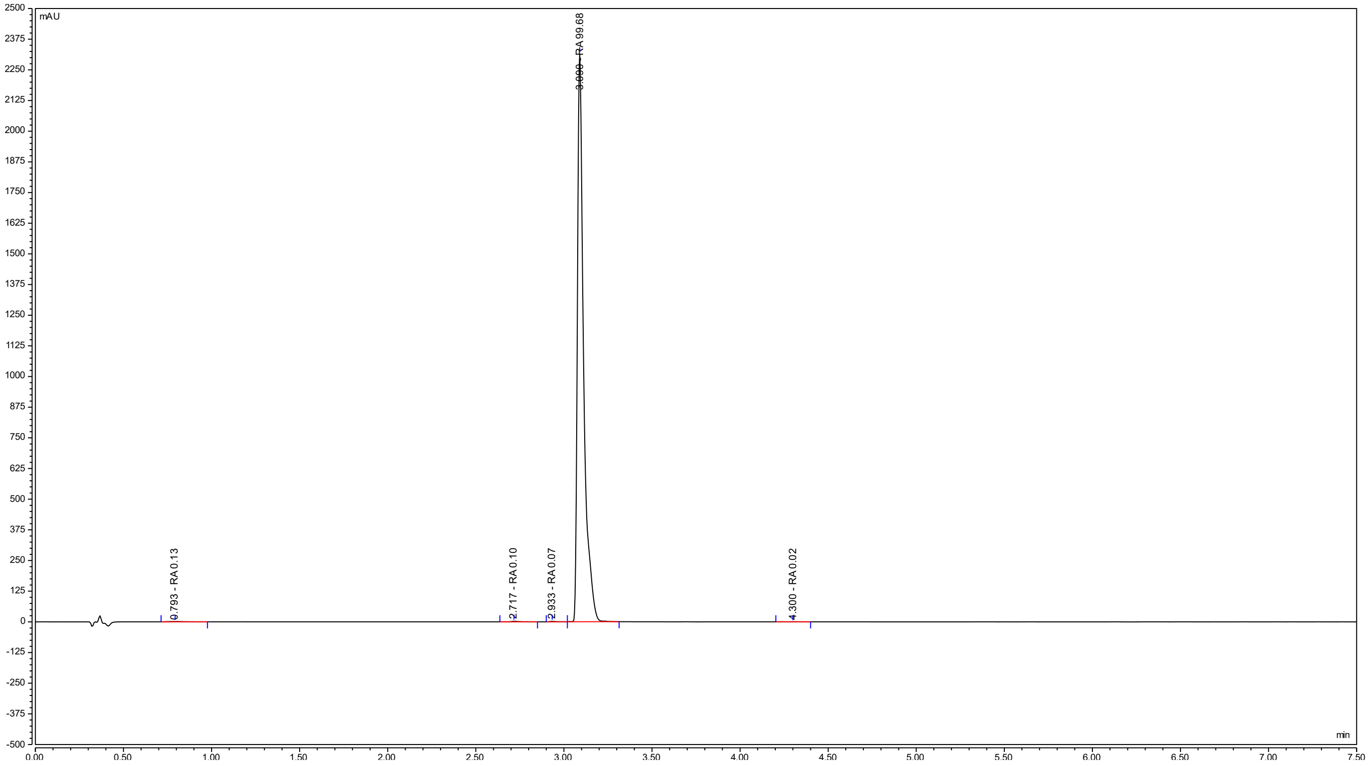

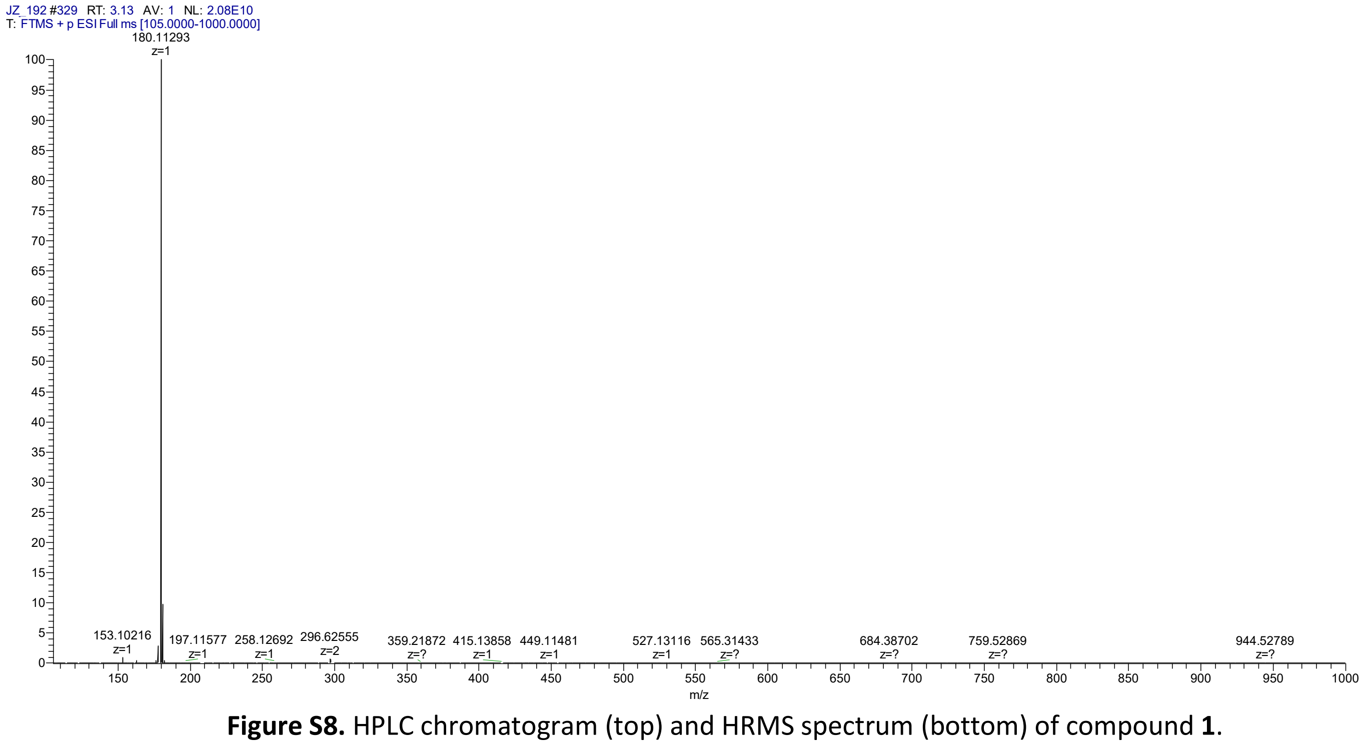

Figure S8. HPLC chromatogram (top) and HRMS spectrum (bottom) of compound **1**.

Compound 2: PePZA

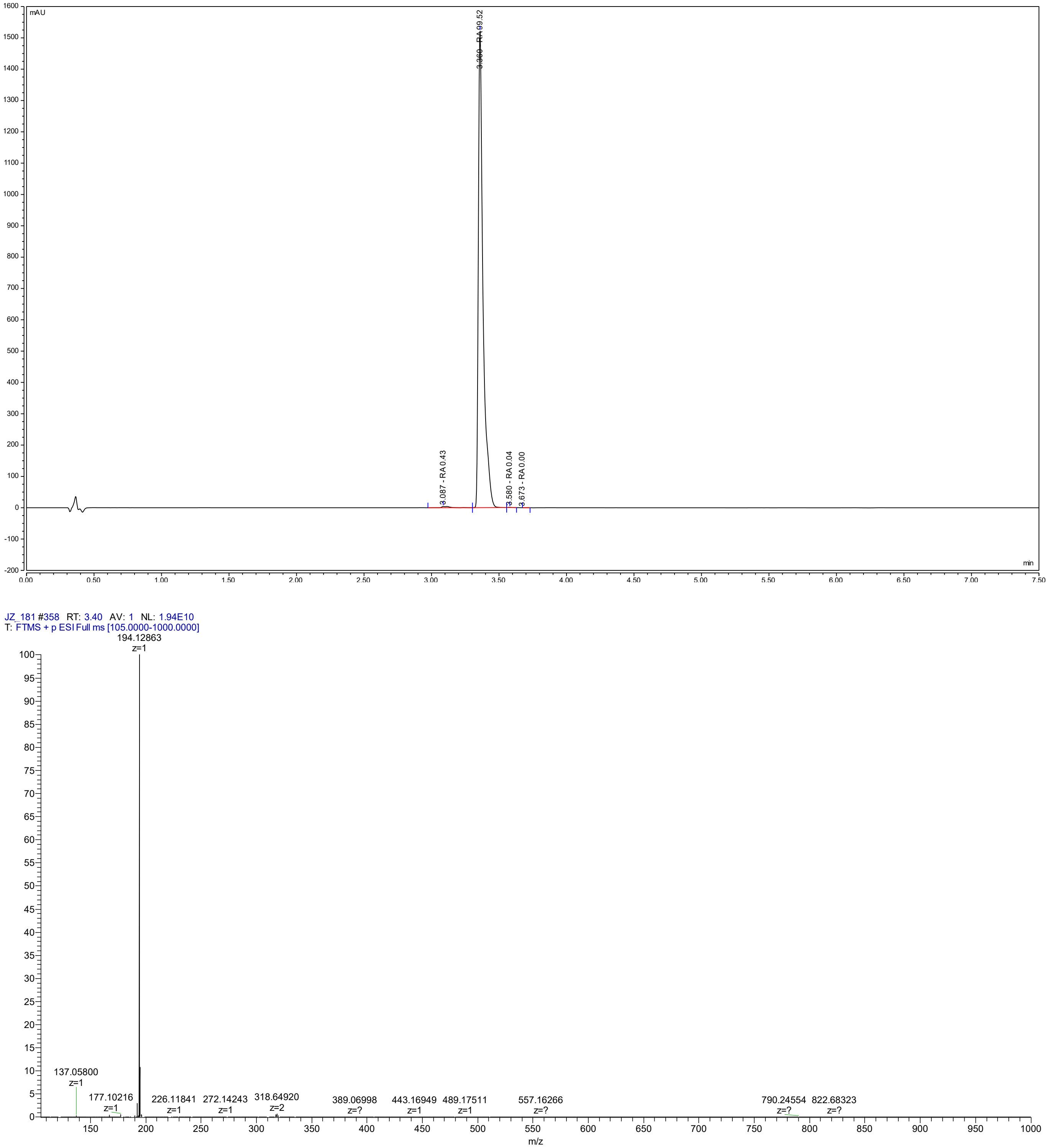

Figure S9. HPLC chromatogram (top) and HRMS spectrum (bottom) of compound **2**.

Compound 6: PePOA

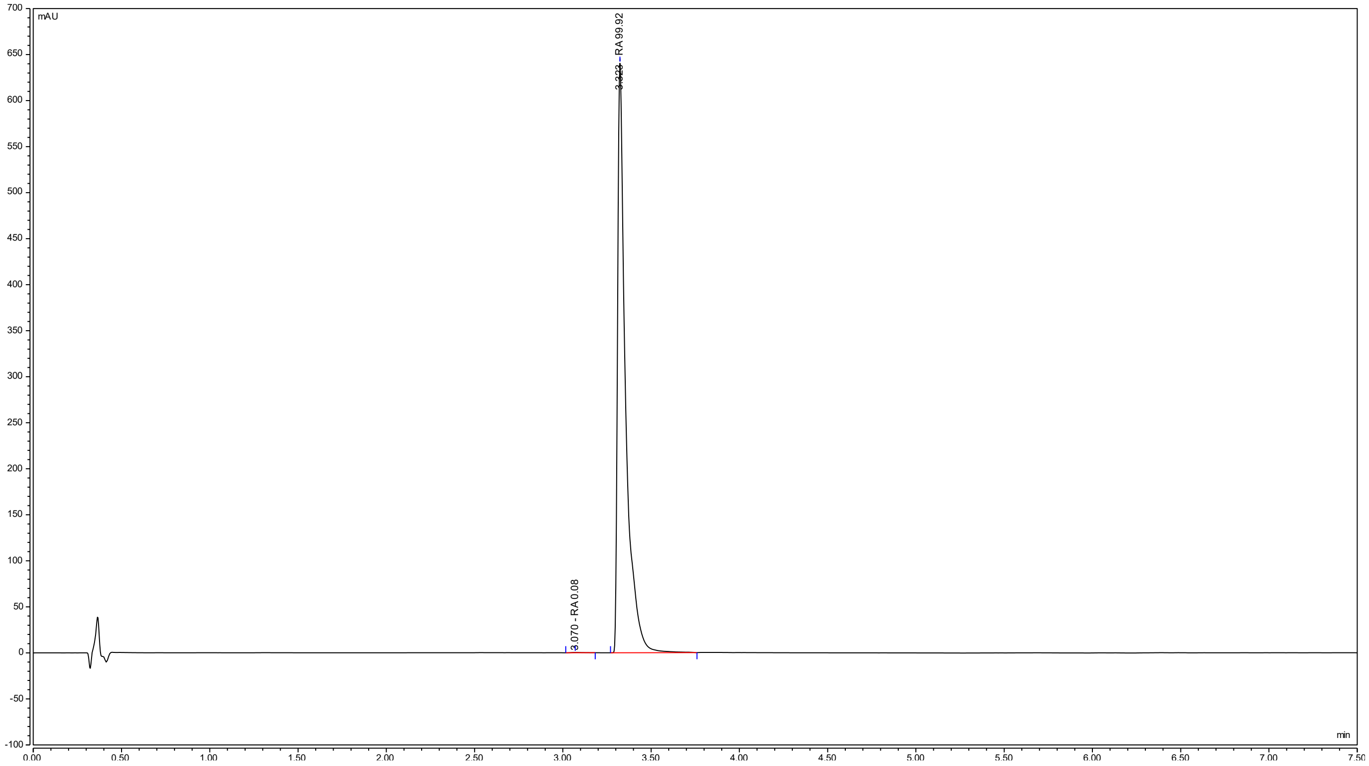

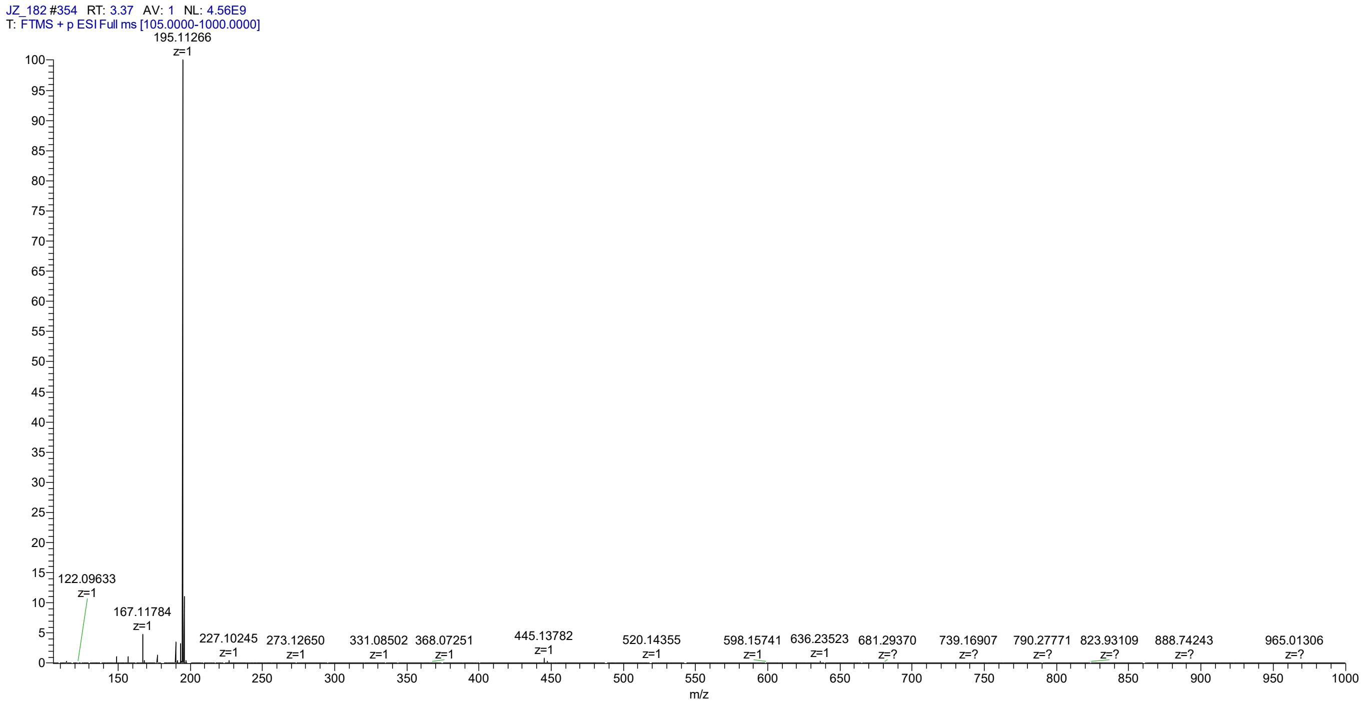

Figure S10. HPLC chromatogram (top) and HRMS spectrum (bottom) of compound **6**.

Compound BuCN: butylpyrazine-2-carbonitrile

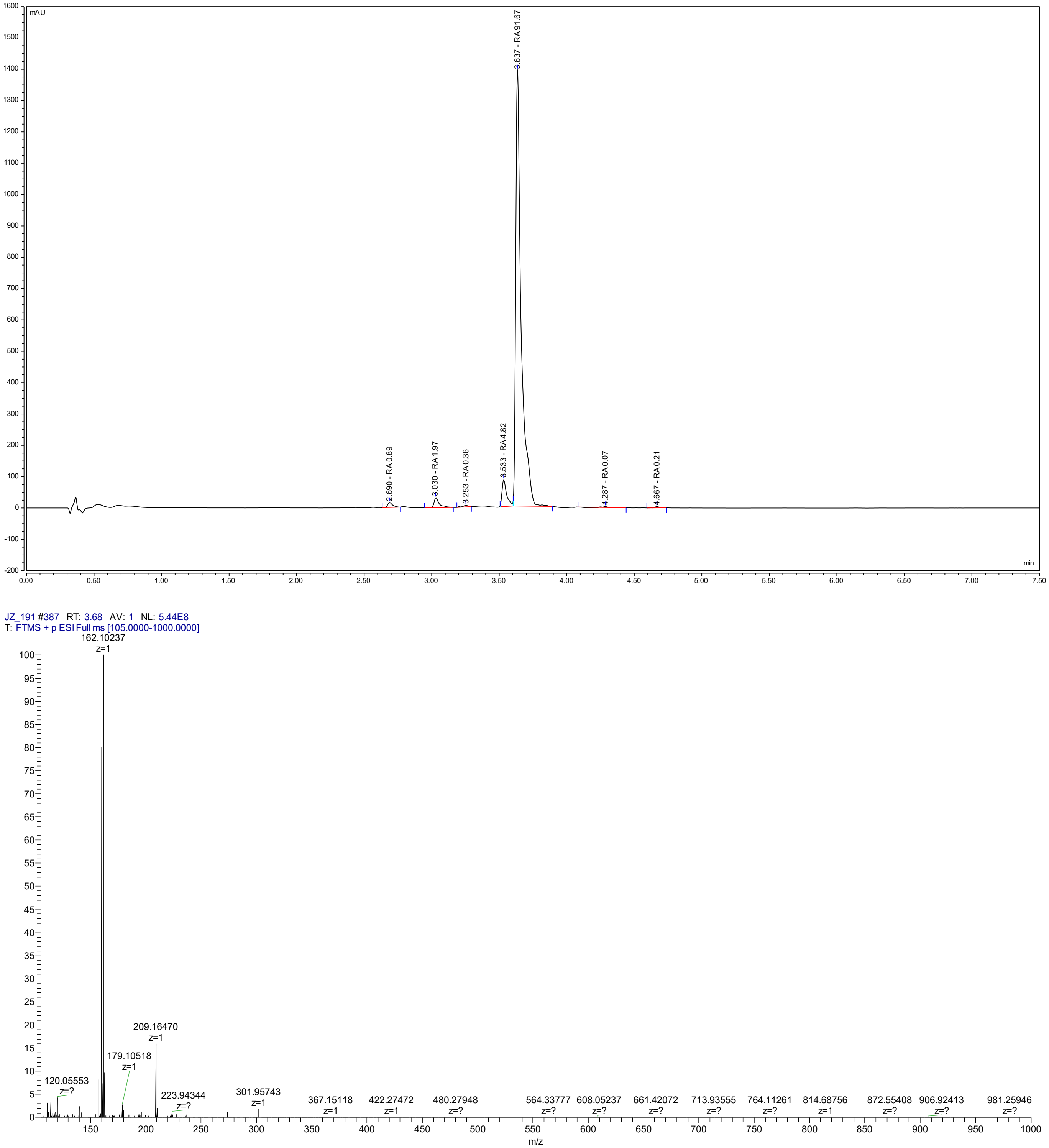

Figure S11. HPLC chromatogram (top) and HRMS spectrum (bottom) of compound **BuCN**.

Compound PeCN: pentylpyrazine-2-carbonitrile

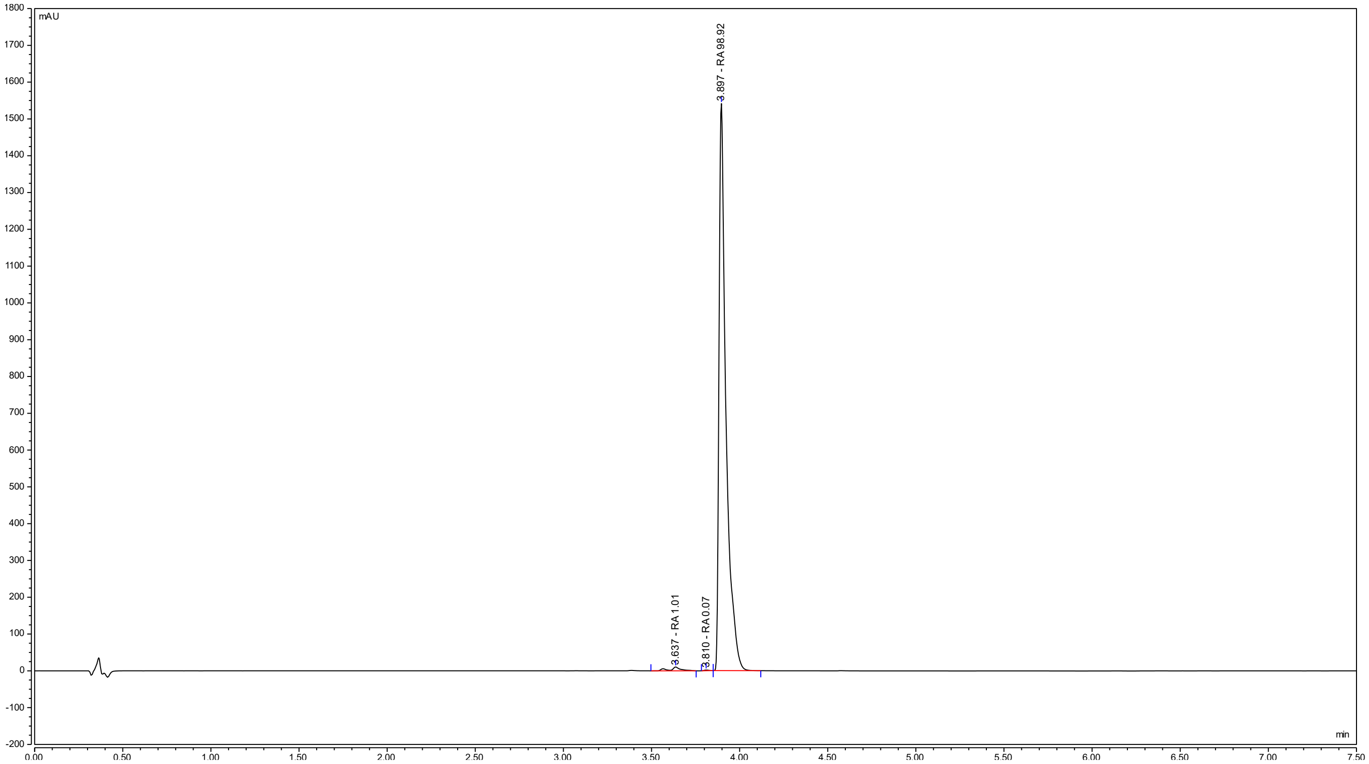

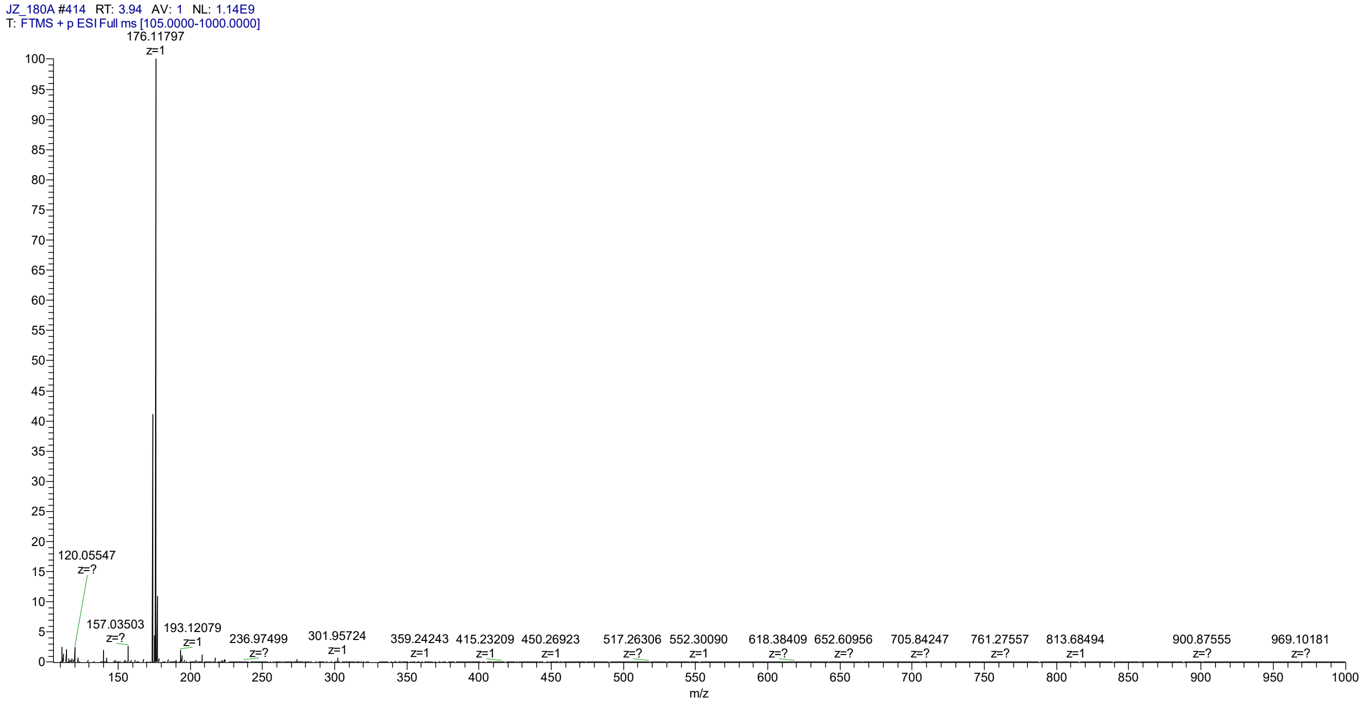

Figure S12. HPLC chromatogram (top) and HRMS spectrum (bottom) of compound **PeCN**.

2.4. SDS-PAGE analysis of mycobacterial pyrazinamidase Mtb-PncA

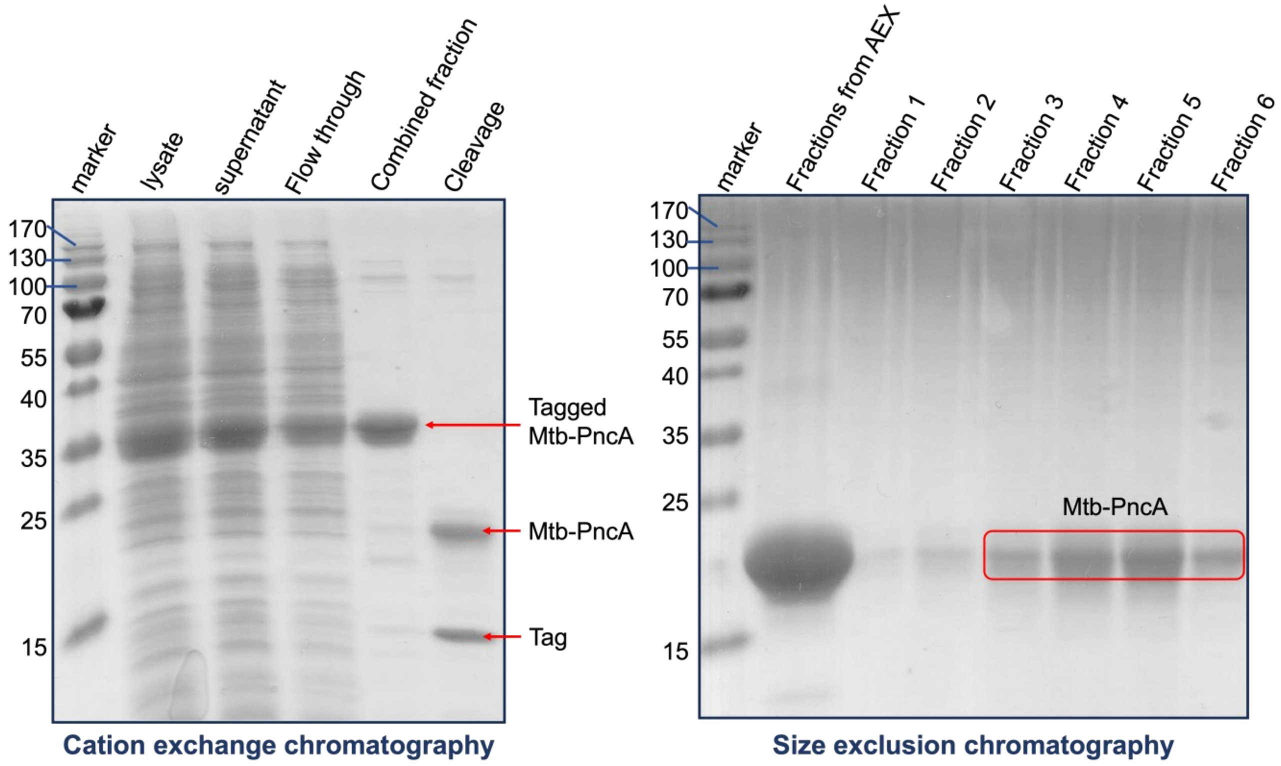

Figure S13. The SDS-PAGE gel stained with Coomassie blue in purification procedure of Mtb-PncA. Left: The SDS-PAGE gel of *Mtb*-PncA in the first step cation exchange chromatography; Right: The SDS-PAGE gel of Mtb-PncA in the size exclusion chromatography. AEX: anion exchange chromatography.

3. Additional results

3.1. MABA assay on MDR strains of *M. tuberculosis* (photos)

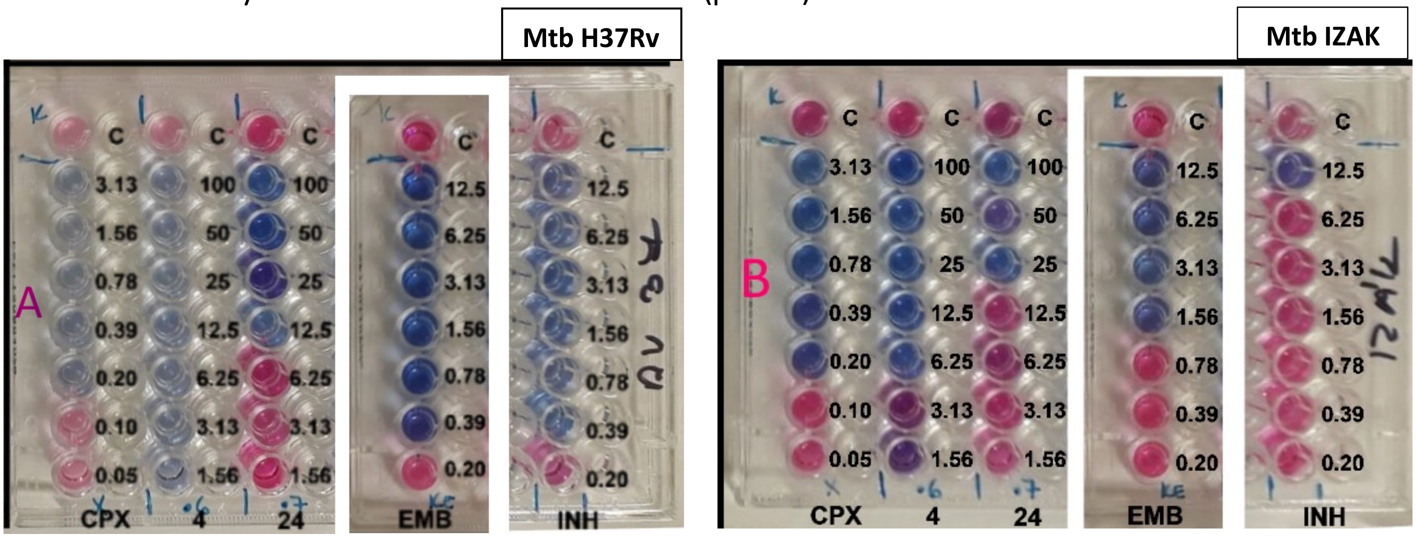

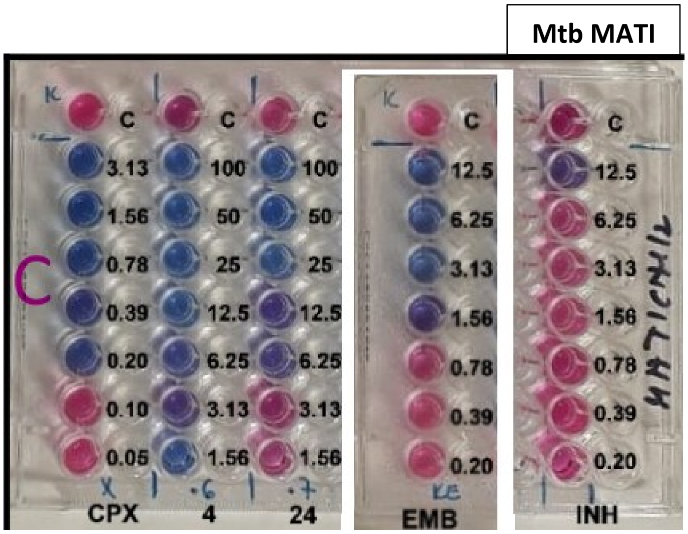

MIC determination [µg/mL]

Strain	4	24	INH	CIP	EMB
***Mtb* H37Rv**	≤1.56	12.5	0.39	0.2	0.39
***Mtb* IZAK**	6.25	25	12.5 **R**	0.2	1.56
***Mtb* MATI**	≤1.56	6.25	12.5 **R**	0.1	1.56

R – resistant

Figure S14. Photos of MABA assay on MDR strains of *M. tuberculosis*. **A** – Collection strain *M. tuberculosis* H37Rv. **B** – MDR clinical isolate strain of *M. tuberculosis* (lab. code IZAK) **C** – MDR clinical isolate strain of *M. tuberculosis* (lab. code MATI). Concentrations of compounds are indicated to the right of the wells. 'C' in the top line is untreated control (growth control). CPX – ciprofloxacin, EMB – ethambutol, INH – isoniazid, 4 – compound **4**, 24 - compound **24**. Pink color indicates growth, blue color indicates no growth. Only wells relevant to this study are shown. Other wells (testing unrelated compounds) were cropped for clarity; no image-level adjustments affecting the displayed wells were made.

3.2. Antimycobacterial activity against non-tuberculous mycobacteria

Table S4. Antimycobacterial activity of prepared compounds against non-tuberculous mycobacteria.

Structure	Code	Alkyl	R	M. avium ATCC 15769 MIC [µg/mL]	*M. kansasii* ATCC 12478 MIC [µg/mL]	*M. aurum* ATCC 23366 MIC [µg/mL]	*M. smegmatis* ATCC 607 MIC [µg/mL]
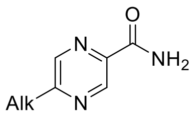 Series A	**1**	Butyl	-	>100	>100	>500	250
	**2**	Pentyl	-	>100	>100	500	>500
	**3**	Hexyl	-	>100	25	>500	>500
	**4**	Heptyl	-	12.5	>100	>500	>500
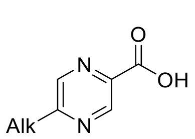	**5**	Butyl	-	>100	>100	n.t.	>500
	**6**	Pentyl	-	>100	>100	125	>500
	**7**	Hexyl	-	>100	>100	125	250
	**8**	Heptyl	-	100	>100	62.5	125
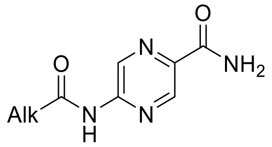 Series B	**9**	Propyl	-	>100	>100	>500	>500
	**10**	Butyl	-	>100	100	>500	>500
	**11**	Pentyl	-	>100	6.25	31.25	>500
	**12**	Hexyl	-	>100	6.25	15.625	>500
	**13**	Heptyl	-	>100	50	7.81	>500
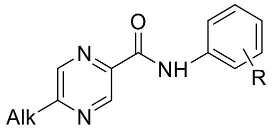 Series C	**14**	Butyl	H	>100	>100	>500	>500
	**15**	Pentyl	H	>100	25	>500	>500
	**16**	Hexyl	H	>50	>50	>250	>250
	**17**	Heptyl	H	>100	>100	>500	>500
	**18**	Butyl	2-Cl	>100	>100	>500	>500
	**19**	Pentyl	2-Cl	>100	>100	>500	>500
	**20**	Hexyl	2-Cl	>50	>50	>125	>125
	**21**	Heptyl	2-Cl	>50	>50	>250	>250
	**22**	Butyl	3-CF_3	>100	>100	>500	250
	**23**	Pentyl	3-CF_3	>100	12.5	n.t.	>500
	**24**	Hexyl	3-CF_3	>100	25	31.25–62.50	3.91
	**25**	Heptyl	3-CF_3	>100	12.5	62.5	62.5
	**26**	Butyl	4-CH_3	>100	>100	>250	>250
	**27**	Pentyl	4-CH_3	>100	>100	>250	>250
	**28**	Heptyl	4-CH_3	>100	>100	62.5	>125
	**29**	Butyl	4-OH	>100	50	>500	>500
	**30**	Pentyl	4-OH	>100	50	>125	>125
	**31**	Hexyl	4-OH	>100	>100	>500	>500
	**32**	Heptyl	4-OH	>100	>100	>500	>500
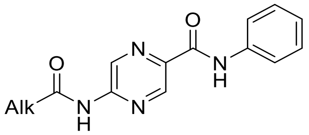 Series D	**33**	Propyl	-	>100	25	>500	>500
	**34**	Butyl	-	>100	6.25	>500	>500
	**35**	Pentyl	-	>100	6.25	250	>500
	**36**	Hexyl	-	>100	6.25	>500	>500
	**37**	Heptyl	-	>100	>100	>250	>500
Standards	INH	-	-	6.25–12.5	6.25–12.5	3.91–7.81	7.81–15.625
	RIF	-	-	n.t.	n.t.	0.39–0.78	12.5–25
	CIP	-	-	n.t.	n.t.	0.0156	0.0625

INH – isoniazid, RIF – rifampicin, CIP – ciprofloxacin; n.t. – not tested

3.3. Antibacterial screening of compounds of series C

Compounds **14**–**32** were screened for *in vitro* **antibacterial activity** (Table S5). No activity was detected up to the highest tested concentration, which was dependent on the solubility of the compound.

Table S5. Results of antibacterial activity screening of compounds **14**–**32**

**STRAIN** (code)		**COMPOUND** – **MIC** (μM)									
		**14**	**15**	**16**	**17**	**18**	**19**	**20**	**21**	**22**	**23**
**SA**	24h	>500	>500	>500	>500	>500	>500	>250	>250	>500	>500
	48h	>500	>500	>500	>500	>500	>500	>250	>250	>500	>500
**MRSA**	24h	>500	>500	>500	>500	>500	>500	>250	>250	>500	>500
	48h	>500	>500	>500	>500	>500	>500	>250	>250	>500	>500
**SE***	24h	>500	>500	>500	>500	>500	>500	>250	>250	>500	>500
	48h	>500	>500	>500	>500	>500	>500	>250	>250	>500	>500
**EF**	24h	>500	>500	>500	>500	>500	>500	>250	>250	>500	>500
	48h	>500	>500	>500	>500	>500	>500	>250	>250	>500	>500
**EC**	24h	>500	>500	>500	>500	>500	>500	>250	>250	>500	>500
	48h	>500	>500	>500	>500	>500	>500	>250	>250	>500	>500
**KP***	24h	>500	>500	>500	>500	>500	>500	>250	>250	>500	>500
	48h	>500	>500	>500	>500	>500	>500	>250	>250	>500	>500
**SEMA***	24h	>500	>500	>500	>500	>500	>500	>250	>250	>500	>500
	48h	>500	>500	>500	>500	>500	>500	>250	>250	>500	>500
**PA**	72h	>500	>500	>500	>500	>500	>500	>250	>250	>500	>500
	120h	>500	>500	>500	>500	>500	>500	>250	>250	>500	>500

<table>
<tr><th colspan="2">STRAIN (code)</th><th colspan="10">COMPOUND – MIC (μM)</th></tr>
<tr><th></th><th></th><th>24</th><th>25</th><th>26</th><th>27</th><th>28</th><th>29</th><th>30</th><th>31</th><th>32</th><th></th></tr>
<tr><td>SA</td><td>24h</td><td>>500</td><td>>500</td><td>>500</td><td>>500</td><td>|</td><td>>500</td><td>|</td><td>|</td><td>>500</td><td></td></tr>
<tr><td></td><td>48h</td><td>>500</td><td>>500</td><td>>500</td><td>>500</td><td>|</td><td>>500</td><td>|</td><td>|</td><td>>500</td><td></td></tr>
<tr><td>MRSA</td><td>24h</td><td>>500</td><td>>500</td><td>>500</td><td>>500</td><td>|</td><td>>500</td><td>|</td><td>|</td><td>>500</td><td></td></tr>
<tr><td></td><td>48h</td><td>>500</td><td>>500</td><td>>500</td><td>>500</td><td>|</td><td>>500</td><td>|</td><td>|</td><td>>500</td><td></td></tr>
<tr><td>SE*</td><td>24h</td><td>>500</td><td>>500</td><td>>500</td><td>>500</td><td>|</td><td>>500</td><td>|</td><td>|</td><td>>500</td><td></td></tr>
<tr><td></td><td>48h</td><td>>500</td><td>>500</td><td>>500</td><td>>500</td><td>|</td><td>>500</td><td>|</td><td>|</td><td>>500</td><td></td></tr>
<tr><td>EF</td><td>24h</td><td>>500</td><td>>500</td><td>>500</td><td>>500</td><td>n.t.</td><td>>500</td><td>n.t.</td><td>n.t.</td><td>>500</td><td></td></tr>
<tr><td></td><td>48h</td><td>>500</td><td>>500</td><td>>500</td><td>>500</td><td>|</td><td>>500</td><td>|</td><td>|</td><td>>500</td><td></td></tr>
<tr><td>EC</td><td>24h</td><td>>500</td><td>>500</td><td>>500</td><td>>500</td><td>|</td><td>>500</td><td>|</td><td>|</td><td>>500</td><td></td></tr>
<tr><td></td><td>48h</td><td>>500</td><td>>500</td><td>>500</td><td>>500</td><td>|</td><td>>500</td><td>|</td><td>|</td><td>>500</td><td></td></tr>
<tr><td>KP*</td><td>24h</td><td>>500</td><td>>500</td><td>>500</td><td>>500</td><td>|</td><td>>500</td><td>|</td><td>|</td><td>>500</td><td></td></tr>
<tr><td></td><td>48h</td><td>>500</td><td>>500</td><td>>500</td><td>>500</td><td>|</td><td>>500</td><td>|</td><td>|</td><td>>500</td><td></td></tr>
<tr><td>SEMA*</td><td>24h</td><td>>500</td><td>>500</td><td>>500</td><td>>500</td><td>|</td><td>>500</td><td>|</td><td>|</td><td>>500</td><td></td></tr>
<tr><td></td><td>48h</td><td>>500</td><td>>500</td><td>>500</td><td>>500</td><td>|</td><td>>500</td><td>|</td><td>|</td><td>>500</td><td></td></tr>
<tr><td>PA</td><td>72h</td><td>>500</td><td>>500</td><td>>500</td><td>>500</td><td>|</td><td>>500</td><td>|</td><td>|</td><td>>500</td><td></td></tr>
<tr><td></td><td>120h</td><td>>500</td><td>>500</td><td>>500</td><td>>500</td><td>|</td><td>>500</td><td>|</td><td>|</td><td>>500</td><td></td></tr>
</table>

* indicates a clinical isolate – see below

n.t. – compounds **28**, **30**, and **31** not tested due to heavy precipitation in the testing medium

Reference strains: SA - *Staphylococcus aureus* subsp. *aureus* CCM 4223 (ATCC 29213), MRSA – methicilin-resistant *Staphylococcus aureus* subsp. *aureus* CCM 4750 (ATCC 43300), EF - *Enterococcus faecalis* CCM 4224 (ATCC 29212), EC - *Escherichia coli* CCM 3954 (ATCC 25922), PA - *Pseudomonas aeruginosa* CCM 3955 (ATCC 27853)

Clinical isolates: SE - *Staphylococcus epidermidis* lab. id. 112-2016, KP - *Klebsiella pneumoniae* lab. id. 64-2016, SEMA *Serratia marcescens* lab. id. 62-2016

3.4. Antifungal screening of compounds of series C

Compounds **14**–**32** were screened for *in vitro* antifungal activity (Table S6). No activity was detected up to the highest tested concentration, which was dependent on the solubility of the compound.

Table S6. Results of antifungal activity screening of compounds **14**–**32**

**STRAIN** (code)		**COMPOUND** – **MIC** (µM)									
		**14**	**15**	**16**	**17**	**18**	**19**	**20**	**21**	**22**	**23**
**CA**	24h	>125	>500	>500	>500	>500	>500	>250	>250	>500	>500
	48h	>125	>500	>500	>500	>500	>500	>250	>250	>500	>500
**CK**	24h	>125	>500	>500	>500	>500	>500	>250	>250	>500	>500
	48h	>125	>500	>500	>500	>500	>500	>250	>250	>500	>500
**CP**	24h	>125	>500	>500	>500	>500	>500	>250	>250	>500	>500
	48h	>125	>500	>500	>500	>500	>500	>250	>250	>500	>500
**CT**	24h	>125	>500	>500	>500	>500	>500	>250	>250	>500	>500
	48h	>125	>500	>500	>500	>500	>500	>250	>250	>500	>500
**AF**	24h	>125	>500	>500	>500	>500	>500	>250	>250	>500	>500
	48h	>125	>500	>500	>500	>500	>500	>250	>250	>500	>500
**AFla**	24h	>125	>500	>500	>500	>500	>500	>250	>250	>500	>500
	48h	>125	>500	>500	>500	>500	>500	>250	>250	>500	>500
**AC**	24h	>125	>500	>500	>500	>500	>500	>250	>250	>500	>500
	48h	>125	>500	>500	>500	>500	>500	>250	>250	>500	>500
**TI**	72h	>125	>500	>500	>500	>500	>500	>250	>250	>500	>500
	120h	>125	>500	>500	>500	>500	>500	>250	>250	>500	>500

**STRAIN** (code)		**COMPOUND** – **MIC** (µM)									
		**24**	**25**	**26**	**27**	**28**	**29**	**30**	**31**	**32**	
**CA**	24h	>500	>500	>125	>125	│	>125	│	│	>125	
	48h	>500	>500	>125	>125	│	>125	│	│	>125	
**CK**	24h	>500	>500	>125	>125	│	>125	│	│	>125	
	48h	>500	>500	>125	>125	│	>125	│	│	>125	
**CP**	24h	>500	>500	>125	>125	│	>125	│	│	>125	
	48h	>500	>500	>125	>125	│	>125	│	│	>125	
**CT**	24h	>500	>500	>125	>125	n.t.	>125	n.t.	n.t.	>125	
	48h	>500	>500	>125	>125	│	>125	│	│	>125	
**AF**	24h	>500	>500	>125	>125	│	>125	│	│	>125	
	48h	>500	>500	>125	>125	│	>125	│	│	>125	
**AFla**	24h	>500	>500	>125	>125	│	>125	│	│	>125	
	48h	>500	>500	>125	>125	│	>125	│	│	>125	
**LC**	24h	>500	>500	>125	>125	│	>125	│	│	>125	
	48h	>500	>500	>125	>125	│	>125	│	│	>125	
**TI**	72h	>500	>500	>125	>125	│	>125	│	│	>125	
	120h	>500	>500	>125	>125	│	>125	│	│	>125	

n.t. – compounds **28**, **30**, and **31** not tested due to heavy precipitation in the testing medium

Reference strains: CA - *Candida albicans* CCM 8320 (ATCC 24433), CK - *Candida krusei* CCM 8271 (ATCC 6258), CP - *Candida parapsilosis* CCM 8260 (ATCC 22019), CT - *Candida tropicalis* CCM 8264 (ATCC 750), AF - *Aspergillus fumigatus* ATCC 204305, AFla - *Aspergillus flavus* CCM 8363, LC - *Lichtheimia corymbifera* CCM 8077, TI - *Trichophyton interdigitale* CCM 8377 (ATCC 9533)

3.5. Cytotoxicity to HepG2 cell line – viability dose-response curves

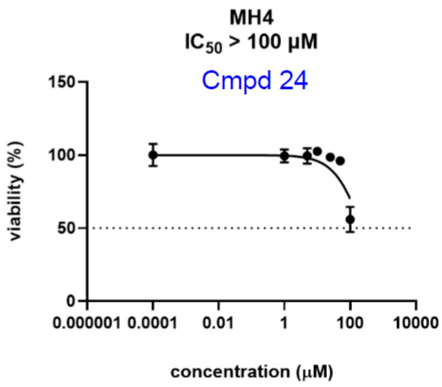

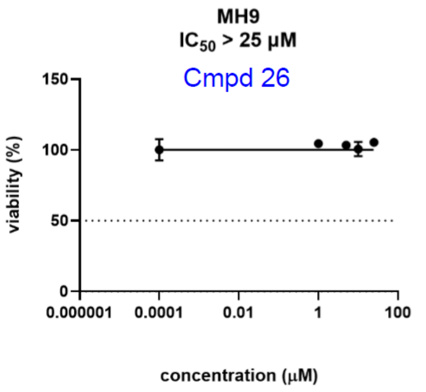

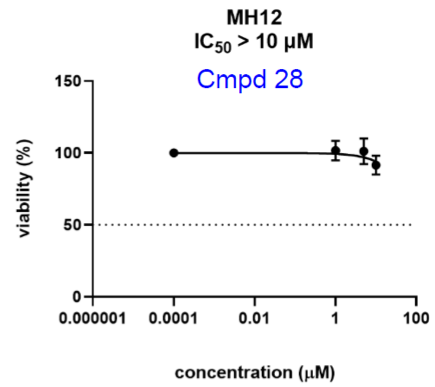

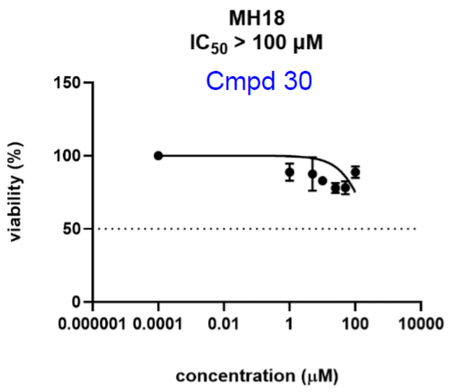

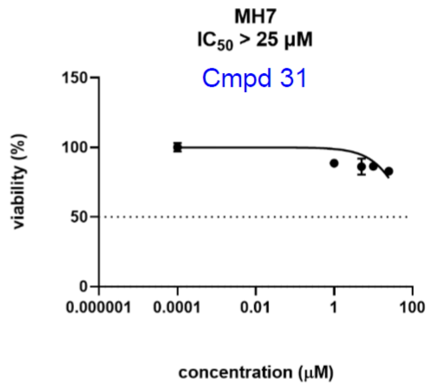

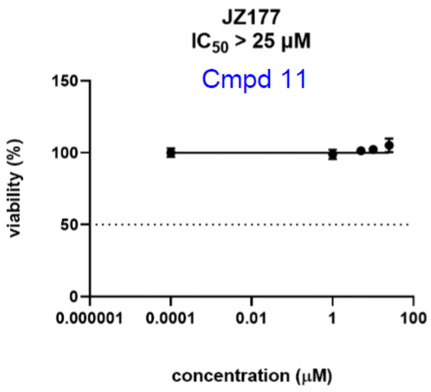

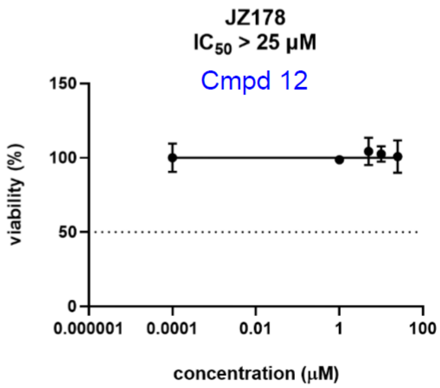

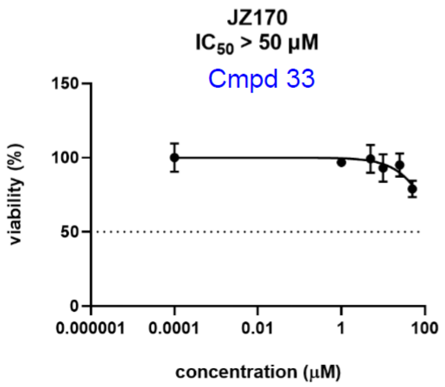

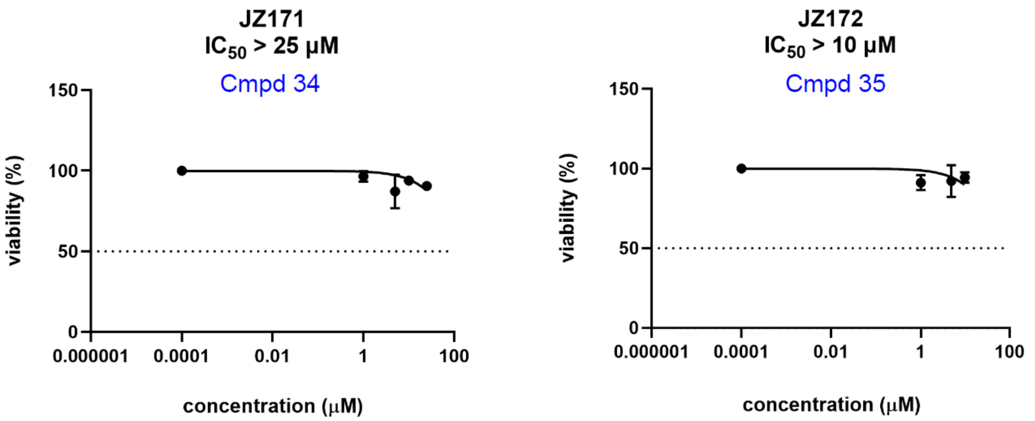

Figure S15. Dose-response curves and IC_{50} values from HepG2 cytotoxicity determination.

3.6. Molecular docking to mycobacterial enoyl-ACP-reductase (InhA)

Selected compounds (**15**, **20**, **31**) representative of series C were docked into the mycobacterial enoyl-ACP-reductase (InhA), PDB ID: 4R9S. The results indicated that the compounds could bind to the active site of InhA in a similar manner to the confirmed inhibitors. The carbonyl oxygen of the amidic linker acted as HBA (accepting a hydrogen from Tyr158 and a hydrogen from 2'-OH of the ribose of the NAD cofactor), and the alkyl chain was located in the entry tunnel, which is under normal situation occupied by the alkyl chain of the growing fatty acid intermediate. Figure S16 shows the docked poses of studied compounds compared to the crystallographic pose of PT70 (PDB ID: 2X23), a confirmed inhibitor of InhA and a close structural derivative of triclosan.

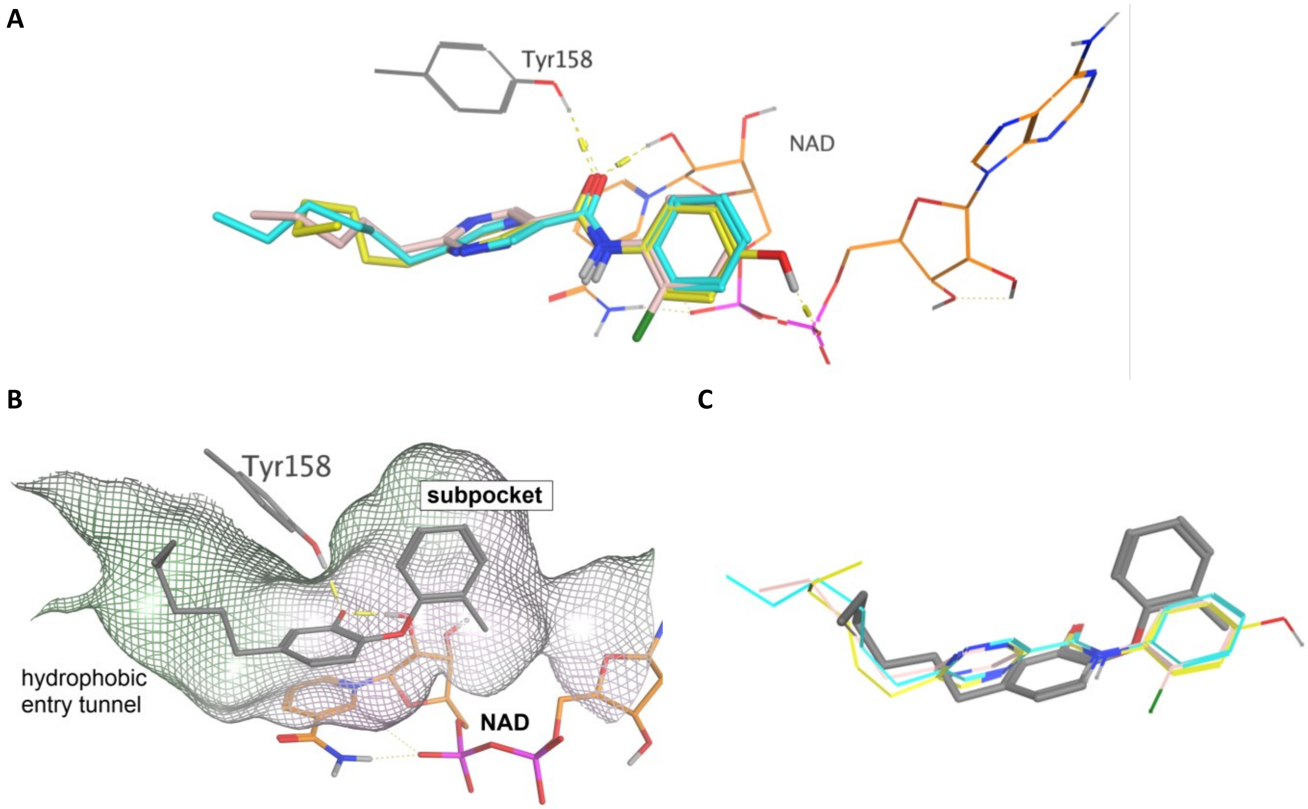

Figure S16. Docked poses of compounds **15** (cyan), **20** (salmon), and **31** (yellow) to mycobacterial enoyl-ACP-reductase (InhA), PDB ID: 4R9S. **A** – Overlay of the best-docked poses. **B** - Crystallographic pose of confirmed inhibitor PT70 (grey, PDB ID: 2X23), receptor surface in mesh colored by hydrophobicity (green hydrophobic, white neutral, violet hydrophilic). **C** – Comparison of the docked poses of the studied compounds with the crystallographic pose of PT70 (grey). NAD cofactor depicted with orange carbons.

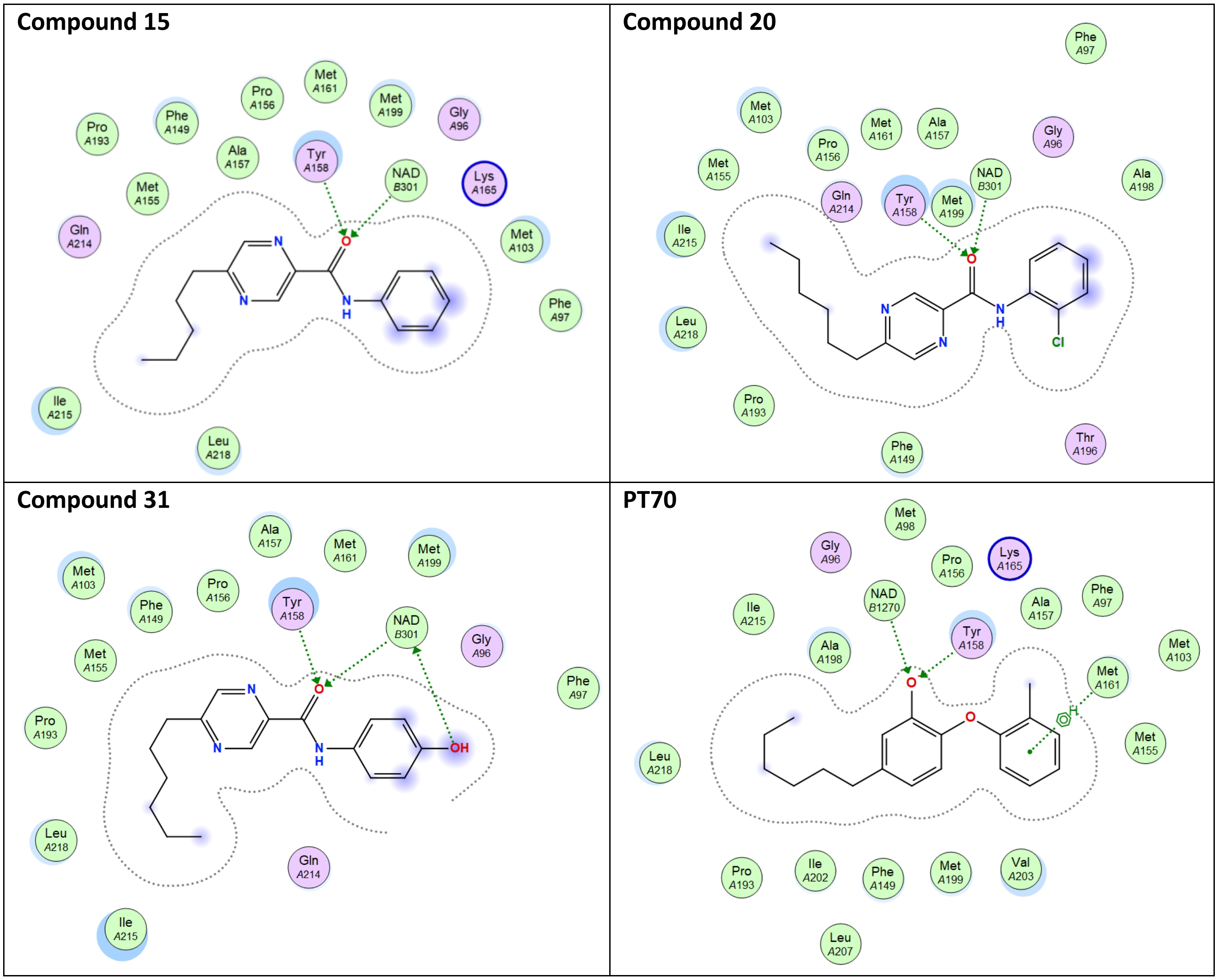

Figure S17. 2D ligand-protein interaction diagrams for poses from Figure S16.

3.7. Inhibition of enoyl-ACP reductase (InhA)

Table S7. Results of the inhibitory activity screening of the selected compounds against InhA. The inhibition is reported as % of the residual activity with the 95% CI.

Code	MW	InhA RA @ 10 µM (% [95% CI])	InhA RA @ 100 µM (% [95% CI])
**15**	269.35	99% [89–109]	89% [64–115]
**20**	317.82	103% [61–145]	N/A
**31**	299.37	99% [83–116]	83% [54-112]
Triclosan (standard)	289.54	37% [34–40]	Not tested

N/A – Not available due to precipitation in the testing medium

4. References

1. CLSI, *Susceptibility Testing of Mycobacteria,* Nocardia *spp., and Other Aerobic Actinomycetes*, 3 ed., Clinical and Laboratory Standards Institute, 2018.
2. European Committee for Antimicrobial Susceptibility Testing (EUCAST) of the European Society of Clinical Microbiology and Infectious Diseases (ESCMID), Determination of minimum inhibitory concentrations (MICs) of antibacterial agents by broth dilution, Clin. Microbiol. Infect., 9 (2003) ix-xv.
3. EUCAST DEFINITIVE DOCUMENT E.DEF 7.3.1., Method for the determination of broth dilution minimum inhibitory concentrations of antifungal agents for yeasts., in, 2017.
4. EUCAST DEFINITIVE DOCUMENT E.DEF 9.3.1., Method for the determination of broth dilution minimum inhibitory concentrations of antifungal agents for conidia forming moulds., in, 2017.
5. R. Šink, I. Sosič, M. Živec, R. Fernandez-Menendez, S. Turk, S. Pajk, D. Alvarez-Gomez, E.M. Lopez-Roman, C. Gonzales-Cortez, J. Rullas-Triconado, I. Angulo-Barturen, D. Barros, L. Ballell-Pages, R.J. Young, L. Encinas, S. Gobec, Design, Synthesis, and Evaluation of New Thiadiazole-Based Direct Inhibitors of Enoyl Acyl Carrier Protein Reductase (InhA) for the Treatment of Tuberculosis, J. Med. Chem., 58 (2015) 613-624.
